# Bayesian modeling of recombination events in bacterial populations

**DOI:** 10.1186/1471-2105-9-421

**Published:** 2008-10-07

**Authors:** Pekka Marttinen, Adam Baldwin, William P Hanage, Chris Dowson, Eshwar Mahenthiralingam, Jukka Corander

**Affiliations:** 1 Department of Mathematics and statistics, University of Helsinki, FIN-00014, Finland; 2 Department of Biological Sciences, Warwick University, Coventry. CV4 7AL, UK; 3 Department of Infectious Disease Epidemiology, Imperial College London. W2 1PG, UK; 4 Cardiff School of Biosciences, Cardiff University, Cardiff. CF10 3TL, UK; 5 Department of Mathematics, Abo Akademi University, FIN-20500, Finland

## Abstract

**Background:**

We consider the discovery of recombinant segments jointly with their origins within multilocus DNA sequences from bacteria representing heterogeneous populations of fairly closely related species. The currently available methods for recombination detection capable of probabilistic characterization of uncertainty have a limited applicability in practice as the number of strains in a data set increases.

**Results:**

We introduce a Bayesian spatial structural model representing the continuum of origins over sites within the observed sequences, including a probabilistic characterization of uncertainty related to the origin of any particular site. To enable a statistically accurate and practically feasible approach to the analysis of large-scale data sets representing a single genus, we have developed a novel software tool (BRAT, Bayesian Recombination Tracker) implementing the model and the corresponding learning algorithm, which is capable of identifying the posterior optimal structure and to estimate the marginal posterior probabilities of putative origins over the sites.

**Conclusion:**

A multitude of challenging simulation scenarios and an analysis of real data from seven housekeeping genes of 120 strains of genus *Burkholderia *are used to illustrate the possibilities offered by our approach. The software is freely available for download at URL .

## Background

Statistical approaches to investigating spatial heterogeneity within DNA sequences have attained a considerable interest for decades. However, the foci of such investigations have varied to a large extent from the analysis of spatially heterogeneous base compositions pioneered by works such as [1] and [2], to a kaleidoscope of methods for detecting anomalous evolutionary patterns caused e.g., by gene conversions, viral recombinations etc [3-7]. Here we focus on the statistical discovery of recombinant (homologous or non-homologous) segments within multilocus DNA sequences from bacteria representing heterogeneous populations of fairly closely related species. For a discussion of the various perspectives on the genomic evolution of bacteria, see, e.g. [8-11]. In this article, we use the word population rather loosely to describe a group of related bacteria. This group may correspond for example to a species, or a subgroup of species that is on its way to become a new species (see e.g. [10]). At some points we may also use the word population in another meaning to refer to all strains present in a data set (as when we speak of population structure). In such a situation populations within the population may be termed subpopulations. We expect that the meaning of the word should be apparent in any particular situation.

The recent trend among the statistical methods for evolutionary molecular biology is the upsurge of Bayesian methods, facilitated by the emergence of a class of powerful Markov chain Monte Carlo (MCMC) algorithms for fitting complex models to molecular data. Examples of such methods in the context of detecting recombination are [7,12-14]. Chan et al. [15] compared the performance of some popular methods for detecting recombination in a phylogenetic framework, and concluded that the Bayesian approach yielded accurate inferences. However, their simulation scenario was restricted to a four-taxon comparison, which is very simple in comparison with typical population genetic datasets. (For instance, [16] considered delineation of the population structure of several hundreds of bacterial strains from genus *Neisseria*).

When targeting to investigate the evolutionary relationships, the quality and informativeness of data as a representation of molecular variation in a population is of utmost importance. The prevailing situation regarding the applicability of the statistical methods is thus somewhat paradoxical, as the large data sets, which are the most representative and comprehensive, are beyond the reach of the currently available Bayesian methods. The approach in [14] where bacterial microevolution is considered in terms of recombination intensity, is capable of handling much larger data sets than the above-mentioned change-point models. However, it is not intended for making inferences about the origins of putatively recombinant segments, assuming instead that all recombination events introduce novel polymorphisms. To meet the above-stated challenges, we introduce here a novel statistical method for the detection of recombination events, including a probabilistic characterization of the uncertainty related to the origin of any particular site within the investigated DNA sequences. Our approach is based on a Bayesian spatial structural model representing the continuum of origins for multilocus sequences that has certain similarity with DNA segmentation models discussed in [17]. Bearing in mind the complex microevolutionary patterns typically observed in large-scale analyses of bacterial populations, we do not attempt to solve the inferential problem from an ordinary phylogenetic perspective, but utilize instead a recently introduced successful Bayesian framework for modeling genetic population structure. To enable a statistically accurate and practically feasible approach to the analysis of large-scale data sets representing a single genus, we have developed a novel software tool (BRAT, Bayesian Recombination Tracker), implementing the model and the corresponding learning algorithm, which is capable of identifying the posterior optimal structure and to estimate the marginal posterior probabilities of putative origins over the sites. The estimated structures can be efficiently explored using the built-in graphics options in BRAT.

A multitude of challenging simulation scenarios and an analysis of real data from seven housekeeping genes of 120 strains of genus *Burkholderia *are used to illustrate the potential of our approach. Our method assumes that a clustering of the strains to different populations is available. In our illustrations, we utilize the clustering of the strains obtained from BAPS (Bayesian Analysis of Population Structure) software, see e.g. [18,19]. Consequently, the simulations illustrate also the behavior of BAPS as an unsupervised classification tool for bacterial strain data.

The structure of the paper is as follows. *Methods *section is divided into three subsections: *Bayesian model for locating recombination events and their related origins *describes the model on a general level. *Details of the Bayesian model *contains the technical details of the model. *Estimation algorithms *outlines the algorithm for finding the optimal model structure. *Results *section is likewise divided into three subsections: *Coalescent simulation *– *a complete example, Repetitive phylogenetic simulation experiments *and *Burkholderia data *include illustrations of the method with both simulated and real data sets. In *Discussion *we summarize various aspects of the behavior and applicability of the model and compare the introduced method to some alternatives. Finishing remarks are provided in *Conclusions*.

## Methods

### Bayesian model for locating recombination events and their related origins

We consider a set of sampled bacteria for which aligned DNA sequences are available over *G *genes, indexed as *g*, * g *= 1, ..., *G*. The cardinality of the sampled set will be kept implicit in our notation to simplify it as much as possible. The observed DNA sequences will be denoted by **y**_*ig *_for any particular gene *g *and an individual strain *i *in the sample. Such data for a single strain, say *D*_*i*_, are unambigously represented by the integer vectors $Y_{ig}=(y_{ig1},...,y_{ign_{g}})\in {\left\{1,2,3,4\right\}}^{n_{g}}$, *g *= 1, ..., *G*, where the four integers correspond to a mapping from the set of bases {*A*, *C*, *G*, *T*} and *n*_*g *_is the length of the aligned sequence for the particular gene. We assume that there are no missing observations. Correspondingly, *D *refers to the complete set of observed data for all strains.

In order to achieve a detailed understanding of the gene flow in a population, it is necessary to establish both the presence of genetically separated subgroups inside it (and their configuration), as well as the individual ancestral patterns valid for the members of such subpopulations. A popular approach to modeling the genetic structure of a population is to use a Bayesian framework, where the number of putative genetically separated subpopulations is unknown *a priori*. Corander and Tang [18] derived a model for this purpose in the present setting, by extending the earlier work of [20] to linked molecular information. However, for large-scale data sets, it is not computationally practical, or often not even feasible, to learn within a single statistical model the genetic population structure, and simultaneously the detailed ancestry of each of the individuals at the finest possible level. At a coarser level, such as that represented by the commonly used admixture models (e.g. [19,21]), this is computationally challenging, but still manageable in practice.

Admixture models provide useful information concerning the evidence for the presence and absence of genetic barriers among various subsets of the data, and the average amount of putative recombinations that can have taken place to yield the molecular patterns present in the observed sequences. Nevertheless, while these models typically contain parameters that correspond to the percentage of the genotype deemed to originate in a specific ancestral group for each individual, they cannot directly pinpoint the locations in the sequences and thus assess the statistical uncertainty about them. The approach developed here can be considered as a complementary statistical tool to be utilized in conjunction with the methods introduced in [18] and [19], to achieve the latter goal of locating recombination events and assessing the uncertainty associated with them.

Assume that an estimate, say *S*, of the genetic population structure underlying the data *D *is available from the BAPS software implementing the various methods referred to (e.g. [18] and [19]). In a generic notation, such an estimate will contain samples from *K *underlying genetically separated groups *k*, *k *= 1, ..., *K*. Each of the *K *groups can now be putatively considered as the origin of any particular genomic segment present in *D*_*i*_. In addition to the putative ancestral origins identified in the unsupervised classification, we also consider explicitly the possibility that any particular DNA segment has its origin outside the investigated set of samples. In the sequel we let one of the *K *clusters corresponds to the outgroup, from which no reference samples are available, while the rest of the clusters correspond to the non-empty clusters detected in the clustering analysis.

The notation used in the sequel will treat the recombination events for each gene separately, conditional on the population structure *S*. In order to simplify the notation, the dependence of the proposed model on *S *will be kept implicit, whenever possible. However, we wish to emphasize that the statistical uncertainty about the molecular characteristics of each inferred subpopulation in *S *is taken into account by our model. Let *ρ *= (*ρ*_1_, ..., *ρ*_*m*_)be a *partition *or *segmentation *of the sequence **y**_*ig*_, defined by the set of breakpoints satisfying 0 = *ρ*_*o *_<*ρ*_1 _< ... <*ρ*_*m*-1 _<*ρ*_*m *_= *n*_*g*_, such that each *ρ*_*c*_, *c *> 0, is an integer determining the end point of a segment in the partition of **y**_*ig*_. If the number of segments *m *> 1, the actual set of sites belonging to the segment *c *in the partition is given by [*ρ*_*c*-1 _+ 1, *ρ*_*c*_], otherwise the whole sequence consists of a single segment (*m *= 1). Note that the number of segments *m *is considered unknown in our modeling framework, and it is one primary target for the statistical inference. In concrete terms, each *ρ*_*c *_in *ρ *specifies here a segment of a nucleotide sequence which has originated as a whole, either by binary fission or by recombination from one of the *K *putative ancestral sources.

Let *Z *= (*Z*_1_, ..., *Z*_*m*_) be a random vector specifying the origins for each of the *m *segments in *ρ*, i.e. *Z*_*c *_∈ {1, ..., *K*}. Thus, *Z *determines unambiguously the origin *X*_*j *_∈ {1, ..., *K*} for each site *j*, *j *= 1, ..., *n*_*g *_in **y**_*ig*_. Our inferential goals can now be specified as follows. Firstly, we seek to identify the partition of **y**_*ig *_and the origins of its segments, leading to an optimal probabilistic prediction for the observed sequence. This estimate corresponds to a pair (*ρ*, *Z*) maximizing the posterior distribution over the joint space of combinations of partitions and origin vectors. Secondly, we aim to quantify the uncertainty related to the estimation by providing marginal posterior probabilities for every *X*_*j*_, the origin of the *j*th base in **y**_*ig*._

To represent the nucleotide variation existing in a bacterial population due to mutation and recombination, our model incorporates parameters for the unknown relative frequencies of bases at each considered site, separately for each of the *K *subpopulations in *S*. These are determined by $p_{kjl}^{\left(g\right)}$, *g *= 1, ..., *G*, *k *= 1, ..., *K*, *j *= 1, ... *n*_*g*_, *l *= 1, ..., 4, where the last index corresponds to the four possible bases. Let *θ *denote jointly the set of all such parameters $p_{kjl}^{\left(g\right)}$ and *p*(*θ*) the corresponding distribution over the joint space of probability vectors Θ, describing the uncertainty remaining about *θ *after the qualitative genetic structure *S *has been learned. To make inferences about the structure (*ρ*, *Z*) of the sequence **y**_*ig*_, given all the observed data *D*, we utilize the posterior distribution of (*ρ*, *Z*), which can be written as

(1) $$\begin{array}{lll} p(\rho ,Z|D) & \propto & p(D|\rho ,Z)p(\rho ,Z)\\ & = & p(\rho ,Z)\int_{\Theta }p(D|\theta ,\rho ,Z)p(\theta |\rho ,Z)d\theta , \end{array}$$

where *p *(*ρ*, *Z*) is a prior distribution for the structure of the sequence and *p *(*D*|*θ*, *ρ*, *Z*) the likelihood of the observed sequence, conditional on the model parameters. The exact mathematical details of these model components are provided in the next subsection *Details of the Bayesian model*.

The analytical form of the posterior distribution (1) of (*ρ*, *Z*) derived in the next subsection enables computationally attractive ways of learning plausible ancestral structures, represented by (*ρ*, *Z*), for observed sequences, as discussed in the subsequent subsection *Estimation algorithms*. Recall that our second inferential goal is to provide an estimate for the marginal posterior probabilities of *X*_*j*_'s, i.e. the origins of all the bases. The probability of *X*_*j *_= *x*_*j *_can be estimated by summing the posterior probabilities (1) of all structural models (*ρ*, *Z*), for which this condition holds. Since it is computationally impossible to use a complete enumeration to treat all the possible models, and we wish to avoid a tedious MCMC analysis, we have developed an approach to choose those models which are the most relevant ones for calculating the marginal probabilities (see the next subsection).

### Details of the Bayesian model

Here we provide the mathematical details of the Bayesian estimation of the structural parameters (*ρ*, *Z*) for the sequence **y**_*ig*_. Because the analysis will be the same for all *i *and *g*, we will drop the indices here to simplify the notation, and use simply **y **= (*y*_1_, ..., *y*_*n*_)for the gene *g *of individual *i*. Similarly, *n *denotes the length of the gene *g*, *p*_*kjl *_the relative frequency of base *l *in population *k*, in site *j *of the gene *g*, etc.

Let S* denote a partition of the strains obtained from the original population structure *S *by removing the strain under investigation. Let *n*_*kjl *_denote the observed count of base *l *at site *j *in population *k *in S*, and *n*_*kj *_the total number of observations available from population *k *at site *j*. The likelihood term in the posterior (1) is defined as

(2) $$p(D|\theta ,\rho ,Z)=\prod_{k=1}^{K}\prod_{j=1}^{n}\prod_{l=1}^{4}p_{kjl}^{n_{kjl}+I(x_{j}=k\&y_{j}=l)}$$

Where *I *(*x*_*j *_= *k *&*y*_*j *_= *l*) is an indicator function, which equals unity if the *j*th site in **y **is assigned to the *k*th cluster and the base at the site equals *l*, otherwise *I*(*x*_*j *_= *k *&*y*_*j *_= *l*) is equal to zero. This form of the likelihood (2) corresponds to the assumption of conditional independence of the sites within a single gene *g*, given the population relative frequencies of the bases *θ*. More complex models formulating the linkage of the sites could also be used, such as the one introduced by [18]. However, the current form of the likelihood leads to a simplified computation. The conditional independence assumption is commonly utilized in Bayesian models, and works usually quite well, even if it may be unrealistic in practice [22].

Given an initial prior for the vector ${\left(p_{kjl}\right)}_{l=1}^{4}$ equal to the Dirichlet (*α*_1_, *α*_2_, *α*_3_, *α*_4_) distribution, the corresponding posterior for ${\left(p_{kjl}\right)}_{l=1}^{4}$ is also a Dirichlet distribution. Here we use *α*_*l *_= 1/4, *l *= 1, ..., 4, which leads to the following expression for the marginal likelihood

(3) $$\begin{array}{lll} p(D|\rho ,Z) & = & \int_{\Theta }p(D|\theta ,\rho ,Z)p(\theta |\rho ,Z)d\theta \\ & = & \prod_{k=1}^{K}\prod_{j=1}^{n}\frac{\Gamma \left(\sum_{l=1}^{4}\alpha_{l}\right)}{\Gamma \left(\sum_{l=1}^{4}\left(\alpha_{l}+n_{kjl}+I(x_{j}=k\&y_{j}=l)\right)\right)}\\ & \times & \prod_{l=1}^{4}\frac{\Gamma (\alpha_{l}+n_{kjl}+I(x_{j}=k\&y_{j}=l))}{\Gamma (\alpha_{l})}\\ & = & \prod_{k=1}^{K}\prod_{j=1}^{n}\frac{1}{\Gamma (1+n_{kj}+I(x_{j}=k))}\\ & \times & \prod_{l=1}^{4}\frac{\Gamma (1/4+n_{kjl}+I(x_{j}=k\&y_{j}=l))}{\Gamma (1/4)}. \end{array}$$

The specification of the posterior (1) still requires that we quantify prior probabilities for the underlying sequence structure

(4) *p *(*ρ*, *Z*) = *p *(*Z*| *ρ*) *p *(*ρ*).

Firstly, conditional on the partitioned sequence, we consider all combinations of segment origins to be equally likely, which leads to

(5) *p *(*Z *= *z*|*ρ*) = *K*^-*m*^.

Secondly, there are two characteristics to be met by the prior distribution *p *(*ρ*). The prior should be relatively vague, in order to avoid strong influences towards the locations and abundance of recombination events, and also, it should lead to computationally tractable solutions. An immediate candidate for such a prior would be the uniform distribution over all possible partitions of the sequence. However, such a prior would give too much weight to partitions in which some, or many of the segments are only one or few bases long. This is not reasonable from a biological perspective, as the recombinant sequences could then be reduced to point mutations in the most extreme configurations. Also, from the statistical perspective, the resulting partitions could easily reflect unidentifiable models. To resolve this issue, we utilize a prior defined by

(6) *p*(*ρ*) = *C *× *I*(*ρ*),

where *C *is a constant and *I*(·) is an indicator function, such that

$$I(\rho )=\{\begin{array}{l} \begin{array}{ccc} 1, & \text{if }\rho_{c}-\rho_{c-1}\geq L, & \forall c=2,...,m-1 \end{array}\\ \begin{array}{cc} 0, & \text{otherwise}. \end{array} \end{array}$$

Thus, the prior (6) is uniform over all partitions, which do not contain any segments shorter than *L*, except that the first and the last segments are allowed to be of any length due to computational simplicity and also the obvious biological fact that in practice the recombined segments may continue beyond the endpoints of a gene.

Sequence data usually contain quite limited information for formal learning of the parameter *L*. Therefore, we have chosen a strategy of using a fixed value *L *= 15, which was considered reasonable from the biological point of view. In general, we concluded that as long as *L *is not too small, the results are quite robust with respect to the exact value of *L *(see supplementary material in Additional file 1). If *L *is too small, say less than 5, then local features may have strong influence on the calculated marginal posterior probabilities (see below). This undesired behavior was actually the main reason why the prior (6) excluding unrealistically small segments was selected. The above derived analytical form of the kernel function of the posterior distribution (1) of (*ρ*, *Z*) now enables computationally attractive ways of learning plausible ancestral structures for observed sequences.

As stated earlier, our second inferential goal is to provide an estimate for the marginal posterior probabilities of *X*_*j*_'s, i.e. the origins of all the bases. The probability of *X*_*j *_= *x*_*j *_can be estimated by summing the posterior probabilities (1) of all structural models (*ρ*, *Z*) for which this condition holds. Since it is computationally impossible to use complete enumeration to treat all possible models, and we wish to avoid a tedious MCMC analysis, we have developed an approach to choose those models which are the most relevant for calculating the marginal probabilities. For this purpose, some new notation is necessary to be introduced. Let *ρ*_[*a*, *b*] _denote a partition induced by *ρ *for the interval [*a*, *b*] of bases, i.e., for all *j*, *j' *such that a *a *≤ *j*, *j' *≤ *b*, *j *and *j' *belong to the same segment in *ρ*_[*a*, *b*]_, if and only if they belong to the same segment in *ρ*. Analogously let *Z*_ [*a*, *b*] _be the vector of origins for the segments in *ρ*_[*a*, *b*]_, induced by *Z*. In the sequel we denote the subset of data *D *corresponding to positions [*a*, *b*] by *D*_ [*a*, *b*]_.

The marginal posterior probabilities of origin for any single site are defined by

(7) $$p(X_{j}=x_{j}|D)=\frac{\sum_{(\rho ,Z):X_{j}=x_{j}}p(D|\rho ,Z)p(\rho ,Z)}{\sum_{\left(\rho ,Z\right)}p(D|\rho ,Z)p(\rho ,Z)}$$

Using the assumed conditional independence of the values of the bases, given the structural parameters, the probabilities in the numerator can be expressed using the following factorization:

(8) $$\begin{array}{ll} & \sum_{(\rho ,Z):X_{j}=x_{j}}p(D|\rho ,Z)p(\rho ,Z)\\ = & \sum_{\begin{array}{c} {}[a,b]∋j,\\ b-a+1\geq L \end{array}}p(D_{\left[a,b\right]}|\text{segment }[a,b]\text{ emanates from }x_{j})K^{-1}\\ \times & \sum_{\begin{array}{c} \rho_{\left[1,a-1\right]},\\ Z_{\left[1,a-1\right]} \end{array}}p(D_{\left[1,a-1\right]}|\rho_{\left[1,a-1\right]},Z_{\left[1,a-1\right]})K^{-|\rho_{\left[1,a-1\right]}|}\\ \times & \sum_{\begin{array}{c} \rho_{\left[b+1,n\right]},\\ Z_{\left[b+1,n\right]} \end{array}}p(D_{\left[b+1,n\right]}|\rho_{\left[b+1,n\right]},Z_{\left[b+1,n\right]})K^{-|\rho_{\left[b+1,n\right]}|},\\ = & \sum_{\begin{array}{c} {}[a,b]∋j,\\ b-a+1\geq L \end{array}}p(D_{\left[a,b\right]}|\text{segment }[a,b]\text{ emanates from }x_{j})\\ \times & \begin{array}{cc} K^{-1}p(D_{\left[1,a-1\right]}) & p(D_{\left[b+1,n\right]}) \end{array} \end{array}$$

The interpretation of (8) is that instead of summing directly over all possible partitions, we take the outmost sum over all possible segments including the *j*th position, and outside that segment, we still sum over all possible ways of partitioning the sequence. (If the *j*th position is closer to the endpoints of the gene than *L *bases, some of the possible segments including the site may be shorter than *L*. For simplicity, the derivation is not shown for these special cases.) Because of the assumed conditional independence of the sites,

$$p(D_{\left[a,b\right]}|\text{segment }[a,b]\text{ emanates from }k)=\prod_{r=a}^{b}p(D_{\left[r,r\right]}|r\text{ emanates from }k),$$

we see that (8) is a sum of products, such that *p *(*D*_[*j*, *j*]_| *j *emanates from *x*_*j*_) is a factor in all of the terms, and the closer *i *is to *j*, the more terms in the sum have the marginal likelihood of the *i*th site, *p*(*D*_ [*i*, *i*]_*| i *emanates from *x*_*j*_), as a factor. Thus, because *K*^-1 ^*p *(*D*_[1, *a*-1]_) *p*(*D*_[*b*+1, *n*]_) in (8) does not depend on *x*_*j*_, we conclude that *p *(*X*_*j *_= *x*_*j*_*|D*) is mostly determined by the sequence positions close to *j*.

This motivates the following approximation for (8):

(9) $$\sum_{\begin{array}{c} {}[a,b]∋j,\\ L\leq b-a+1\leq L_{\max} \end{array}}p(D_{\left[a,b\right]}|\text{segment }[a,b]\text{ emanates from }x_{j})K^{-1}p(D_{\left[1,a-1\right]})p(D_{\left[b+1,n\right]})$$

where *L*_max _is a sufficiently large integer to provide reasonable accuracy for the approximation. Thus, *L*_max _specifies the distance along the gene such that observed values further than *L*_max _from site *j *have no direct effect on the calculated marginal posterior probabilities for site *j *(these observations may yet affect the weights for different intervals considered in (9) by affecting *p *(*D*_ [1, *a*-1]_) and *p *(*D*_ [*b*+1, *n*]_)). Thus, the exact value of *L*_max _has some effect on the calculated marginal posterior probabilities by determining a region in the sequence from which the evidence is summarized by the marginal posterior probability distribution for origins of site *j *(see an illustration in the supplementary material in Additional file 1). To derive an appropriate value for *L*_max _in practice, we calculate a value using the criterion

(10) $$L_{\max}=\min\{x\in {\mathbb{N}}^{+}:p("\text{length of interval containing }j"\leq x)>0.99\},$$

where *p *is calculated using the prior probability for the partitions. *L*_max _is calculated in practice using the recursive procedure described at the end of this section.

Since an exhaustive enumeration of the possible partitions for the regions outside of the segment [*a*, *b*] in (9) is computationally impossible, we will utilize an approximation for the values of *p *(*D*_ [1, *a*-1]_) and *p *(*D*_ [*b*+1, *n*]_). Bayesian asymptotics, see, e.g. [23], show that

(11) $$p(D_{\left[1,a-1\right]})\approx p\left(D_{\left[1,a-1\right]}|{\hat{\rho }}_{\left[1,a-1\right]},{\hat{Z}}_{\left[1,a-1\right]}\right)p\left({\hat{\rho }}_{\left[1,a-1\right]},{\hat{Z}}_{\left[1,a-1\right]}\right)$$

when the amount of data increases, i.e. the sum of marginal likelihoods will be dominated by the single term corresponding to the optimal model $\left({\hat{\rho }}_{\left[1,a-1\right]},{\hat{Z}}_{\left[1,a-1\right]}\right)$ for the interval [1, *a *- 1]. We simplify (11) further by writing

(12) $$\begin{array}{l} p\left(D_{\left[1,a-1\right]}|{\hat{\rho }}_{\left[1,a-1\right]},{\hat{Z}}_{\left[1,a-1\right]}\right)p\left({\hat{p}}_{\left[1,a-1\right]},{\hat{Z}}_{\left[1,a-1\right]}\right)\\ \propto p\left(D_{\left[1,a-1\right]}|\text{segment }[1,a-1]\text{ emanates from }{\hat{k}}_{\left[1,a-1\right]}\right)p\left([1,a-1]\text{ from }{\hat{k}}_{\left[1,a-1\right]}\right), \end{array}$$

where ${\hat{k}}_{\left[1,a-1\right]}$ denotes the single origin maximizing (1) for the segment [1, *a *- 1]. Thus, instead of using the globally optimal model for interval [1, *a *- 1], we use a model in which the segment [1, *a *- 1] has been assigned as a whole to one optimal origin. In practice the effect of approximation (12) usually cancels out. Suppose that some part of [1, *a *- 1] should be from another origin than ${\hat{k}}_{\left[1,a-1\right]}$. Then, using the correct model would increase the marginal likelihood *p *(*D*_ [1, *a*-1]_) in (9) by some factor. However, the value of *p *(*D*_ [1, *a*-1]_) acts as a factor in the sum (9) in a term corresponding to interval [*a*, *b*] also for a competing origin, say $x_{j}^{*}$, and thus the approximation treats the competing origins equally. Furthermore, because we are only considering the surroundings of *j *up to a distance of *L*_max_, possible recombinant segments more distant than *L*_max _bases from *j *would affect *p *(*D*_ [1, *a*-1]_) for all of the terms in the sum (9) for any origin *x*_*j*_, and thus cancel out. In supplementary material (Additional file 1) we illustrate the error caused by the approximations (11) and (12), and conclude it to be negligible in practice. The probability *p*(*D*_ [*b*+1, *n*]_) is approximated analogously to *p*(*D*_ [1, *a*-1]_). Because the models considered in the approximation all consist of three segments, and thus have equal priors, our final version of the required marginal probability can be written as

(13) $$\begin{array}{lll} p(X_{j}=x_{j}|D) & \propto & \sum_{\begin{array}{c} {}[a,b]∋j\\ L\leq b-a+1\leq L_{\max} \end{array}}p(D_{\left[a,b\right]}|\text{segment }[a,b]\text{ emanates from }x_{j})\\ & \times & p\left(D_{\left[1,a-1\right]}|\text{segment }[1,a-1]\text{ emanates from }{\hat{k}}_{\left[1,a-1\right]}\right)\\ & \times & p\left(D_{\left[b+1,n\right]}|\text{segment }[b+1,n]\text{ emanates from }{\hat{k}}_{\left[b+1,n\right]}\right). \end{array}$$

All the terms in (13) can be evaluated analytically using an expression corresponding to one segment in (3)

$$\begin{array}{lll} p(D_{\left[a,b\right]}|\text{segment }[a,b]\text{ emanates from }x) & = & \prod_{k=1}^{K}\prod_{j=a}^{b}\frac{1}{\Gamma (1+n_{kj}+I(x=k))}\\ & \times & \prod_{l=1}^{4}\frac{\Gamma (1/4+n_{kjl}+I(x=k\&y_{j}=l))}{\Gamma (1/4)}. \end{array}$$

To derive an appropriate value for *L*_max _in (10), we utilize the following recursive procedure under the specified prior distribution (6) for the sequence partitions. Let *A*(*t*) denote the number of partitions of a sequence of length *t*, such that all segments, except possibly the first, are at least of length *L *= 15. For small values of *t *we have

*A*(*k*) = 1, for *k *= 1, ..., 15.

Further, for *t *= 16, ..., *n*, where *n *denotes the length of the gene, *A*(*t*) may be written using a recursive formula as

$$A(t)=1+\sum_{j=1}^{t-15}A(j),$$

where [*j *+ 1, *t*] is a segment and the sum is over all possible locations *j *for the breakpoint preceding *t*. Thus, the number of partitions in which [*a*, *b*] is one segment is given by

$$N_{\left[a,b\right]}=\{\begin{array}{l} 1,\text{ if }a=1\text{ and }b=n,\\ A(n-b),\text{ if }a=1\text{ and }b<n,\\ A(a-1),\text{ if }a>1\text{ and }b=n,\\ A(a-1)*A(n-b),\text{ otherwise}. \end{array}$$

Consequently, the sum of prior probabilities of partitions in which [*a*, *b*] is one of the segments, is given by

(14) $$\frac{N_{\left[a,b\right]}}{\sum_{j=n-14}^{n}A(j)},$$

where the denominator is the number of all partitions with positive prior probability (notice that in such a partition both the first and the last segment are allowed to be of any length). The prior probability that the length of the segment containing *j *equals *t *is obtained by summing values given by (14) for all [*a*, *b*], which contain *j *and are of length *t*. For instance, for gene lengths between 300–450 bases, the prior probability that a segment containing *j *is shorter than 56, is at least 0.99, which in this case provides us a value for *L*_max _in (10).

### Estimation algorithms

Standard MCMC algorithms, such as the Gibbs sampler or Metropolis-Hastings algorithm have generally been adopted as tools of choice for fitting Bayesian models to data [24]. However, it is generally acknowledged in statistical literature that numerical convergence and mixing problems for such methods are burdening their application to complex models. Therefore, a myriad of methods have been developed to solve various problems arising in the practical applications, see, e.g. [24]. Particularly challenging classes of Bayesian learning problems are represented by situations where the model dimensionality is not fixed *a priori *(see, e.g. [25]), as well as general combinatorial optimization tasks [26].

Our sequence structure model introduced in the previous subsections has the necessary characteristics to enable estimation of the posterior using the general parallel non-reversible Metropolis-Hastings algorithm introduced by [27]. A central feature of the algorithm is the possibility to utilize intelligent stochastic search operators for which proposal probabilities cannot be calculated in a closed form. Corander et al. demonstrated that already with relatively simplistic random search operators the non-reversible algorithm outperformed a comparable reversible algorithm for fitting a Bayesian unsupervised classification model to a large bacterial database. Nevertheless, as concluded by [28], despite of its advantages, the parallel non-reversible algorithm is still very demanding computationally on a single CPU architecture for complex models. As this limitates the application in practice, [28] developed a stochastic greedy optimization algorithm that utilizes intelligent search operators both locally and globally to identify model structures associated with high posterior probabilities. Here we exploit an analogous approach to optimize the partition of the sequence and origins for different clusters based on (13).

To enhance the search for the optimal structure, we identify first a candidate structure for the sequence by using the calculated marginal probabilies (13) to assign each base to the origin with the highest marginal probability. Although this procedure may lead to a non-legitimate initial model structure with some segments having length smaller than *L *(*L *is the minimum segment length allowed by the model, see the previous subsection), these are merged later in the actual search procedure to yield only models with positive prior probabilities.

Given an initial model configuration, our search for the posterior optimum uses the following steps:

1. Identify the two adjacent segments of different origins for which an assignment to the same origin yields the largest improvement in the posterior probability of the structure. Repeat such assignments until no improvement can be obtained for the posterior probability.

2. For all *c *= 1, ..., *m *- 1, where *m *is the current number of segments, identify the location of the breakpoint *ρ*_*c *_associated with the highest posterior probability for the subsequence from *ρ*_*c*-1 _+ 1 to *ρ*_*c*+1_.

3. If the current partition contains segments with length <*L*, each of them is merged to an adjacent segment leading to largest posterior probability among the two alternatives.

The three above steps are repeated in our algorithm until no improvement is obtained for the posterior probability. In practice the algorithm converges very rapidly, and furthermore, partitions with one or more segments having length <*L *(*L *= 15) occurred very seldom after the second step in our computational experiments with both real and simulated data. The reason for this can be seen from the analytical form of the marginal likelihood conditional on the structural parameters, as it is unlikely that such a short interval would contain enough information to compensate for the increased uncertainty related to the parameters specifying the origin of the interval (this uncertainty is a result of the uniform prior over possible origins, and leads to penalty 1/*K *in (1) when a novel segment is added). Thus, the last search operator rather guarantees under such unlikely events that all considered states are legitimate, i.e. having strictly positive posterior probabilities. As with greedy search algorithms in general, no guarantee can be given that the search really finds the globally optimal model. Local modes are more commonly encountered in a situation with a lot of uncertainty. This uncertainty is in our approach reflected by the marginal posterior probabilities of the origins of the sites. Thus, if these probabilities show a considerable amount of uncertainty, it is likely that the model space contains models which are approximately equally good descriptions of the data, and the search finds only one of the alternatives. In such a situation the uncertainty related to the estimated model should anyway be taken into account when interpreting the results. Notice also that although the parameters *L *and *L*_max _do not have a direct effect on the optimal model, they may have an indirect effect on the estimated optimal model in a situation with a lot of uncertainty, because they have some effect on the marginal posterior probabilities which are used to define the starting point for the search (see illustration concerning *L *and *L*_max _in the supplementary material, Additional file 1).

In practice the number of putative origins of a segment in a data set may be very large. However, it is not feasible to consider all of them as equally likely candidates in the segmentation model, because this would make it impossible to produce any meaningful graphical presentation of the results. To account for the uncertainty relevant for most practical situations, we restrict the maximum number of putative origins to equal 10 for any single sequence. The most plausible origins are detected automatically by our software implementation for the investigated strain. We have implemented the automated selection of the putative origins as follows. Firstly, the most important putative origin equals the subpopulation into which the investigated strain was allocated in the genetic clustering suggested in the previous sections as the first phase of the recombination analysis. Secondly, an empty cluster should always be included to account for the events where a segment has an ancestral source outside the subpopulations present in the current sample. To select the remaining eight origins, we scan through all the segments of length 50 bases and select all those subpopulations which have the highest predictive likelihood in some segment. If less than eight clusters are selected in this way, additional clusters will be chosen, based on the predictive likelihood for the whole sequence. If more than eight clusters are selected, those of the selected clusters which have the lowest predictice likelihood for the whole sequence, will be removed. The resulting group of ten clusters will be used in the analysis.

## Results

### Coalescent simulation – a complete example

We next illustrate our method with a realistic synthetic data set, created by using a software Recodon [29], which is able to generate samples of codon sequences from populations with recombination, migration and demography, using a coalescent. Recodon allows the user to specify several parameters defining the properties of the simulation. The values of different parameters can be found in the parameter file (in supplementary material, Additional file 1) which we provided as an input for the software.

**Figure 1:**
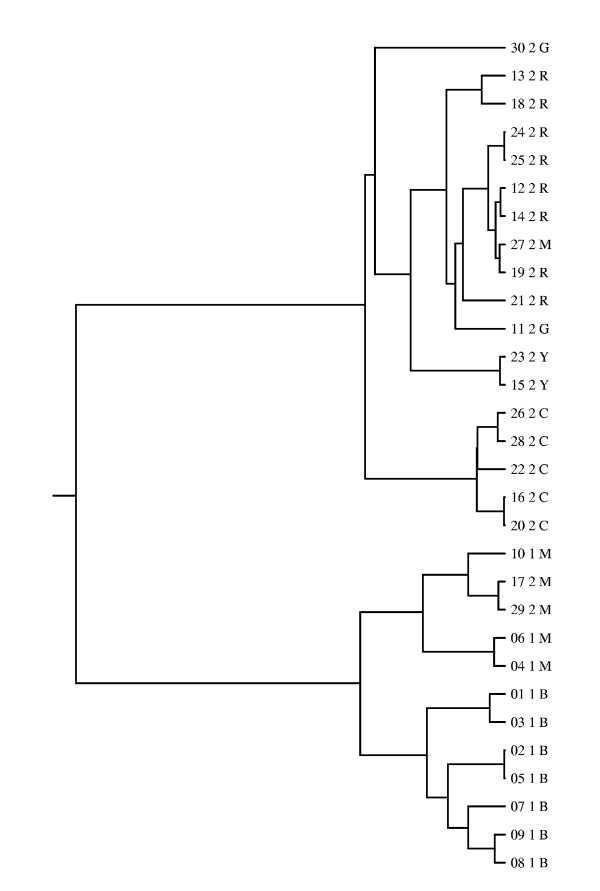
**Evolutionary history for the second fragment in the coalescent simulation**. Evolutionary tree for the second fragment in the coalescent simulation. The leaf nodes correspond to the strains in the data set, and are labelled as XX Y Z, where XX is strain index, Y is the deme to which the strain belonged in the simulation and Z is the cluster label for the strain from BAPS analysis. The clusters are labelled by different colors as follows: R = red, G = green, B = blue, Y = yellow, M = magenta, C = cyan.

The simulated data set consists of genes of length 303 bp for a set of 30 strains. A genetic mixture analysis of these data with BAPS software yielded six clusters. These clusters represent various levels of heterogeneity, such that the average distances among the members of a single cluster are in the interval from 3 to 38 bp, whereas the average distances between different clusters range from 37 to 75 bp. As it is not feasible to present here the complete results, some interesting features are highlighted for three selected strains, and the complete results are shown in the supplementary material (Additional file 1). Results for the strains selected for illustration show features which can be expected to be observed in typical data sets. The simulation process created a total of 11 recombination breakpoints, and hence, the strains consist of 12 gene fragments with differing evolutionary histories. The evolutionary trees for the fragments were obtained from Recodon output. We used Drawgram program included in Phylip software package [30] to draw the trees. An example of such a tree is shown in Figure 1 for the second gene fragment. Remaining trees are provided in the supplementary material. In general, the clustering obtained with BAPS correlates strongly with the true evolutionary histories of the fragments. This is also apparent in Figure 1, with the following two exceptions. Firstly, strain #27, which is assigned to the magenta-colored cluster, is with respect to this fragment very closely related to the strains of the cluster labeled by the red color. Secondly, strains #11 and #30, which form the cluster labeled by the green color, are not very closely related to each other.

**Figure 2:**
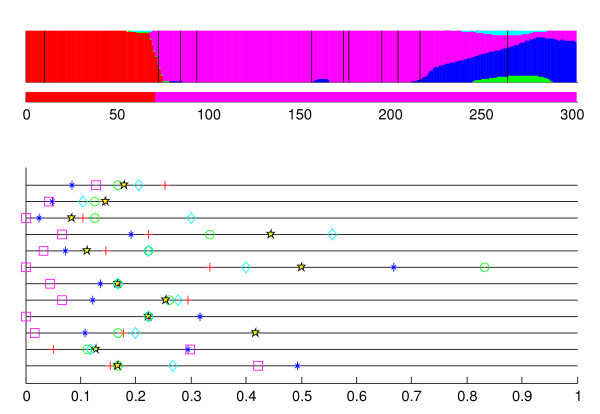
**Coalescent strain #27**. The results for strain #27 in the coalescent simulation. The results obtained from a recombination analysis with BRAT are shown in two colored plots in the upper part of the figure, a narrow plot below a wider plot. In these plots, the different colors correspond to the clusters obtained in the unsupervised mixture classification analysis. The x-axis position specifies a site in the analyzed gene. The narrow plot (optimal model profile) displays the detected optimal model, where each base is assigned to an origin specified by the optimal model. The wider plot (marginal probability profile) shows graphically for each site *j *in the sequence the marginal posterior probability distribution for *X*_*j*_, *j *= 1, ..., *n*_*g*_, the origin of the *j*th base in the gene. The true breakpoint locations are shown by vertical black lines in the marginal probability profile. The plot in the lower part of the figure reflects the correct solution and shows fragmentwise molecular distances from this strain to the identified clusters. Horizontal lines correspond linearly to the different fragments, such that the lowest horizontal line corresponds to the first fragment, etc. The clusters are represented by cluster-specific colored markers. The distance of the marker from the Y-axis shows the average distance between the corresponding cluster and the strain in the corresponding fragment. The distances are normalized according to the lengths of the gene fragments.

As our first example, we investigate more closely the strain #27. The results of recombination inference for this strain given the BAPS clustering are shown in Figure 2. The results of the recombination analysis are summarized by two plots, marginal probability profile, which shows graphically the posterior distributions for *X*_*j*_, and optimal model profile, which shows the optimal partition with each segment assigned to the optimal origin. We will refer to these two profiles jointly as a recombination profile. Figure 2 also displays for each gene fragment the average molecular distances between strain #27 and the strains in the six identified clusters. Recalling that the strain was assigned to the magenta-colored cluster in BAPS clustering, this cluster is indeed associated with high values in the marginal probability profile for the fragments 3–10. Also, for all these fragments, the magenta-colored cluster is the closest one in terms of sequence similarity, which can be seen from the lower pane of Figure 2. An inspection of the evolutionary histories of these gene fragments (supplementary material) verifies that the strain #27 is most closely related to other strains of the magenta-colored cluster. The optimal segmentation model assigns the first two gene fragments to the red-colored cluster, supported by conclusive marginal probabilities. As already seen in Figure 1, the second fragment of the strain #27 is really evolutionarily linked to the strains of the red-colored cluster, and the same result holds for the first gene fragment (supplementary material).

The last two gene fragments are in the optimal segmentation model assigned to the magenta-colored cluster. However, the marginal probability profile associates also the blue-colored cluster with high values. An inspection of the distances in Figure 2, reveals that the magenta- and blue-colored clusters are roughly equally distant from the strain #27 in the second last fragment, and furthermore, that in the last fragment this strain resembles most the members of the blue-colored cluster. The close relationships of the strain #27 with the strains in magenta- and blue-colored clusters can similarly be seen in the evolutionary histories for these fragments.

**Figure 3:**
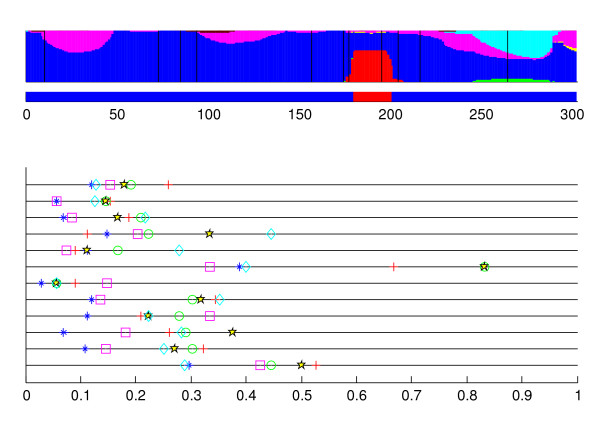
**Coalescent strain #3**. The results for the strain #3 in the coalescent simulation.

Our second and third examples illustrate cases where the optimal model suggests a recombination event which is not supported by the evolutionary histories of the gene fragments. Figure 3 displays the results of recombination inference for the strain #3, for which a gene segment (sequence positions 181–201) is assigned to the red-colored cluster by the optimal model. This recombination event can not be identified in the evolutionary histories of the corresponding fragments. However, a closer investigation reveals that in this segment the strain #3 is in fact identical to all the strains in the red-colored cluster, whereas it has on average 3.3 bp differences to the other strains allocated to the blue-colored cluster (the most plausible one in general). Thus, the recombination suggested by the optimal model corresponds here to a region in which mutations are by chance accumulated in an unexpectedly short interval.

**Figure 4:**
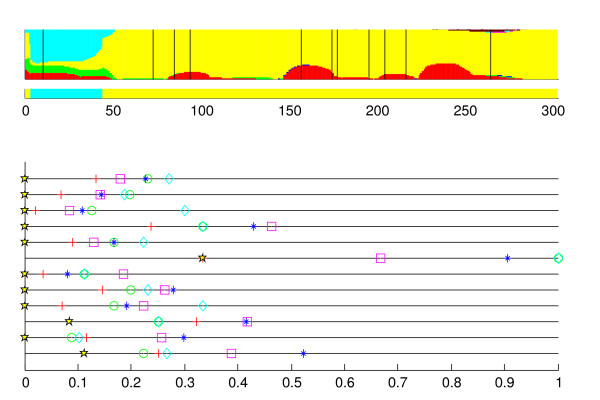
**Coalescent strain #15**. The results for the strain #15 in the coalescent simulation.

The third example is shown in Figure 4, where the results from recombination inference are shown for the strain #15. It can be concluded that the yellow-colored cluster is located closest to this strain in all the gene fragments. However, there is still a short segment overlapping the first and the second fragments, which is considered as recombinant in the optimal model. This is explained by the fact that, apart from the strain #15, there is only one additional strain in the yellow-colored cluster. Therefore, there is considerable uncertainty regarding the nucleotide frequencies associated with the cluster. Consequently, the cluster may be assigned fairly low marginal posterior probabilities, if there exists at least a single alternative cluster with the characteristic that its strains are equally dissimilar to the considered strain. In the segment identified as putatively recombinant by the optimal model, the cyan-colored cluster is least dissimilar to the strain #15, with an average distance of 0.2 bps, whereas the other strain in the yellow-colored cluster differs by one nucleotide.

The second and the third examples illustrate potential causes of a phenomenon which can be considered as false positive recombination discovery. However, it should be stressed that the putatively recombinant segment in the optimal model was not conclusively supported by the marginal posterior probabilities in neither of these cases, as opposed to the first example where the true recombination event was identified. In particular, it is important when interpreting the results that the conclusions based on the optimal recombination profile reflect the uncertainty present in the marginal probability profile. It is also worth noticing that in these cases the suggested recombinant segments were relatively short (31 bp or less). This feature we have observed also more generally to hold in similar situations. To aid the interpretation of the estimation results, our software implementation of the method (BRAT) provides an immediate access to the average distances with respect to the different clusters for an arbitrary selected sequence region, as well as to levels of molecular variation within the clusters.

### Repetitive phylogenetic simulation experiments

As the analysis of the coalescent data set in the previous subsection illustrated, the behavior of the introduced method depends on the characteristics of any particular data analysis situation. These characteristics include for example the genetic distance and sizes of the populations involved in the recombination event.

In this subsection we investigate more closely five different types of scenarios, which cover the most important types of characteristics expected to be present in a molecular data set. These scenarios are: 1) recent recombination between distantly related strains, 2) recent recombination between closely related strains, 3) old recombination, such that the recombined fragment is present in all members of a population, 4) recombination event involving a population with only a very limited number of members (miniature cluster), and 5) recombination where the recombinant fragment is not acquired from any of the populations present in the observed data. Each of these five situations is further investigated under three different levels of molecular variation.

**Figure 5:**
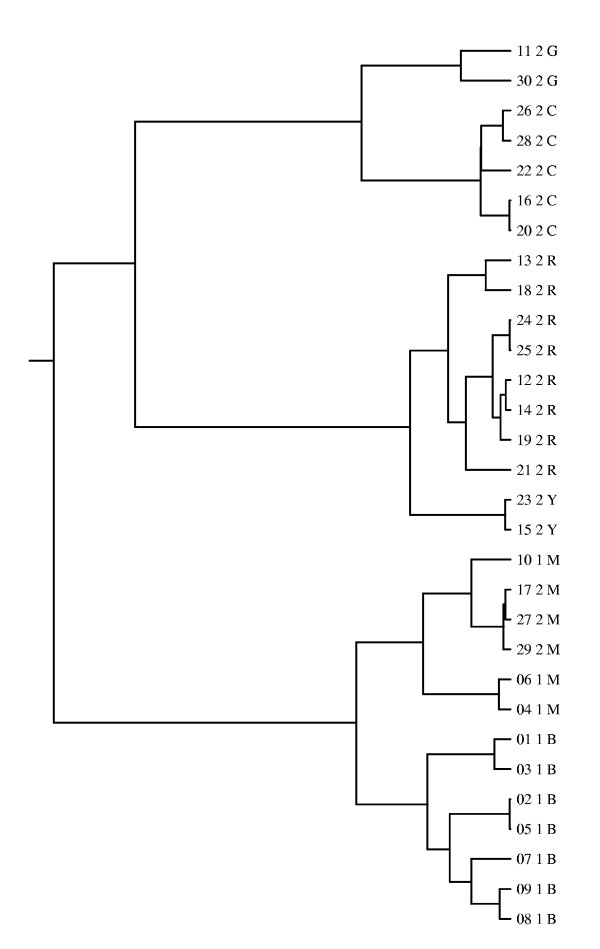
**Evolutionary history for the 6th fragment in the coalescent simulation**. Evolutionary tree for the sixth fragment in the coalescent simulation. The leaf node labels are as in Figure 1. This tree was used as the left-side tree in the repetitive phylogenetic simulation experiments. The right-side trees corresponding to the different recombination types were obtained as follows: 1) (recent recombination with a distant relative) strain 27 was moved to the same branch with strain 19. 2) (recent recombination with a close relative) strain 27 was moved to the same branch with strain 7. 3) (old recombination) the branch containing all the cyan strains was moved to become a part of the branch containing the red strains. 4) (recombination involving a miniature cluster) strain 11 was moved to the same branch with strain 19. 5) (recombined fragment obtained from outside of the populations in data) strain 27 was moved to its own branch, which joins the rest of the tree close to the root.

The used simulation setup was inspired by [31]. Each of the five different scenarios was generated by dividing the recombined gene into two parts, such that the left part was 500 and the right part 200 bases long. For both parts we used fixed evolutionary trees. For the left-side tree we used a tree corresponding to the sixth fragment in the coalescent simulation in the previous subsection. This tree is shown in Figure 5, and it was chosen as the basis for the repetitive simulations given the perfect correspondence between the tree and the estimated clustering (see Figure 5). The right-side trees were created by manually modifying the left-side tree in the different situations as follows: 1) a strain was moved to a branch on the other side of the tree, 2) a strain was moved to a nearby branch, 3) a branch containing all the members of a population was joined with another branch, 4) a strain from a miniature cluster was moved to another branch in the tree, and 5) a strain was moved to its own branch which joins the tree near the root. For details of which specific strains were moved, see caption of Figure 5. Figures of the right-side trees are shown in the supplementary material (Additional file 1). For each type of data, 200 replicate data sets were generated using software Seq-gen [32] using HKY model [33] with base frequencies arbitrarily specified as 0.1, 0.2, 0.3 and 0.4 for A, C, G and T respectively, and transition/transversion ratio set to 2.0. The three different levels of molecular variation were achieved by specifying three different heights of the tree: 0.1, 0.3 and 0.6, where the height corresponds to the distance from the root to any one of the tips in units of mean number of subsitutions per site.

We analyzed the generated data sets using the fixed populations shown in Figure 5. However, as the level of genetic variation is expected to affect the resolution of the clustering obtained from BAPS, it is of interest to investigate how the clustering would be different, if it had been estimated with BAPS. For this purpose, we used BAPS to cluster five arbitrary data sets corresponding to the first data set type and each of the three levels of molecular variation. (As the majority of sites is generated according to the left-side tree which is invariant in the data sets, we do not expect there to be any significant differences between the different types of data sets in this respect.) Indeed, the resolution increased with respect to the increasing height of the tree, because the divergence between different populations increased. When the height of the tree was 0.6, four out of five data sets were clustered in exactly the same way as the specified clustering, and the clustering for the remaining data set contained one additional cluster, corresponding to the branch containing strains 4 and 6. When the height was 0.3, all the estimated clusterings were exactly the same as the specified clustering, except that the miniature cluster with yellow label was merged with the cluster with red label. When the height was 0.1, both the miniature clusters (green, yellow) were merged with their closest clusters. Additionally, in one data set also clusters with blue and magenta labels were merged. Thus, it is possible that some of the investigated situations would not be encountered in practice, if the clustering was performed with BAPS (most notably, the recombination event involving a miniature population with tree height 0.1 would be undetected, because it is unlikely that the miniature cluster (green) would be identified as a separate cluster in BAPS analysis).

**Figure 6:**
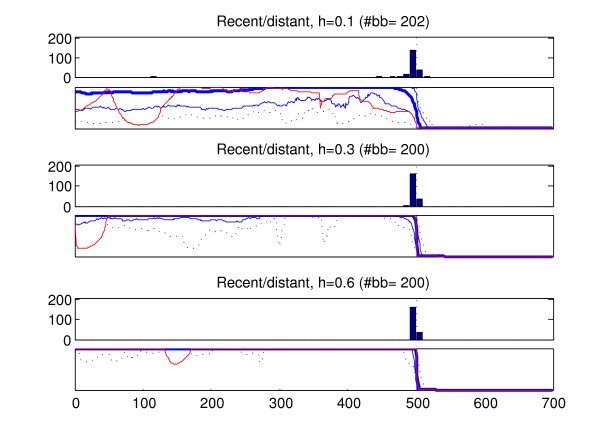
**Results, recent recombination event with a distant relative**. Summary of results for 200 strains which have undergone recent recombination with strains to which they are distantly related. Sites 501–700 have been obtained through recombination. The results are shown for three different heights of evolutionary trees (0.1, 0.3, 0.6, measured as the expected number of substitutions from the root to a leaf in a tree). For each tree height, two plots are shown: the upper plot shows a histogram of the inferred breakpoint locations, the lower plot shows five percentiles for the probabilities of the left-side origin given by the inferred marginal probability profiles (dotted line: 0th and 100th percentile, thin solid blue line: 5th and 95th percentile, thick blue line: 50th percentile). The solid red line shows one particular marginal probability profile. This profile is the one deviating most (in mean squared error sense) from the 'correct' profile, which in this particular case would assign probability unity for the left-side origin in sites 1–500 and probability 0 in sites 501–700. The total numbers of inferred breakpoints for different tree heights are shown in the titles of the respective plots (#bb). The x-axis coordinates shown below the lowest plot specify the location along the gene in any of the plots.

**Figure 7:**
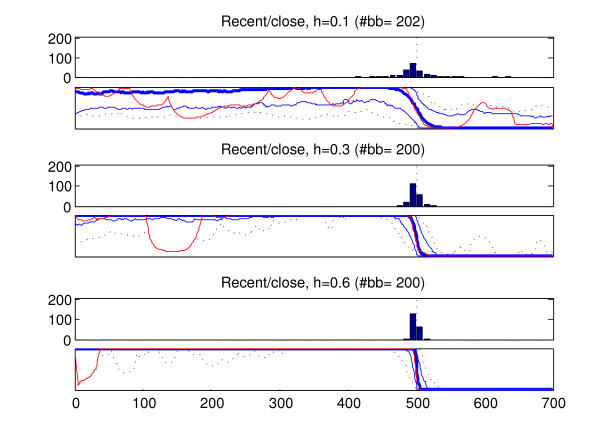
**Results, recent recombination event with a close relative**. Summary of results for 200 strains which have undergone recent recombination with strains to which they are closely related. Sites 501–700 have been obtained through recombination. See Figure 6 for the interpretation of the figure. As in Figure 6, the 'correct' marginal probability profile would assign probability unity for the left-side origin in sites 1–500 and probability 0 in sites 501–700.

**Figure 8:**
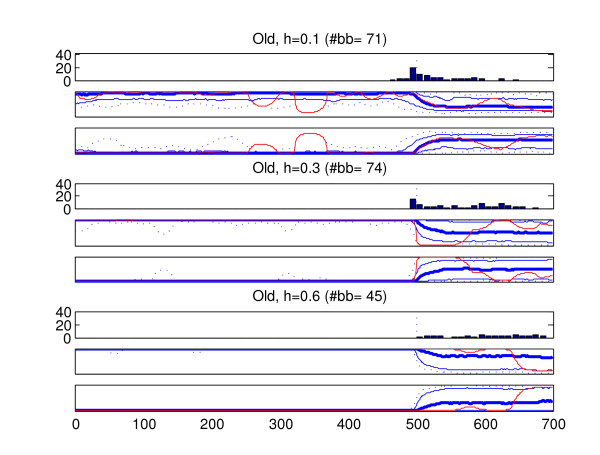
**Results, old recombination event affecting all strains in a population**. Summary of results for 200 strains belonging to a population whose all members have a recombined fragment from another population. Sites 501–700 have been obtained through recombination. See Figure 6 for the interpretation of the figure. Because the two populations now share the same fragment, the sites 501–700 could be assigned to either one of the possible populations. For this reason, also the probabilities assigned to the other possible right-side origin are shown (the third plot for each tree height), similarly to the probabilities assigned to the left-side origin (the second plot). In this case the 'correct' marginal probability profile would assign probability unity for the left-side origin in sites 1–500 and probability 0.5 in sites 501–700. The alternative right-side origin would be assigned in the 'correct' marginal probability profile probability zero in sites 1–500, and also probability 0.5 in sites 501–700. Similarly, the 'correct' optimal model profile would assign the sites 501–700 to either of the two populations. Notice that the y-scale of the histogram plots is different from other result Figures (6, 7, 9 and 10).

**Figure 9:**
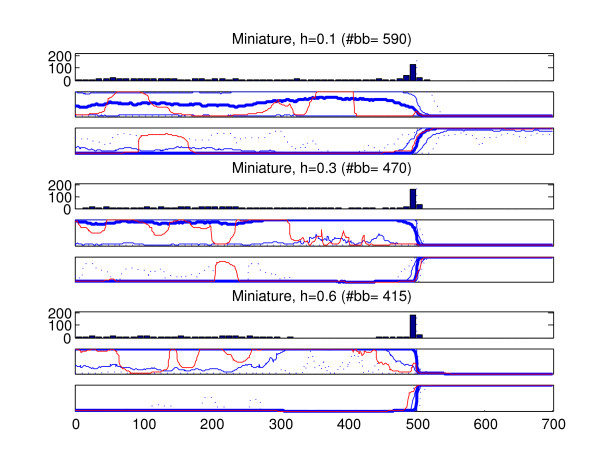
**Results, recombination event involving a miniature cluster**. Summary of results for 200 strains belonging to a miniature population with a recombined fragment from another population. Sites 501–700 have been obtained through recombination. See Figure 6 for the interpretation of the figure. As in Figure 8, the situation between the right- and left-side origins is asymmetric. Therefore, we show also a summary plot of the probabilities for the right-side origin in the marginal probability profiles (the third plot for each tree height). As in Figure 6, the 'correct' marginal probability profile would assign probability unity for the left-side origin in sites 1–500 and probability zero in sites 501–700. The 'correct' probabilities for the right-side origin would be zero in sites 1–500 and unity in sites 501–700.

**Figure 10:**
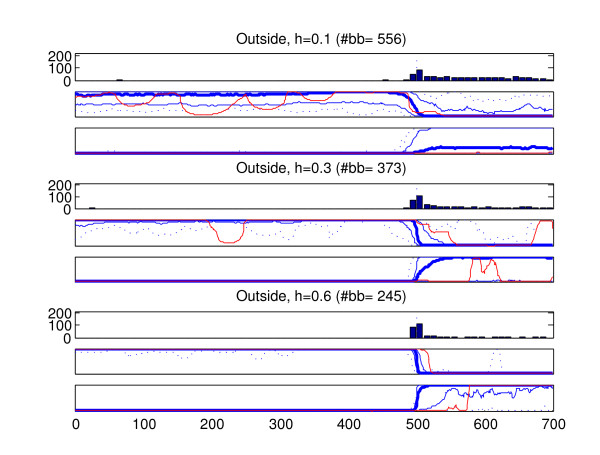
**Results, recombination such that the fragment is obtained from outside of the populations present in data**. Summary of results for 200 strains with a recombined fragment obtained from a population from which no strains are present in data. Sites 501–700 have been obtained through recombination. The figure is interpreted similarly to Figure 9. The 'correct' marginal probability profile would in this case assign probability unity for the left-side origin in sites 1–500 and probability zero in sites 501–700. The 'correct' probabilities for the right-side origin (outside population) would be zero in sites 1–500 and unity in sites 501–700.

We analyzed the recombined strains in the generated data sets with the introduced method (BRAT). In the data type in which more than one recombined strain was present in a data set (old recombination affecting all members of a population), only one of the recombined strains was analyzed from each data set. The results of these analyses are summarized in Figures 6, 7, 8, 9, 10. Here we describe the findings for each data type separately.

1. Figure 6 shows the results for the strains which have had recent recombination with strains to which they are distantly related. The results show that the method has little difficulty in identifying this type of recombination events. In the optimal model profiles the breakpoints are inferred very close to the correct location, and only in the situation where the populations are least diverged (h = 0.1) two additional breakpoints are present in the optimal profiles. The marginal probability profiles correctly assign high probabilities to the left-side origin in sites 1–500. (Only the left-side origin probabilities are shown, because the situation is essentially symmetric between the right-side and left-side origins.) Although the variation in the marginal probability profiles increases with decreasing divergence between populations, even in the least diverged case (h = 0.1) the worst-case profile assigns very high probabilities for left-side origin in a vast majority of sites 1–500, thus facilitating the correct interpretation.

2. Figure 7 shows the results for the strains which have had recent recombination with strains belonging to a nearby branch in the evolutionary tree. Because the populations of origin of the fragments are now closer to each other, some additional variation is added to the results as compared to those in Figure 6. Especially the locations of the breakpoints are now slightly more widely spread around the correct location and the left-side origin may be assigned higher values also in the sites 501–700. This is observed especially with h = 0.1. Yet, it can be concluded that the results still reflect very well the underlying biological truth.

3. Figure 8 shows the results for recombined strains which belong to a population where all members have a recombined fragment (old recombination). In this case, it would be equally correct for the optimal model to assign the right-side fragment to either of the possible populations. The presence of two alternative origins for the fragments clearly affects the inferred breakpoints in the optimal profiles. The number of breakpoints is around 70 for h = 0.1 and h = 0.3, indicating that at most about a third of the strains had any breakpoints in their optimal model profile while a majority of optimal models constituted of just one segment assigned to the left-side origin. The number of breakpoints is yet lower with h = 0.6. Furthermore, although these breakpoints are most often found close to the 500th site, they are also found spread along the whole fragment.

To aid in making the correct interpretation, the marginal probability profiles should assign clearly non-zero probabilities to both of the possible origins in sites 501–700, regardless of the inferred optimal model (optimally, both populations would be assigned a probability 0.5 in these sites). Interestingly, it is now the case with the least variation (h = 0.1) in which the marginal probabilities are closest to the optimal behavior, as even the 0th and 100th percntiles are clearly separated from zero and unity for probabilities assigned to both the possible origins. The explanation is that the amount of divergence between the populations after the recombination event matters here considerably more than the divergence before it (both decrease with decreasing h). Indeed, because in the right-side tree the population with the recombined fragment still constitutes its own subbranch in the branch of the other population, with increasing h the difference between these populations becomes statistically relevant. This can be seen in the case with h = 0.6, where the left-side origin is on average assigned clearly higher probabilities than the right-side origin in sites 501–700.

As can be seen in the worst-case profile with h = 0.3 and h = 0.6, the condition that both origins should be assigned clearly non-zero probabilities in sites 501–700 to aid the making of the correct interpretation does not always hold. However, with h = 0.3 the 5th and 95th percentiles are clearly separated from zero and unity, indicating that for the majority of strains the correct interpretation can be reached. We investigated this further by counting the number of strains such that for either origin there are maximally 10 sites in which the origin is assigned a probability less than or equal to 0.05 (among sites 520–700, the first 19 sites of the fragment are not taken into account, because close to the breakpoint there can be expected to be more variation in the probabilities). Altogether 169 strains out of 200 satisfied this condition indicating that in the majority of cases the correct interpretation can be reached. For the case h = 0.6 the same figure was only 78, because the right-side origin was assigned clearly lower probabilities in general than the left-side origin. This fact simply highlights the point that the populations have already become so diverged that the model treats them as distinct. Although the correct interpretation can be made for some strains, for other strains only traces of the shared evolution are still visible in terms of elevated probabilities for the right-side origin at some parts of the recombined fragment.

4. Figure 9 shows the results for the strains belonging to a cluster with only a limited number of strains (miniature cluster). These results highlight the fact that when there is considerable uncertainty concerning the nucleotide frequencies in the cluster to which the strain under investigation is assigned, the results should be interpreted with caution. The optimal models contains a large number of breakpoints within the left-side fragment, and there is also a lot of variation in the marginal probability profiles within this fragment. With h = 0.1 the worst-case probabilities assigned to the left-side origin in sites 1–500 are completely misleading. With h = 0.3 the situation is somewhat improved while with h = 0.6 the results are already decent, such that in the majority of sites even in the worst-case profile the left-side origin is assigned high probabilities. However, as discussed above, it is unlikely that the h = 0.1 case would be encountered in practice, if the populations are inferred using BAPS, because the miniature cluster would most likely be merged with another cluster (in which case the profiles would be less noisy but, if the recombination occurred between the merged populations, it would of course remain completely undetected). Nonetheless, it may be completely possible to identify a fragment which has been obtained from another population with at least a moderate number of strains, as the results for the right-side fragments illustrate.

5. Figure 10 shows the results for the strains where the recombinant fragment has its origin outside the populations represented in the data. These results quite expectedly show that the detection of the recombination is in such scenario more difficult than in the case where the origins of the fragments are present in data. Nevertheless, the statistical power to detect such recombination events increases with increasing divergence between the populations (increasing h). While with h = 0.1 the recombination from outside may in the worst-case be completely undetected, on average the results are already satisfactory with h = 0.3. With h = 0.6 the recombined fragments from the outside can be inferred with good accuracy. It is worth noticing that within the fragments obtained from the outside of populations the optimal model may contain short intervals assigned to different origins. This is simply a consequence of the fact that the optimal model must assign all the sites to some origin, and certain parts of the fragment may by chance always resemble strains in some population. Thus, if the marginal probability profile contains areas where the outside origin is assigned elevated probabilities, the optimal models should again be interpreted with caution.

In addition to the above simulation experiments, in the supplementary material we present experiments based on fairly simplistic stochastic forward simulations, which were initially utilized to investigate the elementary behavior of the developed method under various circumstances. Three main types of such experiments were performed: 1) balanced sample sizes and true sources of recombinant segments present in the data, 2) balanced sample sizes present but the true source of recombinant segments remaining outside of the available data, 3) unbalanced sample sizes with multiple species represented by a very limited number of strains. Especially the third situation setup provides some relevant additional information to the simulations presented here. Specifically, when there are several strains in a data set which are sole representatives of their true populations, such strains may be combined into a single hybrid-like cluster in the BAPS analysis (subsequently termed as 'hybrid cluster'). Because the estimates of the nucleotide frequency parameters of such clusters contain considerable uncertainty, the recombination profiles may look noisy, as with the data type involving recombination with a miniature cluster. For further illustration of hybrid clusters, and guidelines for drawing correct interpretations in a situation involving them, see the supplementary material.

To summarize the simulations briefly, we conclude that the method works very well in situations where a sufficient number of strains from populations involved in the recombination event are present in the data. In a situation where some fragment is shared by two populations, the optimal model most often contains no breakpoints, but the marginal probability profiles assign elevated probabilities to the alternative origins. We have also investigated and discussed cases where the nucleotide frequencies of an origin for some fragment include a lot of uncertainty (e.g. miniature cluster, outside origin, hybrid cluster). In such situations the optimal model profile often contains several short segments assigned to various origins, and the marginal probability profiles fluctuate, but do not indicate strong evidence for any particular population. In these situations we recommend a conservative way of making interpretations, such that only the conclusions which are strongly supported by the marginal probability profile should be considered. Also, we recommend investigating all the genes of a strain when making interpretations concerning any particular gene, as information pointing consistently to the same direction can be useful (for example several recombined fragments in different genes from the same population). Also, investigating the results for all the strains in a cluster as a whole may be helpful if there is some structure within the cluster. In the next subsection, we show how to use these guidelines when investigating a real data set.

### Burkholderia data

To illustrate the presented method with a real data set, we use *Burkholderia *data introduced earlier in [34]. The data set consists of 120 strains from *Burkholderia cepacia *complex (Bcc). The Bcc is a widespread group of related bacteria found in a variety of environments, although little is known about the natural history of these organisms [35]. The Bcc are potentially economically very important but are also important opportunistic pathogens among vulnerable individuals [35].

The strains in the data set either belong to one of nine different species, or are unlabelled. Some of the species are further divided into subgroups. The names of the species, as well as the number of strains from each species are shown in Table 1. Each strain is characterized by seven genes, the lengths of which vary between 301 and 454 bases. Table 2 shows a distance matrix for the species present in the data.

**Table 1:** *Burkholderia *data.

Species	Label	Strains
*B. cepacia*	*I*	14
*B. multivorans*	*II*	13
*B. cenocepacia*	*IIIA*	10
*B. cenocepacia*	*IIIB*	10
*B. cenocepacia*	*IIIC*, *D*	3
*B. stabilis*	*IV*	6
*B. vietnamiensis*	*V*	14
*B. dolosa*	*VI*	3
*B. ambifaria*	*VII*	12
*B. anthina*	*VIII*	7
*B. pyrrocinia*	*IX*	5
*others*	*X*	23

The species present in the *Burkholderia *data set, their labels, and the number of strains from each species.

**Table 2:** The distance matrix for the species in the *Burkholderia *data set.

	*I*	*II*	*III*	*IV*	*V*	*VI*	*VII*	*VIII*	*IX*	*X*
*I*	39	175	106	145	160	199	143	144	169	125
*II*		26	169	189	179	149	174	186	206	183
*III*			70	139	165	193	130	139	165	124
*IV*				21	184	207	163	162	119	146
*V*					8	186	164	167	211	166
*VI*						12	186	197	215	199
*VII*							29	134	177	130
*VIII*								85	177	142
*IX*									97	141
*X*										119

The distances are measured as the number of differing bases in the sequences. The off-diagonal values are average distances between the members of the corresponding species. The values on the diagonal are average distances between the members of any single species. The total length of the seven genes in the data is 2773 bases.

The Bcc have recently emerged as human pathogens and provide us with a fascinating group of important model organisms with varied population biology across their different species. Human colonisation by some strains and their ability to survive anti-infectives may have been heightened by adaptation processes with other Bcc strains [35,36]. They represent an exceptional group to examine how opportunistic pathogens have evolved and how we might use this information to inform on their control.

The novel MLST (Multi Locus Sequence Typing) scheme of the Bcc having identical alleles and therefore comparable sequence data across >9 species, has provided us with a unique framework, enabling us to explore mutation and recombination events and their impact upon evolution, speciation, niche adaptation, antimicrobial resistance and pathogenicity. Although co-colonisation of different Bcc is documented clinically [37], little is really known about the ecology and genetic exchange of Bcc in the environment. From genome sequences available for several Bcc species it is clear that they have multi-chromosome genomes (8 Mb) of which 0.8–1.0 Mb can be accounted for by mobile elements, phage, IS and genomic islands. The distribution and functional impact that this laterally acquired DNA has on pathogenicity is largely unknown [38], though comparisons can be made with the B. pseudomallei and B. mallei genomes [39].

The results obtained by the introduced method will provide a starting point in answering the following biologically relevant questions:

1. Do genetic alterations facilitate niche jumping from different environments to clinical settings?

2. How does recombination affect pathogenicity and pathogen-host interactions and how does this shape our understanding of natural communities?

3. What are the roles of local or widespread recombination events in the emergence of new species?

The unsupervised mixture classification with BAPS identified eight clusters for the *Burkholderia *data, shown in Table 3. The obtained population structure is highly consistent with the species classification given in the database. Only one of the species, *B. cenocepacia*, is split into two distinct groups, corresponding to different subspecies for *B. cenocepacia*. In total, two of the obtained clusters contained strains from two or more species.

**Table 3:** The clustering of the *Burkholderia *data.

Cluster	Species included in the cluster
1	*B. cepacia*
2	*B. stabilis, B. pyrrocinia*
3	*B. ambifaria*
4	*B. cenocepacia (IIIC, IIID), B.anthina, others*
5	*B. multivorans*
6	*B. dolosa*
7	*B. cenocepacia (IIIA, IIIB)*
8	*B. vietnamiensis*

The eight clusters for the Burkholderia data set identified by the unsupervised mixture clustering analysis.

In addition to using BAPS to identify the required clustering for the data, we carried out an admixture analysis for the data with an admixture model, also implemented in BAPS [19]. As opposed to the methodology introduced here, BAPS admixture model considers simultaneously the observed pieces in the whole genome to estimate the appropriate weights for the admixture proportions corresponding to the different ancestral origins, while not reflecting the actual locations of the recombined areas in the sequences. For this reason, it is also possible that BAPS may fail to identify some recombination events, even in the presence of a strong signal within a relatively short interval of bases, when the signal is not significant on the level of the complete observed concatenated sequence. The results obtained by the introduced method are compared with the corresponding admixture results obtained by BAPS.

Here, our goal is not to give a complete description of the obtained results or to draw profound biological conclusions. Rather, we aim to illustrate the behavior of the introduced methodology (BRAT) in a realistic challenging setting. To do this, we consider in detail three different specific issues concerning the structure of the molecular data. Each of these issues is illustrated by estimated sequence structure of a particular strain and the related statistical characterization of the uncertainty, and is accompanied by a different biological interpretation. To maintain readability, we leave out some details of the analysis leading to presented conclusions. A more detailed description can be found in the supplementary material (Additional file 1).

#### *B. cenocepacia IIIC*

**Figure 11:**
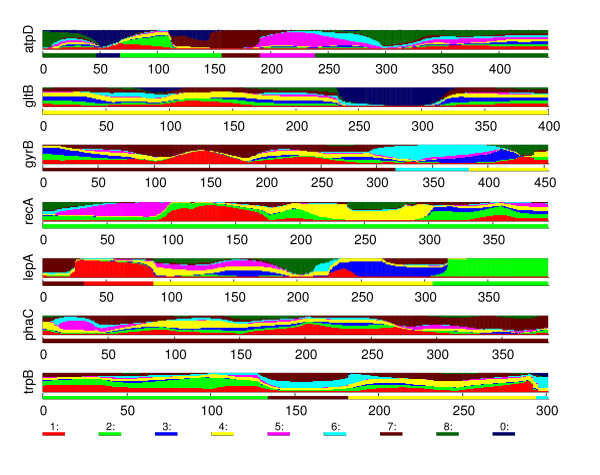
***B. cenocepacia IIIC***. The recombination profile of the strain belonging to *B. cenocepacia IIIC*, discussed in the text.

The BAPS admixture analysis identified five strains as having statistically significant evidence for admixture. In particular, both strains from species *B. cenocepacia IIIC *were identified. As our first example, we consider in detail one of the admixed *B. cenocepacia IIIC *strains. In total six of the clusters were associated with non-zero estimated admixture coefficients for this strain in the BAPS analysis, the estimates being: Cluster 1: 0.05, Cluster 2: 0.08, Cluster 4: 0.59, Cluster 5: 0.02, Cluster 6: 0.03, and Cluster 7: 0.23. The recombination profile obtained by the introduced method is shown in Figure 11. Immediately, areas in the sequence having a particular cluster as the most likely origin can be identified for each cluster associated with non-zero coefficients in the BAPS admixture analysis. Furthermore, some segments are also assigned to Cluster 8, and to the cluster corresponding to an unknown origin.

By examining the recombination profile and using the built-in option of BRAT to calculate the distances of the strain to the different clusters in a given interval, we were able to identify areas in the sequence with strong evidence of shared ancestry with the Clusters 7 and 2, corresponding to the second and the third largest coefficients in the BAPS admixture analysis. For example, the third and the sixth gene have long areas where the Cluster 7 dominates the marginal probabilities, while the rightmost 80 bases in the fifth gene are strongly associated with the Cluster 2. The other clusters, suggested by the optimal model profile as possible origins for some parts of the sequence were also investigated in a similar manner. Interestingly, we were not able to find areas with strong evidence for the other origins, not even for the Cluster 4, which had the largest coefficient in the BAPS analysis. While the distances (in mutations) were somewhat supporting the suggested origin, they were not conclusive, as with the Clusters 7 and 2.

To focus on the biological interpretation for this strain we recall that the strain was assigned in the unsupervised classification phase to a cluster (4), which consists of strains from species *B. cenocepacia IIIC*, *IIID*, *B. antina *and a group named as others. Furthermore, many of these species are represented by only a small number of strains. Thus, it is possible that the Cluster 4 is an example of what was earlier referred to as a 'hybrid cluster', i.e. a collection of strains from species which are represented in the data set by an insufficient number of strains to be identified as a distinct cluster. Such a misleading classification for the strain under investigation is also suggested by the fact that there are some areas in the sequence where the cluster corresponding to the unknown origin gets high probabilities.

Thus, we can summarize our findings for this strain as follows. There is strong evidence that some parts of the sequence share ancestry with the Cluster 7 (*B. cenocepacia IIIA *and *IIIB*) and some parts with the Cluster 2 (*B. stabilis *and *B. pyrrocinia*). Furthermore, there is strong evidence that there are areas in the sequence which are not closely related to either of the mentioned clusters. These areas are mostly represented by the Cluster 4, and some other clusters. However, there is no strong evidence that these clusters in fact represent the true origin for these areas, they may just be the statistically most appropriate ones of the available alternatives.

#### *B. cenocepacia IIIA *and *IIIB*

**Figure 12:**
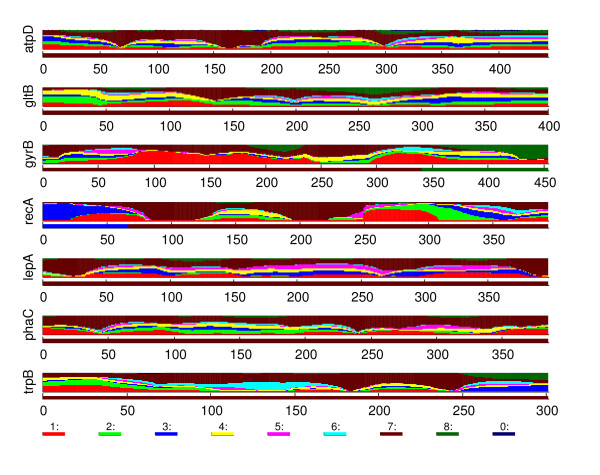
***B. cenocepacia IIIB***. The recombination profile of the strain belonging to *B. cenocepacia IIIB*, discussed in the text.

In the mixture clustering, the subspecies *IIIA *and *IIIB *of *B*. *cenocepacia *were clustered together, which reflects their close evolutionary relation. In the BAPS admixture analysis none of these strains was identified as having admixture with species in the other clusters. However, the recombination profiles calculated for the strains support a different conclusion. There is also a clear distinction in the recombination profiles between the strains in the two groups. Out of the ten *IIIA *strains, only two had one recombination event in one gene in their optimal recombination model. On the other hand, all the ten *IIIB *strains had at least one recombinant segment in their optimal model profiles, and most of these strains had actually a couple of such segments. These segments were mostly assigned to the clusters corresponding to species *B. ambifaria *and *B. vietnamiensis*. Figure 12 shows the profile of one strain belonging to *IIIB*. The segment assigned to *B. ambifaria *can be seen at the beginning of the fourth gene (blue color). The segment assigned to the *B. vietnamiensis *species can be seen at the end of the third gene. By investigating the marginal probability profile for these areas, and calculating the distances to the different clusters, we can conclude that these intervals have their origins in a different source than the rest of the sequence. Analogous conclusions can be made for other suggested recombinant segments from *B. ambifaria *and *B. vietnamiensis *for the strains of species *B. cenocepacia IIIB*. On the other hand, the two possible recombinant segments which were observed in the optimal profiles of *B. cenocepacia IIIA *did not obtain conclusive support. Thus, the biological interpretation of these results would be that the difference between *B. cenocepacia IIIA *and *B*. *cenocepacia IIIB *is that *IIIB *has had recombination with *B. ambifaria *and/or B. *vietnamiensis*, while *IIIA *has not.

#### *B. pyrrocinia*

**Figure 13:**
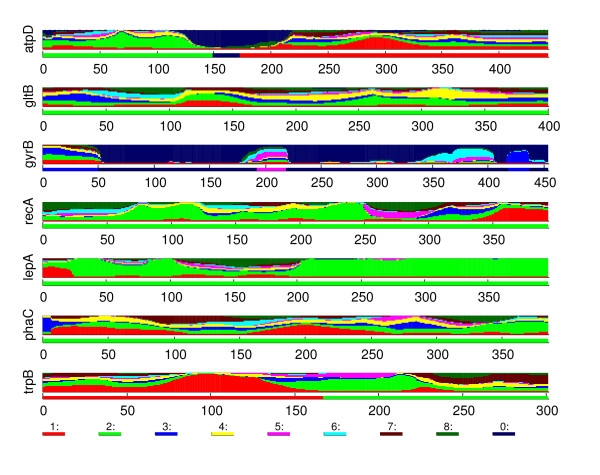
***B. pyrrocinia***. The recombination profile of the strain belonging to *B. pyrrocinia*, with the third gene assigned to an unknown origin.

The recombination profile of a strain belonging to *B. pyrrocinia *species is shown in Figure 13. This strain was not identified as admixed in the BAPS admixture analysis. However, the profile shows a striking feature which is not present in any other profile in the whole data set. Apart from some small stretches, the third gene is assigned as a whole to an unknown origin in the optimal model, i.e. origin not represented by any of the known clusters. This can also be seen, when the average distances to the different clusters are calculated. The average distance in this gene to the cluster closest to the strain is 81 mutations, while the average distance in this gene between the strains in any cluster is at most approximately 28 mutations. Thus, this gene is dozens of mutations further apart from any species present in the data. Because this is the only gene of the strain with this characteristic, a plausible biological explanation would be that this gene is obtained as a whole by recombination with some species not present in the data set.

## Discussion

We have introduced a Bayesian approach for the identification of recombination events in DNA sequences obtained from bacteria. Uncertainty related to a solution can in our approach be expressed in terms of marginal posterior probabilities for origins of different sites in the sequences. The computational advantages of our approach stem from a characterization of the putative origins for a segment by parameters corresponding to relative frequencies of different nucleotides at each site. In this respect our approach is less realistic than the recently introduced Bayesian approaches based on phylogenies, which have an explicit model for mutations (e.g. [7,13]). In our model the variation caused by mutations in bacterial populations is taken into account by the varying nucleotide frequency parameters. Also, as opposed to the phylogeny-based approach, we do not try to model the complete evolutionary histories of all putative recombinant segments, but determine a probabilistic characterization of plausible origins for each detected segment. If there are several plausible origins for some segment, the corresponding posterior probabilities will be substantial for all of them. At the same time, this provides indirect evidence of a common evolutionary history of the possible origins in this segment. The utilized approximations involve two open parameters *L *and *L*_max _which have a smoothing effect on the calculated marginal probability profiles. These parameters are given default values, which are deemed reasonable through theoretical considerations. Also, the behavior of the method under the default selections has been examined through substantial simulation experiments, and the effect of these parameters has been illustrated through examples in the supplementary material (Additional file 1). However, we wish to point out that no in depth experimental study has been carried out to assess their effect, and possible bias caused to the calculated marginal probability distributions, in all possible situations.

The adopted simplifications in the model formulation allow us to apply the method efficiently to data sets consisting of hundreds of strains. For example, an analysis of all the seven genes of one *Burkholderia *strain, discussed in real data analysis, takes about 85 seconds on a desktop PC with a 2.2 GHz processor. As the strains are analyzed independently, the required CPU time is linear to the number of strains and genes to be analyzed.

We utilized BAPS software to define the reference populations for our analysis. It would also be possible to use a reference clustering obtained by some other method, although we have not considered such an alternative in this work. There are two primary reasons for this. Firstly, the stochastic urn model for unsupervised classification, combined with the stochastic optimization algorithm, as implemented in BAPS, has a superior accuracy compared to standard Bayesian computation, in particular, when the data represents a complex population structure with many underlying clusters [27]. Secondly, due to the similarities in the likelihood function, by utilizing a preclustering obtained with BAPS one will never in practice encounter a paradoxical situation in which some strain is assigned to a cluster for which the recombination analysis does not yield high probabilities anywhere within the observed sequence. As illustrated by several examples in this article and the supplementary material, BAPS is able to provide a clustering which in general reflects very well the evolutionary relationships of the strains. The only scenario in which a clustering obtained by BAPS clearly deviated from the 'true' population stratification occurred when some populations were represented by a very limited number of strains in the data set. Even then, the behavior of BAPS is well-characterized, leading for example to a hybrid cluster, as discussed in illustrations in the previous section, as well as in the supplementary material.

The reference populations were summarized in our approach by posterior distributions for the nucleotide frequencies. An alternative would be to use a consensus sequence to summarize the reference populations, enabling one to proceed for example with the phylogenetic framework. However, such a strategy would be inferior as it neglects the variation within the sequences in a population. For example, a cluster may in reality contain two subgroups of strains, which are otherwise similar, but different in some segment. Then, a strain which reminds either of these subgroups in this segment will show elevated posterior probabilities for the corresponding cluster in our approach (see e.g Figure 1d in the supplementary material), while the consensus strain would be likely to abandon the information concerning one of the subgroups. Also, taking the variation into account is important when considering the possibility of some segment originating from an outgroup, i.e. a population not represented by any of the strains in the data set. For instance, assume that a particular strain *i *has an elevated level of distance to the nearest cluster (population) within some gene segment. Further, if the strains in that cluster are identical or nearly identical over the same gene segment, and the remaining clusters are also internally homogeneous within the segment, then it is likely that this segment of the strain *i *has a deviating ancestry, and the outgroup will be associated with a high posterior probability. On the other hand, if there is more variation in the observed nucleotides within the nearest cluster, especially at those sites at which the strain is most different from the strains in the cluster, then it would be more likely that the particular segment is associated with the cluster, and consequently, the outgroup would not be assigned high posterior probability in our approach. See also a related discussion in [40].

The optimal use of the introduced method requires that the estimates of the relative frequencies of different nucleotides in the populations must be reasonably good. In practice this means that each underlying population must be represented by a sufficient number of strains in the data set. What sufficient means depends in practice on characteristics of the data, for example on the level of divergence between and within different clusters. In our analyses we have discovered that the calculated posterior probabilities for strains in populations represented by one or two strains may look noisy. On the other hand, already populations with five strains seem to provide a sufficient level of accuracy. Fortunately, it is usually quite straightforward to recognize the situations in which the interpretation of the results requires extra care, e.g. when the cluster to which the strain belongs is very small, or constitutes a so-called hybrid cluster. Notice also that while the introduced method works best with a sufficient number of strains present in a data set, the phylogeny-based methods are mainly intended to be used with a limited number of strains only. Thus, in addition to the different levels of model abstraction, as discussed above, the two methods also differ by the type of a data set for which they are the most suitable. For these reasons, we do not consider the two approaches as exclusive, but rather as complementary to each other.

Apart from the sizes of the reference populations and the quality of the clustering solution, the reference samples may be distorted by strains which are recombined themselves. This may be expected to increase the level of noise in the resulting profiles. However, as long as the reference populations fairly adequately correspond to the different types of strains present in a data set, we do not expect this to actually lead to wrong interpretations, but just increase the uncertainty related to the conclusions. The coherence of the reference populations is ensured by using BAPS for clustering the strains. For example, assume that there are some 'mosaic-like' strains in a cluster, say *k*, carrying a recombined segment whose origin is different compared to those strains which are the 'pure' representatives of the cluster. Consider then recombination inference for a strain which resembles closely the 'mosaic-like' strains in this particular segment. The posterior probabilities may then be clearly elevated for the origin corresponding to the cluster *k*, if there is no other cluster representing solely the 'mosaic-like' strains. However, because the reference clusters are defined only in terms of the strains they contain, in reality there are no 'pure' or 'mosaic-like' representatives of a cluster. Consequently, the fact that such a cluster is associated with elevated probabilities highlights the point that the cluster hosts strains which might share ancestry with the strain under consideration in this particular gene segment. Thus, the uncertainty illustrated in the profiles is increased in the manner one would expect.

We end the discussion by considering briefly the process of using the introduced method to analyze a data set. The task of statistically detecting recombination among the observed strains is a challenging one, due to the many possible uncertain elements involved. For this reason, the presented method, or any other statistical method for the same purpose, should not be used as a black-box tool, and the results inevitably require some subjective interpretation. To reach the correct conclusion, it is important to understand the behavior of the method in any specific situation. For this reason, we strongly encourage all potential appliers to read carefully through the examples in this paper. As a general strategy, we suggest that the first step of the analysis is the investigation of the optimal model profile. If no recombinant fragments are present in the optimal model profile, then it is plausible to conclude that no recombination has taken place. If the optimal model contains recombinant fragments, then all other available information should be taken into account when making the interpretation, including the calculated marginal probability distributions for the origins of the sites, the molecular distances to different clusters, the levels of molecular variation within the clusters (the last two are immediately available from our method for any selected segment) and biological knowledge about the species under investigation. Optimally, the putative recombination events would be confirmed by independent *in vitro *experiments, as e.g. in [41].

## Conclusion

Statistical discovery of recombinant and other 'anomalous' segments within DNA sequences has been an area of considerable research activity for more than two decades. In general, it has been shown in a multitude of contexts that model-based approaches provide quite accurate characterizations of the evolutionary processes, and help to assess uncertainty related to the central issues of interest. However, regarding the analysis of large bacterial DNA databases, the currently available model-based tools have restricted applicability in practice. The methodology introduced here is precisely intended to bridge this gap between the complexity of the currently existing data and the capacity of the methods. The importance of this issue will further increase in the future, as a consequence of the evolving sequencing techniques which will enable the investigation of much larger quantities of samples, as well as a denser coverage of the genomes.

Our statistical tool (BRAT) is developed to provide the results of the Bayesian analysis in an easily utilizable format, including high-resolution graphical displays of the estimated structures of the investigated sequences.

## Authors' contributions

JC and PM developed the Bayesian model. PM implemented the method and carried out the analyses. AB and WPH contributed in the biological aspects of the model. AB, CD, and EM provided the example real data set and expert knowledge about *Burkholderia*. AB, JC, WPH and PM wrote the article. (The lists are in alphabetical order.)

## Supplementary Material

Additional File 1 **Package of supplementary material**. File *BRAT_supplementary.zip *available at  includes a file BRAT_supplementary_text.pdf, which contains additional information on following issues: 1) elementary simulation experiments, 2) illustration of the effect of used parameter values and utilized approximations, and 3) a description of the real data analysis with more details than presented in the main text. The zip package also includes two folders. The folder "Coalescent_results" contains complete results for the analyzed coalescent data set, see the README file in the folder for further details. The folder "Trees for repetitive simulations" contains the figures of the left-side and right-side trees used in different types of simulations. Click here for file
