# Supplementary material for: Bayesian modeling of recombination events in bacterial populations
Source: BMC Bioinformatics. 2008 Oct 7;9:421. doi: 10.1186/1471-2105-9-421 (PMC2579306; doi:10.1186/1471-2105-9-421)
Supplement: Additional File 1 — Package of supplementary material. File BRAT_supplementary.zip available at includes a file BRAT_supplementary_text.pdf, which contains additional information on following issues: 1) elementary simulation experiments, 2) illustration of the effect of used parameter values and utilized approximations, and 3) a description of the real data analysis with more details than presented in the main text. The zip package also includes two folders. The folder "Coalescent_results" contains complete results for the analyzed coalescent data set, see the README file in the folder for further details. The folder "Trees for repetitive simulations" contains the figures of the left-side and right-side trees used in different types of simulations. [file 1471-2105-9-421-S1.zip › BRAT_supplementary_2/brat_supplementary_text_2.pdf]

Contents

1 Elementary simulation experiments 1
1.1 Simulated data sets with recombination events between populations 1
1.2 Simulated data sets with recombination events from hidden populations 4
1.3 Simulated data sets with complex population structure 4
1.4 Summary of the elementary simulations 8

2 Illustration of e¤ects of parameter values and used approximations 8
2.1 E¤ect of parameters L and L_{max} 8
2.2 E¤ect of approximation for intervals outside the segment 10

3 Detailed description of real data analysis 11

1 Elementary simulation experiments

As a basis for the simulations, we imitate the features present in a real dataset consisting of sequences of seven housekeeping genes for a set of *Burkholderia* strains (for details, see the section of *Burkholderia data* in the main text). More specifically, we use here the same number (7) and the same lengths of the genes. Also, we use the di¤erent *Burkholderia* species available to specify realistic levels of molecular variation within and between populations. In the *Burkholderia* data the average number of sites with di¤erences in the bases between two identified subpopulations varies roughly within the interval 100-200 bases, among the total of 2773 sites. The average number di¤erences within a pair of strains from the same population varies approximately between 10-100 bases.

The following scheme is used for simulating the data sets. First, a base sequence of 2773 nucleotides is randomly simulated. From this base sequence, a sample of population kernel sequences is generated, by mutating independently each nucleotide of the kernel sequence with the probability 0.035 (´ p_1). Hence, the sampled population kernel sequences di¤er on average by slightly less than 200 bases. The actually used sample strains for each population are finally obtained from the corresponding population kernel sequence by randomly mutating each nucleotide with the probability 0.005 (´ p_2). Thus, the average distance between any two strains in the same population is approximately 28 mutations.

1.1 Simulated data sets with recombination events between populations

The following types of simulated data sets containing recombination events were generated.

1. A data set of 3 populations (red, green, blue), each of the size of 15 strains. In addition, there were two strains from the green population, such that one of the strains had a recombined sequence fragment from the blue, and the other from the red population. The recombinant segments were of length 100 bases and their locations were chosen randomly.

2. A data set of 3 populations (red, green, blue), each of the size of 15 strains. In addition, there were 15 strains from the red population, such that each had a randomly located recombined sequence fragment of a random length of 50-100 bases from the green population. Thus, the data set included 60 strains in total.

3. A data set of 3 populations (red, green, blue), each of the size of 15 strains. In addition, there were 15 strains simulated from the green population, such that they all had a recombined sequence fragment of length 150 bases from the blue population in the same location in their genomes. Thus, the data set included 60 strains in total.

From the simulated data sets 1 and 2, we show here results for one randomly chosen strain and gene containing a recombination event. From data set 3 we show one randomly chosen strain and gene with recombination and, for comparison, a gene of 'pure' ancestry. The results are all shown in Figure 1. The results in Figure 1 are interpreted as follows. For each gene, two plots are shown, a narrow plot below a wider plot. The di¤erent colors in the plots correspond to the clusters obtained in the unsupervised mixture clustering analysis. The narrow plot shows the optimal model (ρ, Z) for the gene, i.e. the assignment of each base in the sequence to a cluster according to the optimal model. The wider plot shows graphically the marginal probability distribution for X_j, $j = 1, \ldots, n_g$, the origin of the jth base in the gene.

Figure 1 reveals that the described model is able to identify areas of recombination with good accuracy in all these cases. Also, the estimated probabilities provide a clear support for the correct interpretation concerning the recombination events. In the unsupervised clustering analysis using BAPS that was performed before the recombination modeling, the correct three distinct subpopulations were always identified. Furthermore, the recombined strains were always assigned to the ancestral group from which most of that strain's genome had originated.

It is interesting to investigate the strain of pure ancestry in Figure 1d. For this strain, the area 185-335 is clearly assigned non-zero probabilities for the green population (and also for the red one). This is due to the fact that there is a group of 15 'green' strains (out of 30 green strains in total) having a recombined 'blue' fragment in this genomic area. Therefore, in this area, the 'blue' strain resembles strains assigned to the green population. Notice that some fluctuation in the marginal probabilities can be seen all over the genes, and also there is a boundary e¤ect, which a¤ects the probabilities close to the endpoints of some of the recombined fragments.

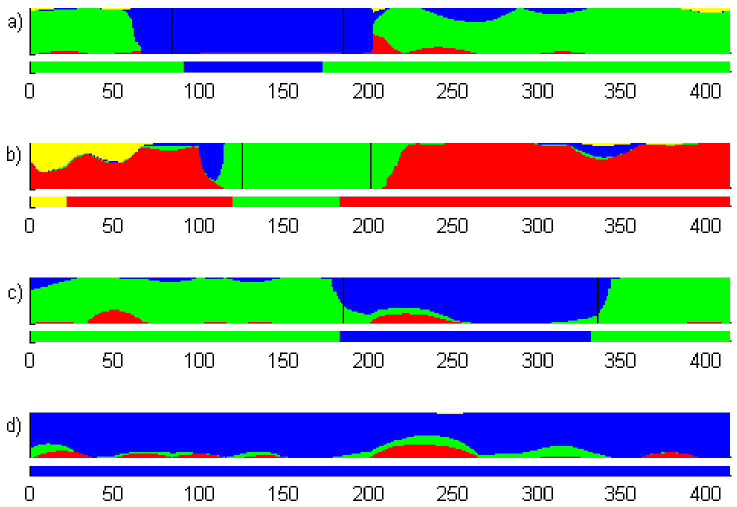

Figure 1: Results from the analyses of simulated data with recombination events between populations. a) green strain, with sites 85-184 from blue population (data set 1). b) red strain with sites 126-201 from green population (data set 2). c) green strain with sites 185-335 from blue population (data set 3). d) blue strain (data set 3). The recombination breakpoints are marked by vertical black lines in all the figures.

1.2 Simulated data sets with recombination events from hidden populations

In the previous examples, the source populations for recombined fragments were present in data. Next we consider situations where this is not true. To achieve such ancestral schemes, we generated the following data sets.

4. Firstly, four populations were simulated using the same parameter values as in the previous examples (p_1 = 0.035, p_2 = 0.005). Secondly, 15 strains from three of the populations were chosen to compose the data set. Finally, one strain from the first population was chosen to have a recombined fragment in the first gene at sites 85-185 from the remaining fourth population, which is not present in the data.

5. An identical setup to scheme 4, except that the average divergence between the members of a population was made smaller (p_1 = 0.035, p_2 = 0.0015).

6. An identical setup to scheme 4, except that the average divergence between any two populations was made larger (p_1 = 0.07, p_2 = 0.005).

The unsupervised clustering phase utilizing BAPS found for all these data sets the correct three underlying populations. Figure 2 shows the results of the recombination analysis for the recombined strains and genes in simulated data sets 4-6. A comparison with results in Figure 1 reveals that the estimation of recombination is here clearly harder, when the population corresponding to the origin of the recombined fragment is not present in the data. Still, in all these cases our method is able to identify some gene part correctly as emanating from an outside source. In 2a, parts of the recombined segment get incorrectly high probabilities towards the blue or red populations. Reducing the divergence within populations provides some improvement, however, considerable uncertainty still remains about the correct origin (Figure 2b). Increasing the divergence between the populations increases statistical power and allows for detection of anomalous areas in the genome with high certainty, as can be seen from the result in Figure 2c.

1.3 Simulated data sets with complex population structure

Realistic data sets often include strains from populations from which only a very limited number, say one or two, strains are available. Statistical analyses of such data could easily lead to misleading conclusions, if the results are interpreted carelessly. As a minimum requirement, the used methodology should be able to highlight the uncertainty related to the results concerned with such strains. Here we investigate the behaviour of our modeling approach, when some of the populations are underrepresented in the data. Strains belonging to populations of sizes one or two, will here be called as outliers and such populations will be termed as miniature populations. As illustrations, we use the following simulated data sets.

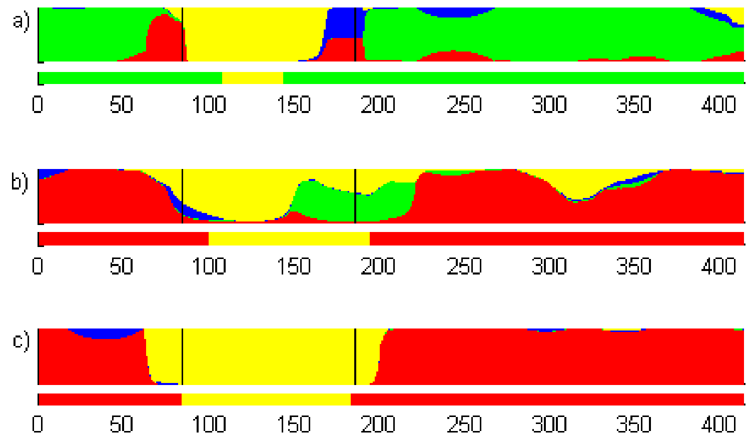

Figure 2: Results from the analyses of simulated data with recombination from outside of given populations. In all the figures, the section 85-185 in the shown gene was obtained from an unknown source. a) green strain from data set 4. b) and c) red strains from data sets 5 and 6. The unknown origin is represented by the yellow color in all the figures.

7. Four populations were simulated, exactly as before (p_1 = 0.035, p_2 = 0.005), however, three of which had 15 strains and the fourth only one strain.

8. Three populations with 15 strains were simulated. In addition, five populations of two strains and five populations of only one strain were included in the data set. All the populations were simulated using the same parameters values (p_1 = 0.035, p_2 = 0.005) as before.

9. The same population setup as in 8 was used, but with parameter values: p_1 = 0.035, p_2 = 0.0015. Thus, the average distance between the strains of any population is decreased.

10. Similar populations as in 9 were generated, but one outlier strain was given a recombined sequence fragment (sites 85-235 in the first gene) from a population containing fifteen strains.

Notice that the data sets 7-9 consist only of 'pure' strains with no recombination events at all, and that the data set 10 has one strain with recombination.

Figure 3 shows recombination results for data sets 7-10. In general, there seem to be four di¤erent types of outcomes from the unsupervised classification step with BAPS, when miniature populations are present in the data. The different alternatives are as follows: 1) miniature population is correctly identified

as one cluster, 2) miniature population is joined with the closest big population, 3) many miniature populations join together into a single cluster (such a cluster will be termed here as 'hybrid cluster'), and 4) many miniature populations are joined together into a single cluster, which is further joined with the closest big population. The actual outcome depends on the level of divergence between and within the populations. If only one miniature population is present in data, then either 1) or 2) may occur, and if there are many miniature populations in the data, then 3) or 4) are the most likely outcomes.

For the data set 7, the clustering phase yielded the correct four populations. Figure 3a shows the result for one gene of the strain in the singleton cluster (green) in data set 7. As can be seen from Figure 3a, there is considerable uncertainty related to the origin of the strain, and the most likely origin varies strongly along the gene. Also, there are areas where the 'outside' origin (magenta) is assigned high probabilities. Similar features were observable in all the genes of the strain. Notice also that, excluding the strain under investigation, there are no samples from green population. This is visible in Figure 3a, such that the 'outside' origin and the green population get equal probabilities at every location, because equally little is known about them.

For the data set 8, the unsupervised clustering found correctly the strains in the three big populations. The strains in the miniature populations formed a hybrid cluster, which was further combined with the green population. Figure 3b shows the profile for a true green strain and 3c shows the profile (one gene) of an outlier assigned to the green cluster. In Figure 3b, as in other genes of that strain, the evidence is conclusive for the correct origin. On the contrary, Figure 3c shows a short area, where the 'outside' origin (yellow in Figures 3b and 3c) is assigned high probabilities. Such areas were also found in other genes of the strain. Thus, instead of representing a true recombination event from an outside source, these areas are here an indication that neither the population to which the strain was assigned, nor any other population, is a proper origin for the strain as a whole. Notice also that the green population is assigned in general smaller probabilities in Figure 3c than in Figure 3b.

For the 9th simulated data set, the unsupervised clustering phase identified correctly the big populations. As opposed to the 8th data set, the miniature populations formed now a hybrid cluster (blue), distinct from the rest of the clusters. Figure 3d shows the profile of one strain and gene from that population. Again, similar features as in Figure 3c are present, i.e. the population to which the strain was assigned (blue), does not get very high probabilities anywhere, and several small areas where the outside origin (magenta in Figure 3d) is assigned high probabilities, can be seen in all the genes. However, even if a dubious clustering of an outlier strain may lead to an ambiguous recombination profile, the identification of some recombined segment from a known source population may still be possible. An example is shown in Figure 3e, where an outlier strain assigned to the green hybrid cluster has in the shown gene the fragment of sites 85-235 coming from the red population.

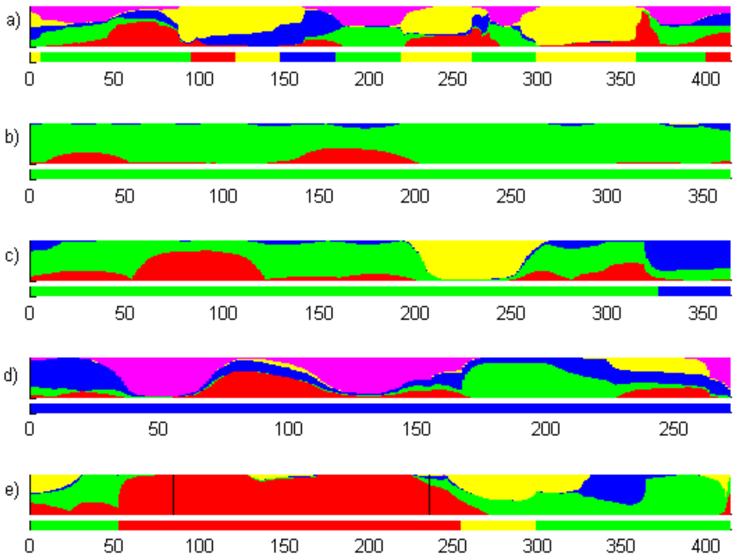

Figure 3: Results from the analyses of simulated data with complex population structure. a) A gene from the strain belonging to the singleton population (green) (data set 7). The unknown origin is represented by magenta. b) and c) Genes from two di¤erent strains belonging to the joint cluster (green) of green strains and outlier strains (data set 8). b) shows the gene of a green strain and c) shows the gene of an outlier assigned to the green population. The unknown origin is represented by yellow color. d) A gene from an outlier assigned to the joint cluster (blue) of all outlier strains (data set 9). The unknown origin is represented by magenta color. e) A gene from an outlier, assigned to the joint cluster of all outliers (green). Section 85-235 is obtained from the red population (data set 10).

1.4 Summary of the elementary simulations

Statistical behavior of the introduced methodology was here investigated with 10 di¤erent data sets generated using a fairly simple forward simulation strategy. As a summary, it can be concluded that, as long as reasonably sized samples from potential source populations for a recombinant fragment are present in data, the method has high power of identifying the correct genome structure. The method is also able to detect parts of genome coming from sources outside of the known populations. However, the unknown nucleotide frequencies of the source population increase the uncertainty related to the estimation in such a situation. If some strain in the data set is the sole representative of its true population, it is possible that the strain will be assigned in the clustering step to some other cluster (e.g. 'hybrid cluster'), unless the molecular distance to other populations present in the data is high enough. In such a situation, the recombination profile usually assigns relatively low probabilities for the cluster to which the strain was assigned. Also, several small fragments for which an outside origin is assigned high probabilities, can usually be found. Thus, if the results contain areas where an outside origin has high probabilities, this may be an indication of either a true recombination from an outside source or that the strain should be assigned as a whole to a new cluster. Making the distinction between these two cases requires an investigation of the results for all the genes of the strain as a whole. If there are several short areas where an outside origin is assigned high values, and other areas where the population to which the strain was allocated in the clustering phase has relatively low probabilities, then the latter interpretation would more likely be proper. If, on the other hand, the population to which the strain was allocated in the clustering phase explains the observed sequences in general adequately, and there are only some areas with clear boundaries of having originated from the outside, then a true recombination is a more plausible explanation. These observations highlight the importance of using all the available information in making correct interpretations. Also, the compatibility of di¤erent interpretations with biological background knowledge should be taken into account.

2 Illustration of e¤ects of parameter values and used approximations

2.1 E¤ect of parameters L and L_{max}

Two parameters, L, and L_{max} specify the lower and the upper bounds for the length of intervals which are considered when calculating the marginal probabilities for the origin X_i of some base i in a gene. Figure 4 shows the e¤ect of using di¤erent values for these parameters. The analyzed gene is the first gene of the *B.cenocepacia IIIC* strain considered in detail in the real data analysis section. The length of the gene is 443 bases. When L is equal to unity, the marginal probability profile fluctuates strongly, and local features get much weight

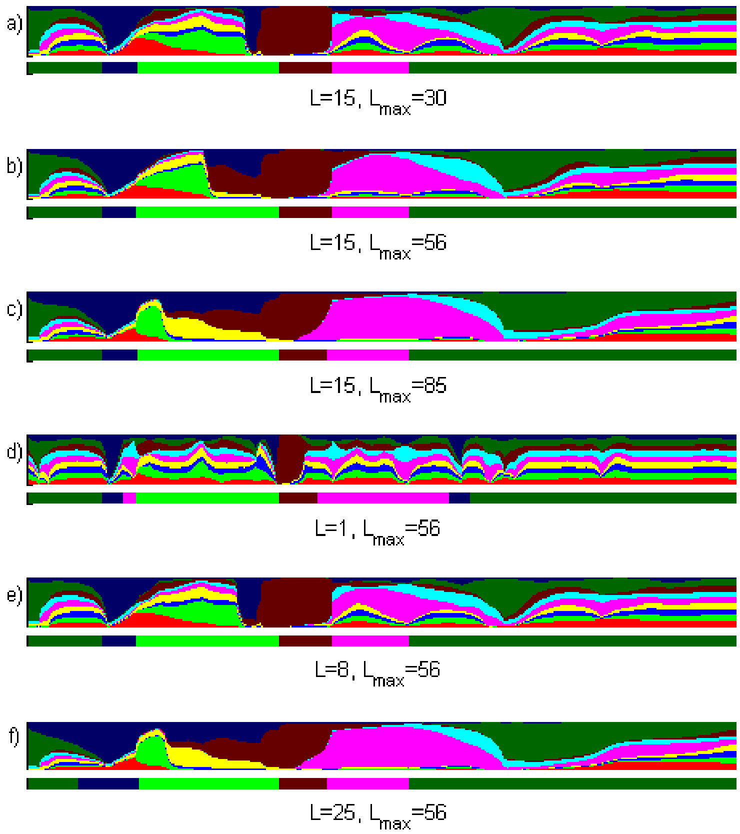

Figure 4: Illustration of the e¤ect of di¤erent parameter values for the lower and upper bounds, L and L_{max}, of the considered segment length. The parameter values are shown below the plots.

(Figure 4d). Also, if there is no strong evidence towards any of the clusters in the immediate neighborhood of some base, all the clusters get even probabilities for the origin of the base. Increasing L to 8 already provides an improvement by giving less weight to local features. Increasing L further to 15 or 25 smooths out some local variation, but the outcome is not significantly di¤erent from the case with $L = 8$. The e¤ect of L_{max} is similar to L: when L_{max} is increased, the local features get less weight and the areas with strong evidence to some origin spread out. However, except for the case with $L = 1$, all the marginal probability profiles look very similar in general, and the resulting optimal models are the same, except for slight changes in the lengths of the segments with $L = 25$.

2.2 Effect of approximation for intervals outside the segment

In the model formulation we approximated the marginal likelihood of data outside the interval $[a,b]$ under consideration by assigning the segments $[1,a-1]$ and $[b+1,n_g]$ to their optimal origins (formulas (11) and (12) in the main text). To investigate the effect of this 'crude' approximation to the calculated marginal probabilities of X_j, we use the following approach: we calculate the marginal likelihood for interval $[1,a-1]$ by

$$p\left(D_{[1,a-1]}\right) \approx \sum_{(\rho_{[1,a-1]},Z_{[1,a-1]})\in \mathbf{A}} p\left(D_{[1,a-1]}|\rho_{[1,a-1]},Z_{[1,a-1]}\right) p\left(\rho_{[1,a-1]},Z_{[1,a-1]}\right),$$

where $\mathbf{A}$ is the set of all the possible legitimate models $(\rho_{[1,a-1]},Z_{[1,a-1]})$ which have maximum three segments. The marginal likelihood for interval $[b+1,n_g]$ is calculated similarly. Thus, as opposed to the crude approximation strategy where the marginal likelihood for interval $[1,a-1]$ is based on a single model with one segment assigned to a single origin, we consider here all possible models with one, two or three segments assigned to any combination of putative origins. This is not yet the exact value for the marginal likelihood, since it is possible that the true best model(s) for interval $[1,a-1]$ contain even more than three segments. However, as compared to the crude approximation, this strategy can be considered to yield a value much closer to the exact value, and we will therefore refer to it as 'exact' in the sequel.

To illustrate the difference between the crude and the exact strategies, we will analyze four strains with both the approaches. As enumeration of all possible models for intervals $[1,a-1]$ and $[b+1,n_g]$ is computationally very demanding, we are obliged to restrict the comparison to subsequences of length of about half of the true lengths of the considered genes. In this way, we will analyze bases 1-200 of the first gene of *B. cenocepacia IIIC* and the fourth gene of *B. cenocepacia IIIB* strains, considered in the real data analysis in the main text. We will also analyze bases 1-150 of strains #27 and #30 from the coalescent simulation.

The results of all these analyses are shown in Figure 5. The *B. cenocepacia IIIB* strain and the strain #27 from the coalescent simulation correspond to cases in which the detected optimal model consists only of two segments. Thus, the crude approximation is expected to cause less bias than when the optimal model consists of several segments (and consequently also the optimal models for $[1,a-1]$ and $[b+1,n_g]$ can be expected to consist of more than one segment). By inspecting the results for these strains in Figure 5 a-d, one can see that the difference between the crude and the exact strategies is indeed very small or even negligible.

The detected optimal profiles of the *B. cenocepacia IIIC* strain and the strain #30 from the coalescent simulation consist of several fragments. Therefore, it might be expected that the difference between the crude and the exact strategies would be larger. However, by inspecting Figures 5 e-h, one can see that the

calculated marginal probability profiles are still highly similar with both the approaches. Nevertheless, comparison of 5g and 5h reveals that it is possible that the slight differences in the probability profiles (which are used to define a starting point for the search of the optimal model) may lead to slightly different outcomes when estimating the optimal model, if there are several alternative models with about equal probabilities.

3 Detailed description of real data analysis

B. cenocepacia IIIC. Before applying the introduced method to the *Burkholderia* data, we analyzed the data using the linkage admixture module of the BAPS program. As opposed to the methodology introduced here, BAPS model considers the genome as a whole to estimate the appropriate weights for the admixture proportions corresponding to the different ancestral origins, and does not reflect the actual locations of the recombined areas in the sequences. For this reason, it is also possible that BAPS may fail to identify some recombination events, even if there is a strong signal within a short interval of bases, but the signal is not significant on the level of the complete sequence. The BAPS analysis identified five strains as having statistically significant evidence for admixture. In particular, both strains from species *B. cenocepacia IIIC* were identified.

As our first example, we consider in detail one of the admixed *B. cenocepacia IIIC* strains. In total six of the clusters were associated with non-zero estimated admixture coefficients for this strain in the BAPS analysis, the estimates being: Cluster 1: 0.05, Cluster 2: 0.08, Cluster 4: 0.59, Cluster 5: 0.02, Cluster 6: 0.03, and Cluster 7: 0.23. The recombination profile obtained by the introduced method is shown in Figure 6. Immediately, areas in the sequence having a particular cluster as the most likely origin, are identified for each cluster associated with non-zero coefficients in the BAPS admixture analysis. Furthermore, some segments are also assigned to Cluster 8, and the cluster correponding to an unknown origin.

Certain details of the estimated sequence structure are now provided for the three main ancestral sources identified in the BAPS analysis (clusters 2, 4, 7). Cluster 7, containing the species *B. cenocepacia IIIA* and *IIIB*, was associated with the second largest admixture coefficient, and the marginal probability profile shows strong support for this origin at many sites. In particular, the third and the sixth gene have long areas where this origin dominates the probabilities. Also, in the first gene, around 160th base, the probability is conclusive for this cluster. In the interval of bases $[158, 190]$, the average distance of the strain to the cluster 7 is 0.65 mutations, while for example the average distances to the other two main clusters are 3.7 and 5 mutations.

Similarly, there are areas in sequence where the strongly supported origin corresponds to the cluster 2, associated with the third largest admixture coefficient and containing species *B. stabilis* and *B. pyrrocinia*. For example, in the end of the fifth gene within the interval $[319, 397]$, the average distance of the

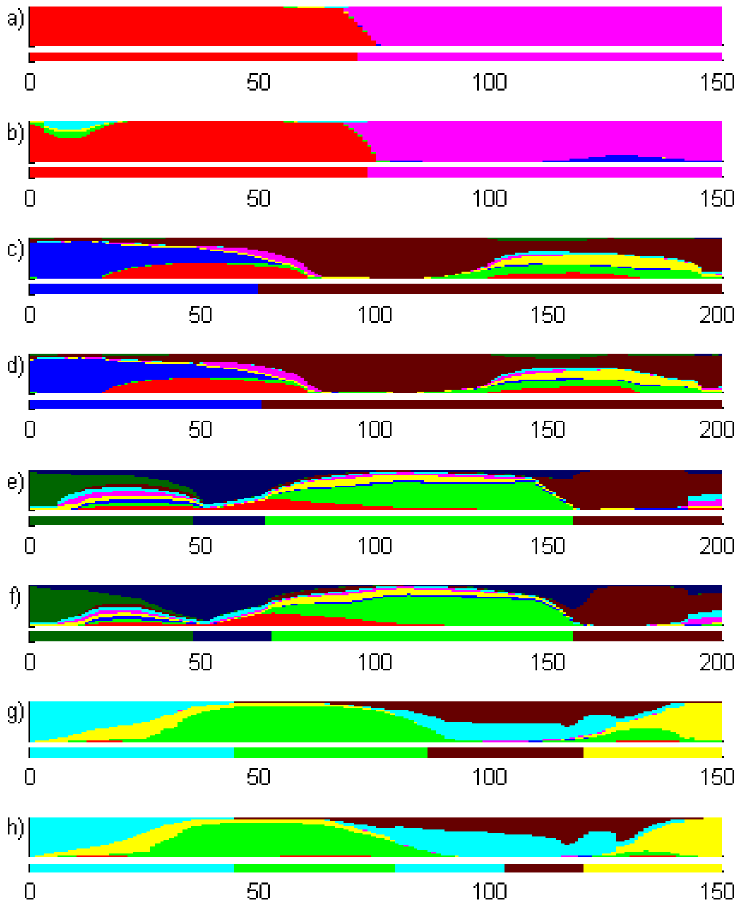

Figure 5: Results for four di¤erent strains when they are analyzed by using the crude approximation and exact enumeration strategies. The results are shown in pairs, such that the crude results precede the exact results. a), b) strain #27 from the coalescent simulation. c), d) *B. cenocepacia IIIB* strain. e), f) *B. cenocepacia IIIC* strain. g), h) strain #30 from the coalescent simulation.

strain to the cluster 2 is 4.2 mutations, while for any other cluster, the distance is at least 14.2 mutations. Interestingly, when the areas assigned to the cluster with the largest admixture coe¢cient in the BAPS analysis (cluster 4) are considered, it is not possible to identify as conclusive evidence as for the other two main clusters. For instance, the recombination model assigns the second gene as a whole to the origin corresponding to the cluster 4. However, when the distances of the strain to di¤erent clusters are calculated using the second gene, it is seen that actually the cluster 7, not cluster 4, is closest to the strain. The corresponding distances to the two clusters are 9.6 and 11.8 mutations, respectively. The reason why the model favors cluster 4 despite of this apparent discrepancy, is that cluster 4 contains more variation than cluster 7, the average distances between strains being 9.0 and 4.0 mutations, respectively. Thus, in this sense, the gene fits more plausibly to cluster 4.

The other clusters suggested by the optimal recombination profile as possible origins for some parts of the sequence were also investigated in the same manner as above. Analogous findings were obtained, namely that while the distances (in mutations) were somewhat supporting the suggested origin, no conclusive evidence was found. To focus on the biological interpretation for this strain we recall that the strain was assigned in the to a cluster (4), which consists of strains from species *B. cenocepacia IIIC*, *IIID*, *B. antina* and a group named as others. Furthermore, many of these species are represented by only a small number of strains. Thus, it is possible that cluster 4 is an example of what was earlier referred to as a 'hybrid cluster', i.e. a collection of strains from species which are represented in the data set by an insu¢cient number of strains to be identified as a distinct cluster. Such a misleading classification for the strain under investigation is also suggested by the fact that there are some areas in the sequence where the cluster corresponding to the unknown origin gets high probabilities.

Thus, we can summarize our findings for this strain as follows. There is strong evidence that some parts of the sequence share ancestry with cluster 7 (*B. cenocepacia IIIA* and *IIIB*) and some parts with cluster 2 (*B. stabilis* and *B. pyrrocinia*). Furthermore, there is strong evidence that there are areas in the sequence which are not closely related to either of the mentioned clusters. These areas are mostly represented by cluster 4, and some other clusters. However, there is no strong evidence that these clusters in fact represent the true origin for these areas, they may just be the statistically most appropriate ones of the available alternatives.

B. cenocepacia IIIA and IIIB. In the mixture clustering, the subspecies *IIIA* and *IIIB* of *B. cenocepacia* were clustered together, which reflects their close evolutionary relation. In the BAPS admixture analysis none of these strains was identified as having admixture. However, the recombination profiles calculated for the strains support a di¤erent conclusion. Furthermore, there is a clear distinction in the recombination profiles between the strains of the two groups. Out of the ten *IIIA* strains, only two had one recombination event in one gene in their optimal recombination model. On the other hand, all the ten *IIIB* strains had at least one recombinant segment in their optimal model profiles, and

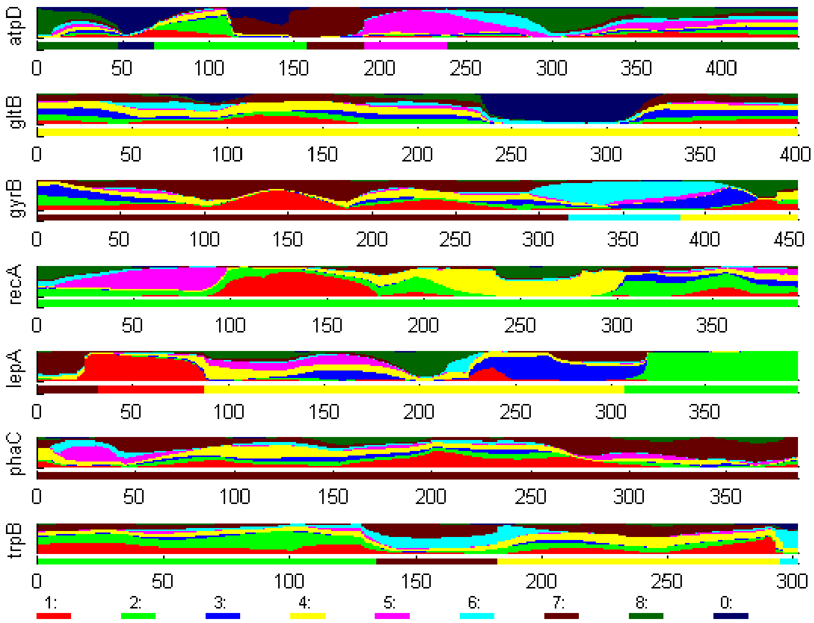

Figure 6: The recombination profile of the strain belonging to *B. cenocepacia IIIC*, discussed in the text.

most of these strains had actually a couple of such segments. These segments were mostly assigned to the clusters corresponding to species *B. ambifaria* and *B. vietnamiensis*. Figure 7 shows the profile of one strain belonging to *IIIB*. The segment assigned to *B. ambifaria* can be seen at the beginning of the fourth gene (blue color). When we investigated the average distance of this strain to di¤erent clusters within this region, it could be concluded that the segment in the interval [1, 73] is exactly the same as for all the strains in *B. ambifaria* species. The second closest cluster to the strain in this area is the cluster to which the strain was assigned in the mixture clustering (i.e. cluster containing other strains of *B. cenocepacia IIIA* and *IIIB*), and on average the strains in this cluster are at a distance of 2.4 mutations from the strain. All other clusters are on average more than 3.8 mutations away from this strain. We did similar calculations to the segment assigned to the *B. vietnamiensis* species, which can be seen at the end of the third gene. In this region, corresponding to the bases in the interval [341, 454], the strains of *B. vietnamiensis* are on average at a distance of 0.07 mutations from this strain, while the second closest is the cluster corresponding to *B. cepacia*, the strains of which are on average at the distance of 1.6 mutations of this strain. The cluster to which the strain was assigned in the mixture clustering is within this region on average 4.3 mutations away from this strain. These calculations strongly support the conclusion that the investigated intervals have their origins in a di¤erent source than the rest of the sequence.

Similar calculations with analogous conclusions can be made from other suggested recombinant segments from *B. ambifaria* and *B. vietnamiensis* for the strains of species *B. cenocepacia IIIB*. For some of the strains in *B. cenocepacia IIIB* the optimal profiles contained some recombinant segments from other sources also. However, when we performed similar calculations for these segments, the results were not as conclusive, as for the segments assigned to *B. ambifaria* and *B. vietnamiensis.* The same applies to the two possible recombinant segments which were observed in the optimal profiles of *B. cenocepacia IIIA*. Thus, the biological interpretation of these results would be that the di¤erence between *B. cenocepacia IIIA* and *B. cenocepacia IIIB* is that *IIIB* has had recombination with *B. ambifaria* and/or *B. vietnamiensis*, while *IIIA* has not. Further statistical support for the suggested recombinations is given by the fact that many of the strains in *IIIB* which contain some recombinant segment in the optimal profile from either *B. ambifaria* or *B. vietnamiensis,* have actually two or three such segments in di¤erent genes and locations. This would not be expected, if the detected recombinations were a result of random variation in the sequences.

B. pyrrocinia. The recombination profile of a strain belonging to *B. pyrrocinia* species is shown in Figure 8. This strain was not identified as admixed in the BAPS admixture analysis. However, the profile shows a striking feature which is not present in any other profile in the whole data set. Apart from some small stretches, the third gene is assigned as a whole to an unknown origin in the optimal model, i.e. origin not represented by any of the known clusters. This can also be seen, when the average distances to the di¤erent clus-

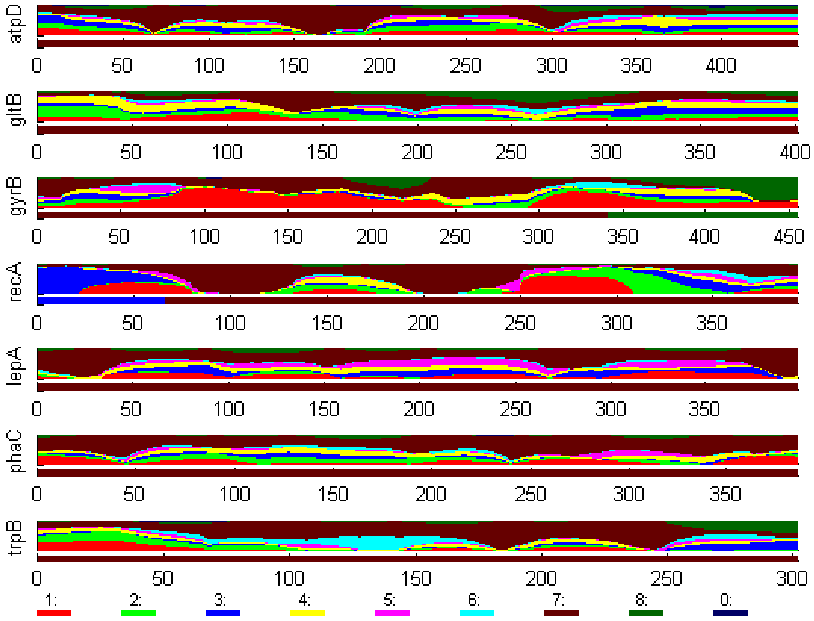

Figure 7: The recombination profile of the strain belonging to *B. cenocepacia* IIIB, discussed in the text

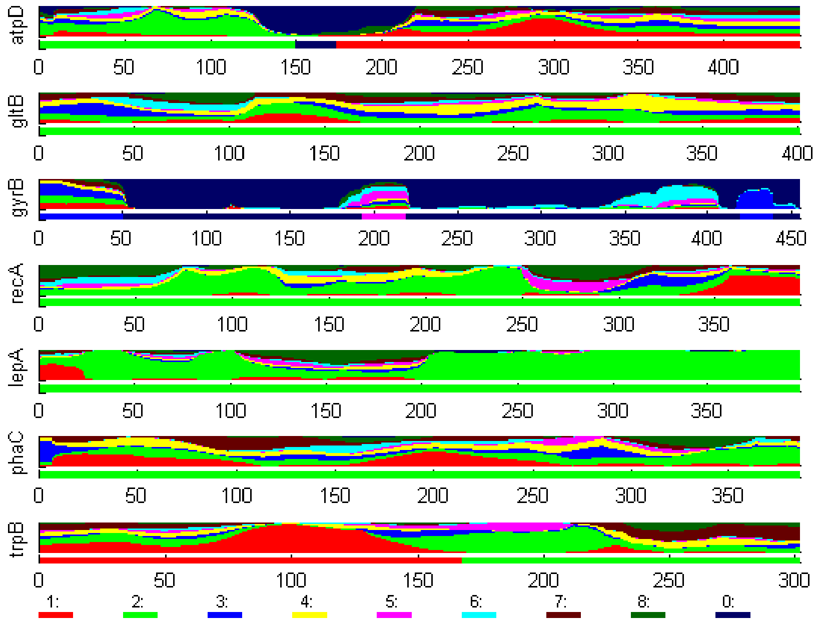

Figure 8: The recombination profile of the strain belonging to *B. pyrrocinia*, with the third gene assigned to an unknown origin.

ters are calculated. The average distance in this gene to the cluster closest to the strain is 81 mutations, while the average distance in this gene between the strains in any cluster is at most approximately 28 mutations. Thus, this gene is dozens of mutations further apart from any species present in the data. Because this is the only gene of the strain with this characteristic, a plausible biological explanation would be that this gene is obtained as a whole by recombination with some species not present in the data.
