# Supplementary figures and images for: Bayesian modeling of recombination events in bacterial populations
Source: BMC Bioinformatics. 2008 Oct 7;9:421. doi: 10.1186/1471-2105-9-421 (PMC2579306; doi:10.1186/1471-2105-9-421)

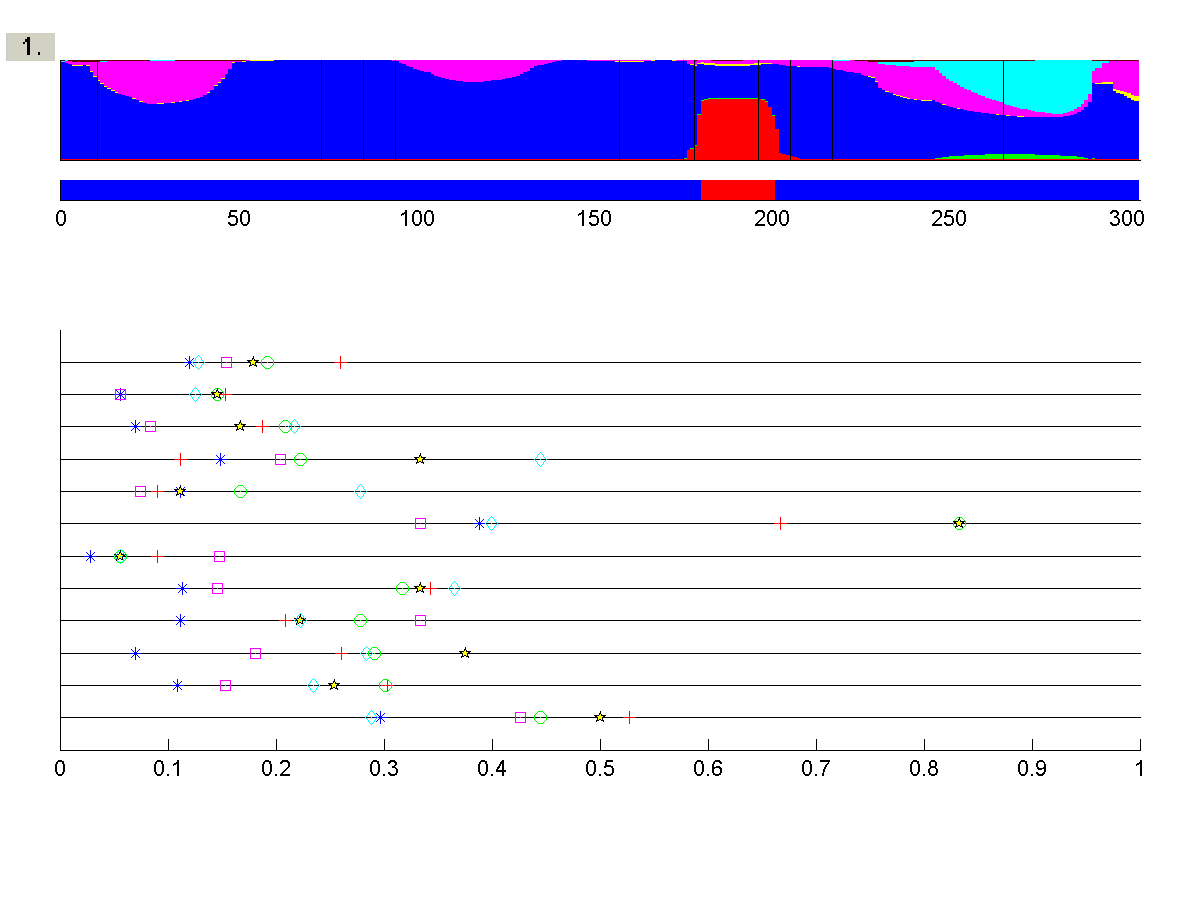

Supplement: Additional File 1 — Package of supplementary material. File BRAT_supplementary.zip available at includes a file BRAT_supplementary_text.pdf, which contains additional information on following issues: 1) elementary simulation experiments, 2) illustration of the effect of used parameter values and utilized approximations, and 3) a description of the real data analysis with more details than presented in the main text. The zip package also includes two folders. The folder "Coalescent_results" contains complete results for the analyzed coalescent data set, see the README file in the folder for further details. The folder "Trees for repetitive simulations" contains the figures of the left-side and right-side trees used in different types of simulations. [file 1471-2105-9-421-S1.zip › BRAT_supplementary_2/Coalescent_results/Results/strain1.png]

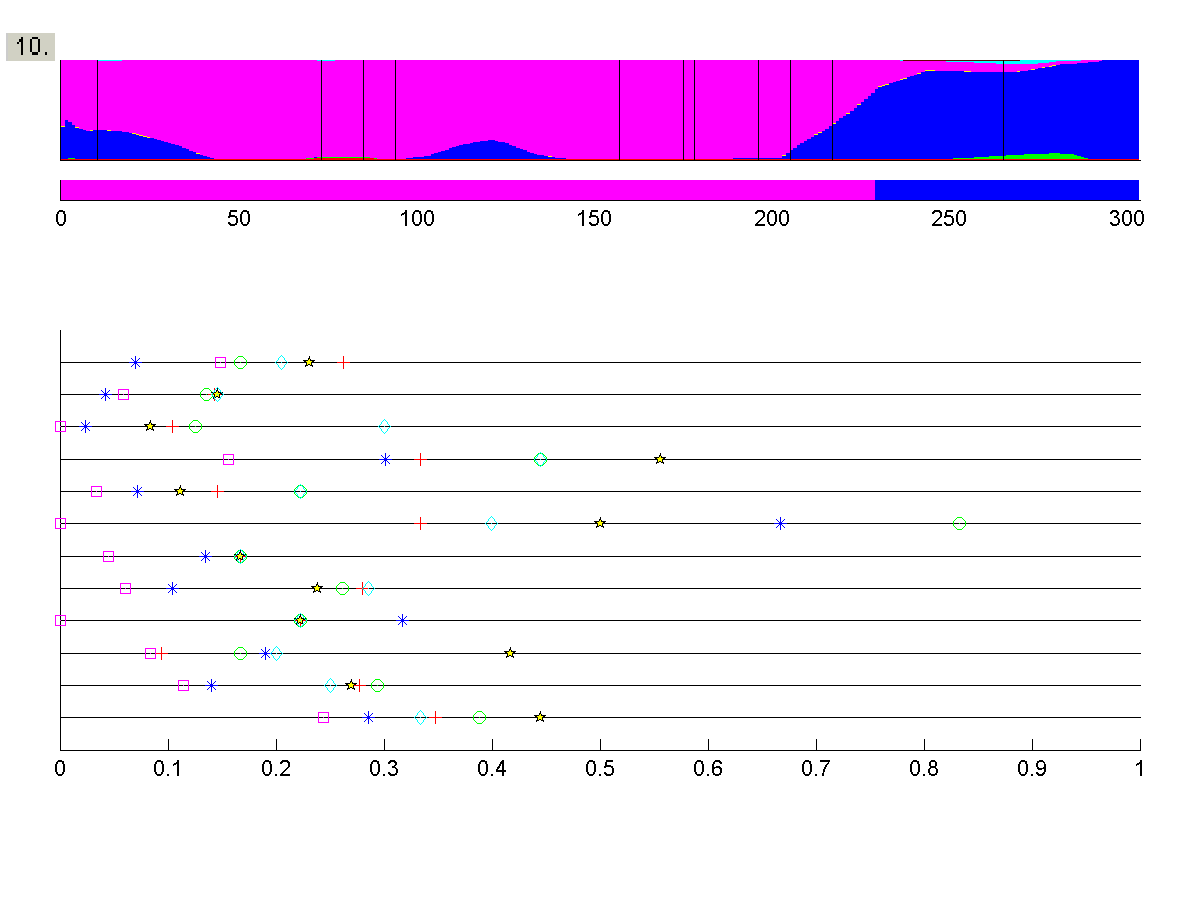

Supplement: Additional File 1 — Package of supplementary material. File BRAT_supplementary.zip available at includes a file BRAT_supplementary_text.pdf, which contains additional information on following issues: 1) elementary simulation experiments, 2) illustration of the effect of used parameter values and utilized approximations, and 3) a description of the real data analysis with more details than presented in the main text. The zip package also includes two folders. The folder "Coalescent_results" contains complete results for the analyzed coalescent data set, see the README file in the folder for further details. The folder "Trees for repetitive simulations" contains the figures of the left-side and right-side trees used in different types of simulations. [file 1471-2105-9-421-S1.zip › BRAT_supplementary_2/Coalescent_results/Results/strain10.png]

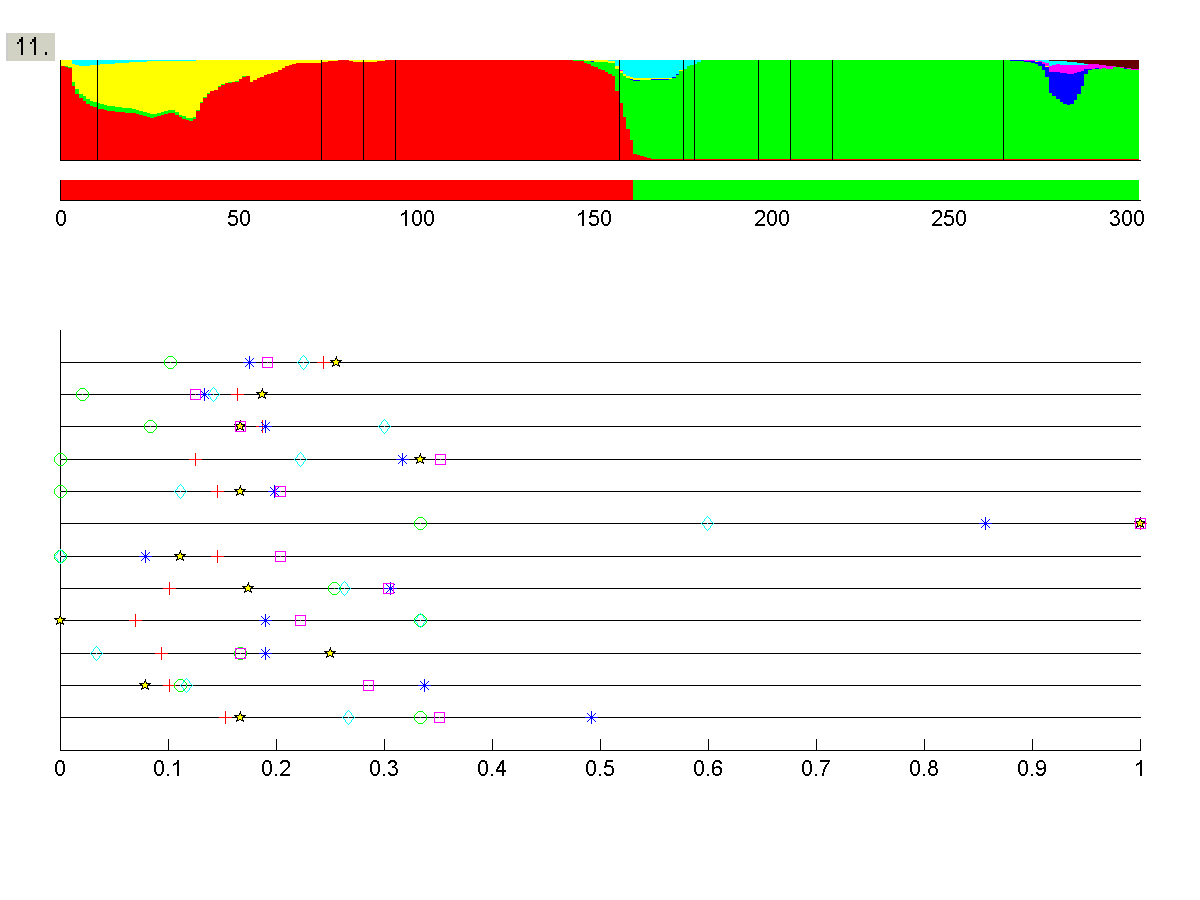

Supplement: Additional File 1 — Package of supplementary material. File BRAT_supplementary.zip available at includes a file BRAT_supplementary_text.pdf, which contains additional information on following issues: 1) elementary simulation experiments, 2) illustration of the effect of used parameter values and utilized approximations, and 3) a description of the real data analysis with more details than presented in the main text. The zip package also includes two folders. The folder "Coalescent_results" contains complete results for the analyzed coalescent data set, see the README file in the folder for further details. The folder "Trees for repetitive simulations" contains the figures of the left-side and right-side trees used in different types of simulations. [file 1471-2105-9-421-S1.zip › BRAT_supplementary_2/Coalescent_results/Results/strain11.png]

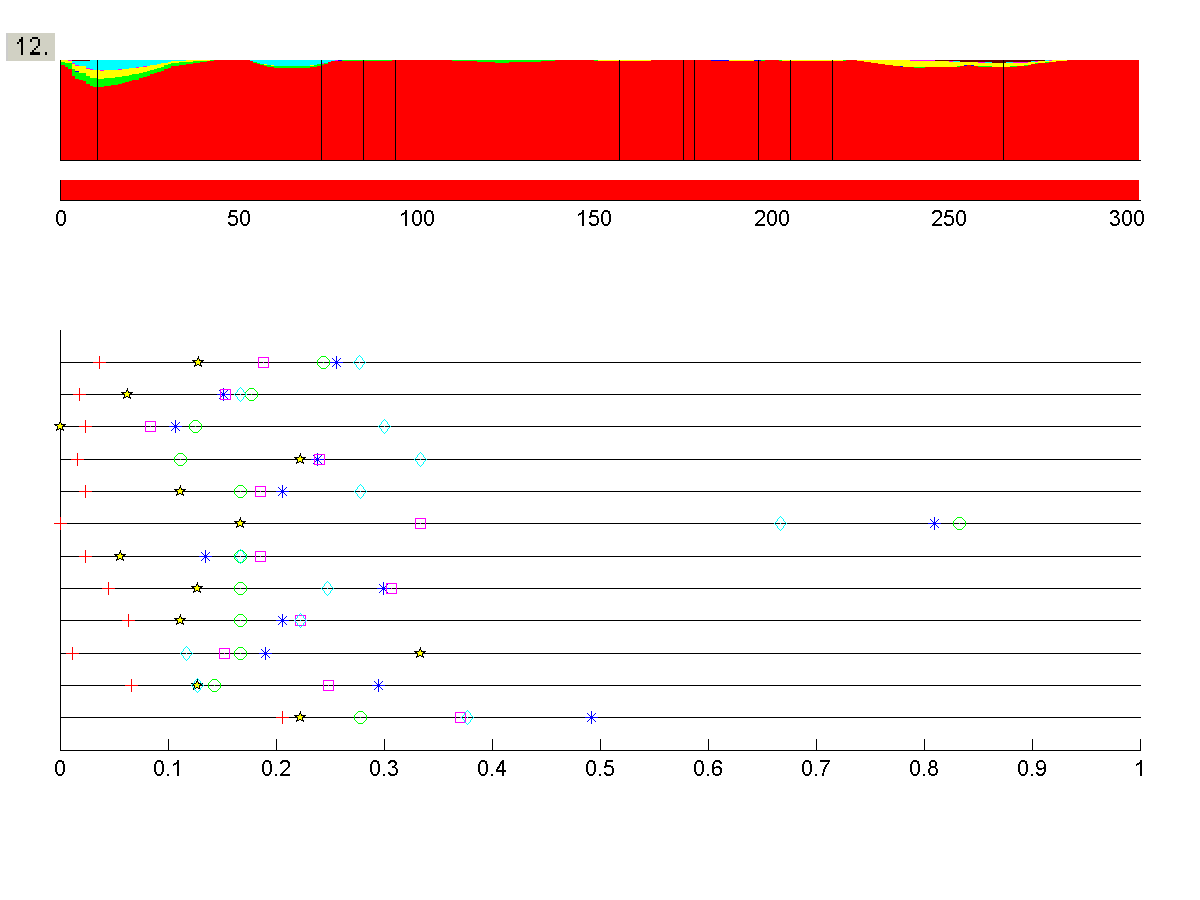

Supplement: Additional File 1 — Package of supplementary material. File BRAT_supplementary.zip available at includes a file BRAT_supplementary_text.pdf, which contains additional information on following issues: 1) elementary simulation experiments, 2) illustration of the effect of used parameter values and utilized approximations, and 3) a description of the real data analysis with more details than presented in the main text. The zip package also includes two folders. The folder "Coalescent_results" contains complete results for the analyzed coalescent data set, see the README file in the folder for further details. The folder "Trees for repetitive simulations" contains the figures of the left-side and right-side trees used in different types of simulations. [file 1471-2105-9-421-S1.zip › BRAT_supplementary_2/Coalescent_results/Results/strain12.png]

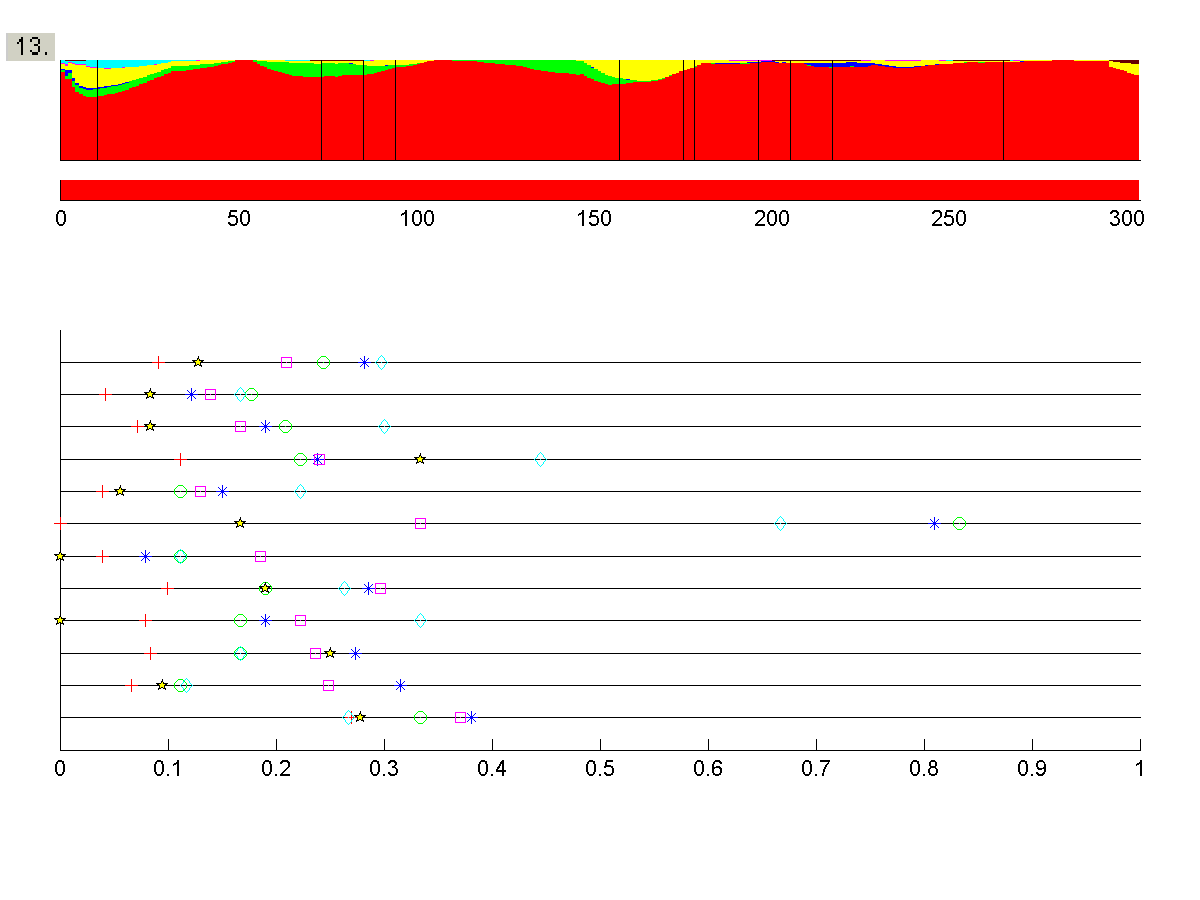

Supplement: Additional File 1 — Package of supplementary material. File BRAT_supplementary.zip available at includes a file BRAT_supplementary_text.pdf, which contains additional information on following issues: 1) elementary simulation experiments, 2) illustration of the effect of used parameter values and utilized approximations, and 3) a description of the real data analysis with more details than presented in the main text. The zip package also includes two folders. The folder "Coalescent_results" contains complete results for the analyzed coalescent data set, see the README file in the folder for further details. The folder "Trees for repetitive simulations" contains the figures of the left-side and right-side trees used in different types of simulations. [file 1471-2105-9-421-S1.zip › BRAT_supplementary_2/Coalescent_results/Results/strain13.png]

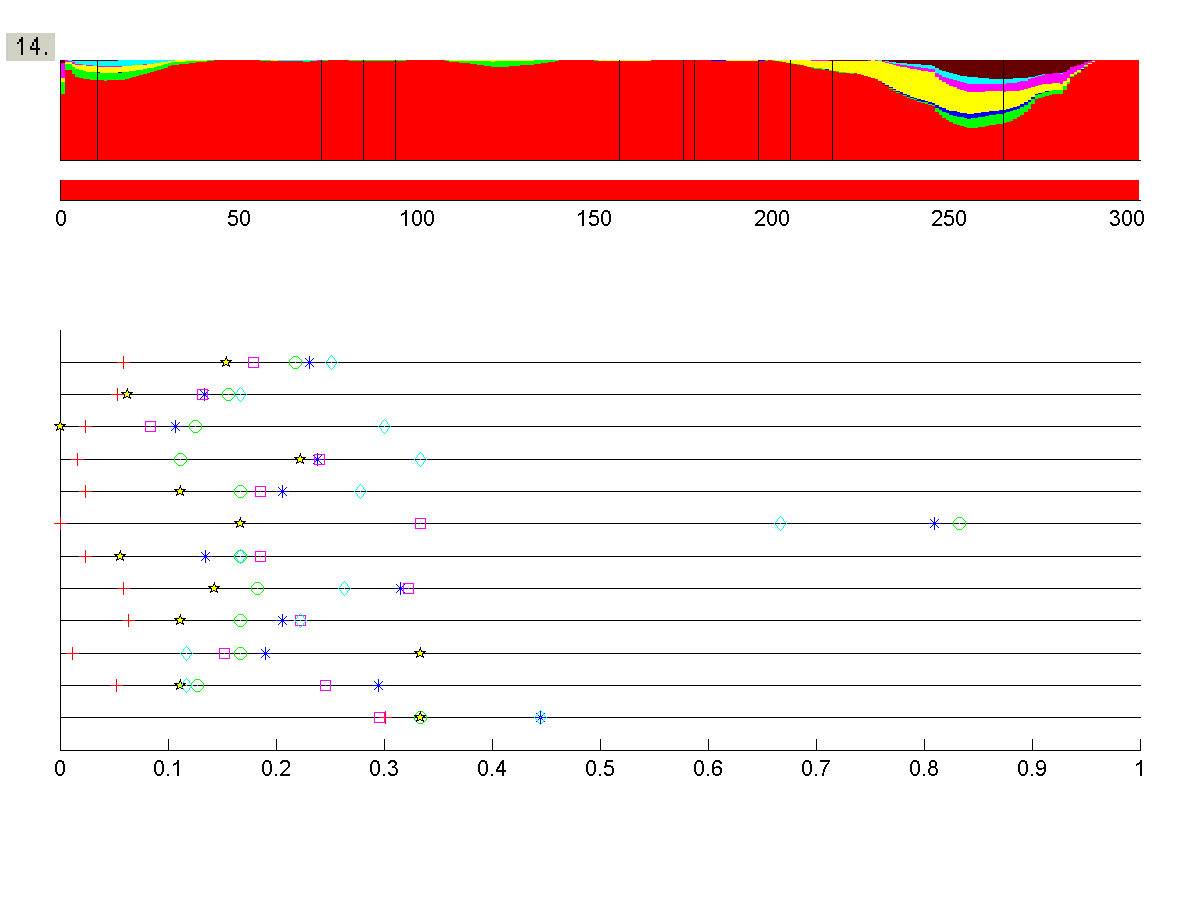

Supplement: Additional File 1 — Package of supplementary material. File BRAT_supplementary.zip available at includes a file BRAT_supplementary_text.pdf, which contains additional information on following issues: 1) elementary simulation experiments, 2) illustration of the effect of used parameter values and utilized approximations, and 3) a description of the real data analysis with more details than presented in the main text. The zip package also includes two folders. The folder "Coalescent_results" contains complete results for the analyzed coalescent data set, see the README file in the folder for further details. The folder "Trees for repetitive simulations" contains the figures of the left-side and right-side trees used in different types of simulations. [file 1471-2105-9-421-S1.zip › BRAT_supplementary_2/Coalescent_results/Results/strain14.png]

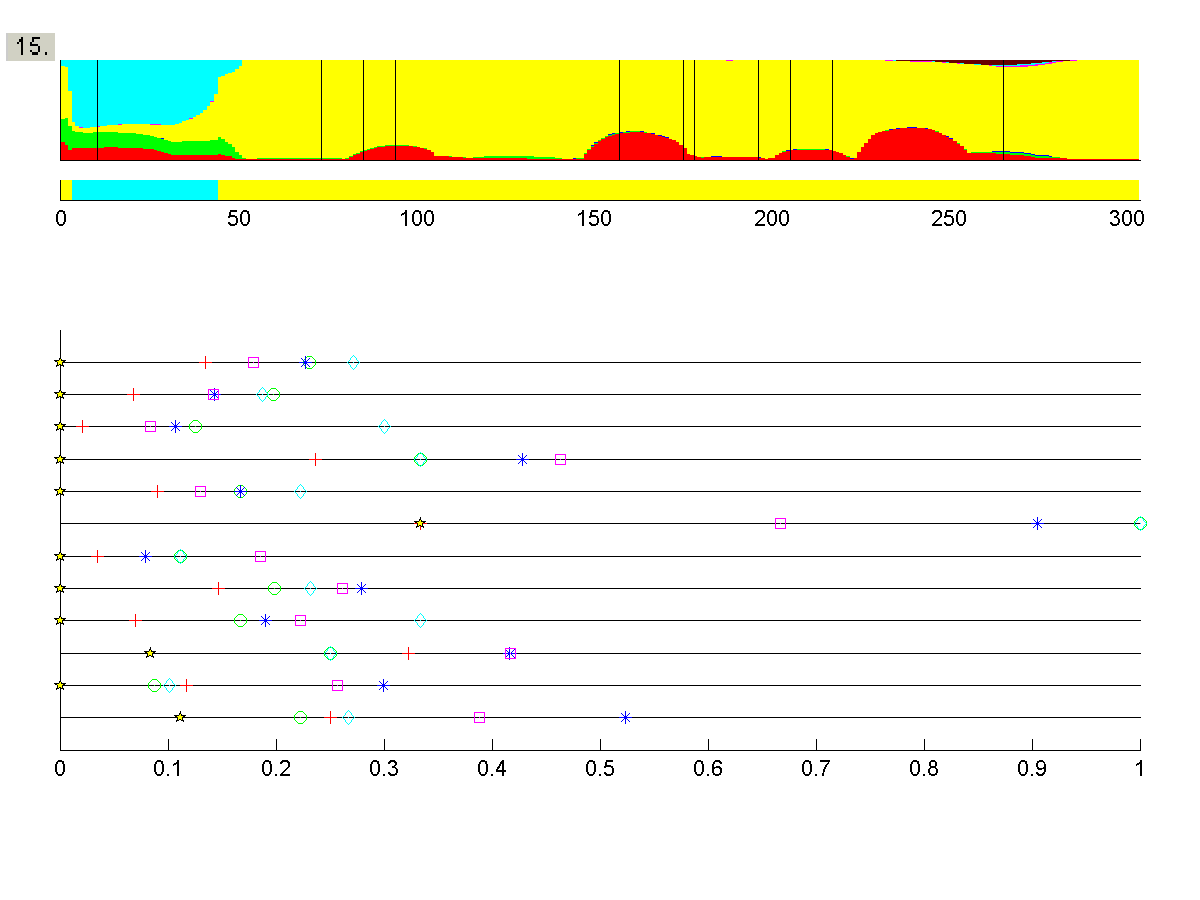

Supplement: Additional File 1 — Package of supplementary material. File BRAT_supplementary.zip available at includes a file BRAT_supplementary_text.pdf, which contains additional information on following issues: 1) elementary simulation experiments, 2) illustration of the effect of used parameter values and utilized approximations, and 3) a description of the real data analysis with more details than presented in the main text. The zip package also includes two folders. The folder "Coalescent_results" contains complete results for the analyzed coalescent data set, see the README file in the folder for further details. The folder "Trees for repetitive simulations" contains the figures of the left-side and right-side trees used in different types of simulations. [file 1471-2105-9-421-S1.zip › BRAT_supplementary_2/Coalescent_results/Results/strain15.png]

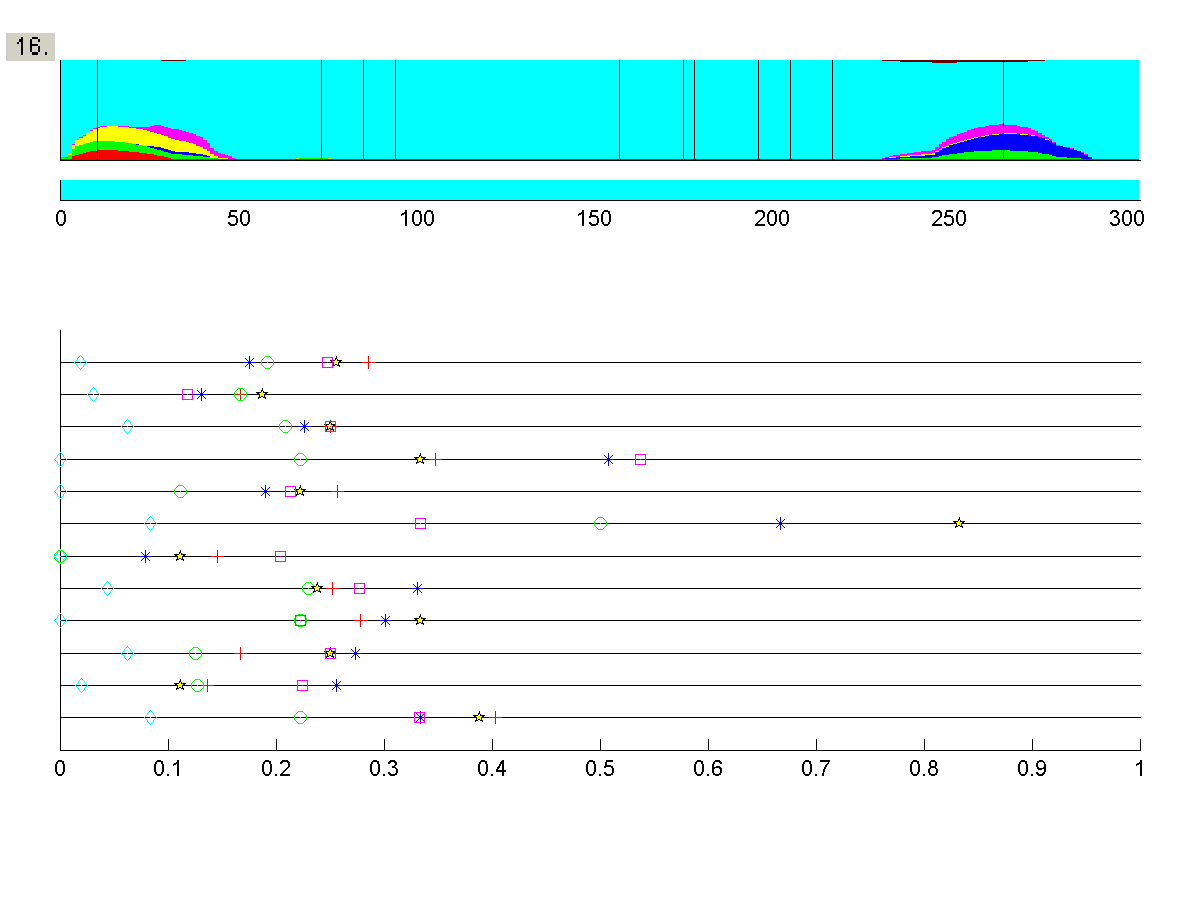

Supplement: Additional File 1 — Package of supplementary material. File BRAT_supplementary.zip available at includes a file BRAT_supplementary_text.pdf, which contains additional information on following issues: 1) elementary simulation experiments, 2) illustration of the effect of used parameter values and utilized approximations, and 3) a description of the real data analysis with more details than presented in the main text. The zip package also includes two folders. The folder "Coalescent_results" contains complete results for the analyzed coalescent data set, see the README file in the folder for further details. The folder "Trees for repetitive simulations" contains the figures of the left-side and right-side trees used in different types of simulations. [file 1471-2105-9-421-S1.zip › BRAT_supplementary_2/Coalescent_results/Results/strain16.png]

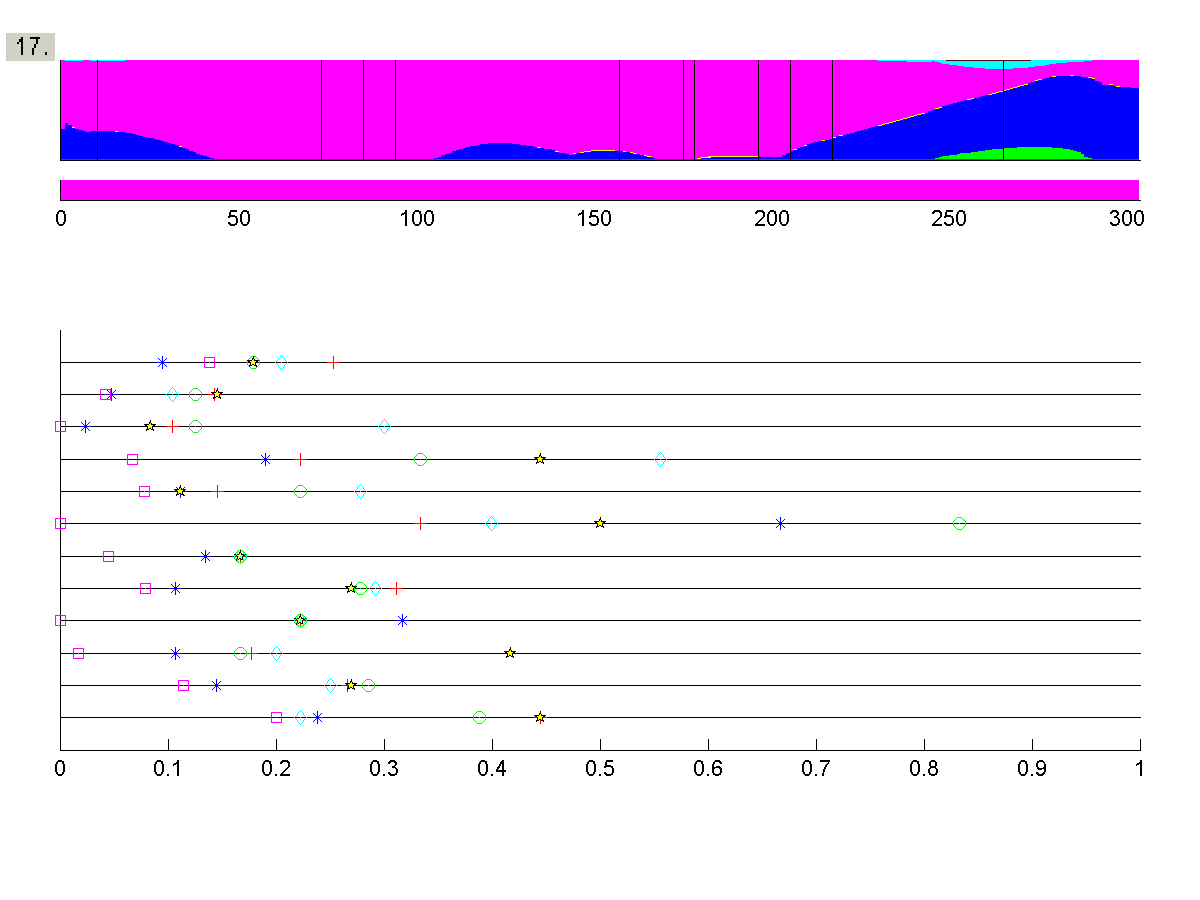

Supplement: Additional File 1 — Package of supplementary material. File BRAT_supplementary.zip available at includes a file BRAT_supplementary_text.pdf, which contains additional information on following issues: 1) elementary simulation experiments, 2) illustration of the effect of used parameter values and utilized approximations, and 3) a description of the real data analysis with more details than presented in the main text. The zip package also includes two folders. The folder "Coalescent_results" contains complete results for the analyzed coalescent data set, see the README file in the folder for further details. The folder "Trees for repetitive simulations" contains the figures of the left-side and right-side trees used in different types of simulations. [file 1471-2105-9-421-S1.zip › BRAT_supplementary_2/Coalescent_results/Results/strain17.png]

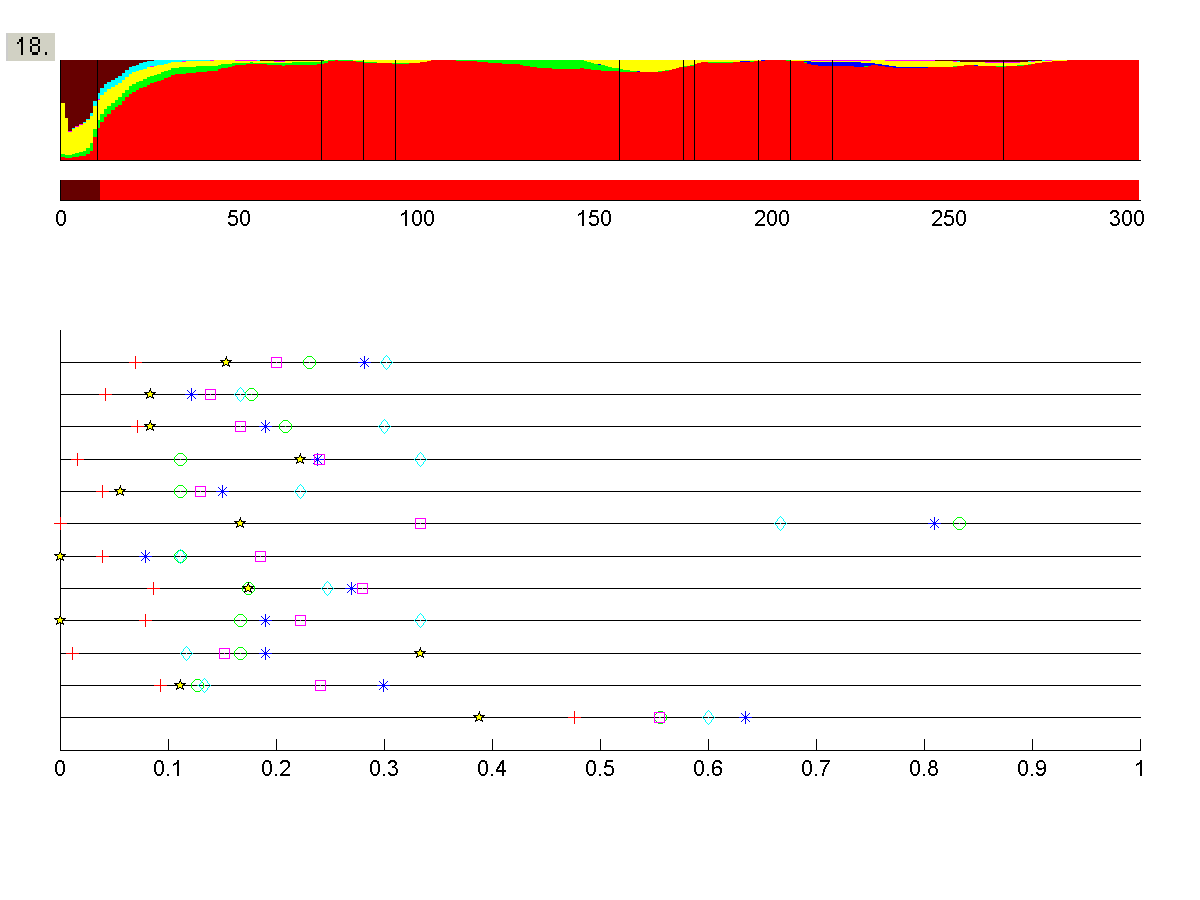

Supplement: Additional File 1 — Package of supplementary material. File BRAT_supplementary.zip available at includes a file BRAT_supplementary_text.pdf, which contains additional information on following issues: 1) elementary simulation experiments, 2) illustration of the effect of used parameter values and utilized approximations, and 3) a description of the real data analysis with more details than presented in the main text. The zip package also includes two folders. The folder "Coalescent_results" contains complete results for the analyzed coalescent data set, see the README file in the folder for further details. The folder "Trees for repetitive simulations" contains the figures of the left-side and right-side trees used in different types of simulations. [file 1471-2105-9-421-S1.zip › BRAT_supplementary_2/Coalescent_results/Results/strain18.png]

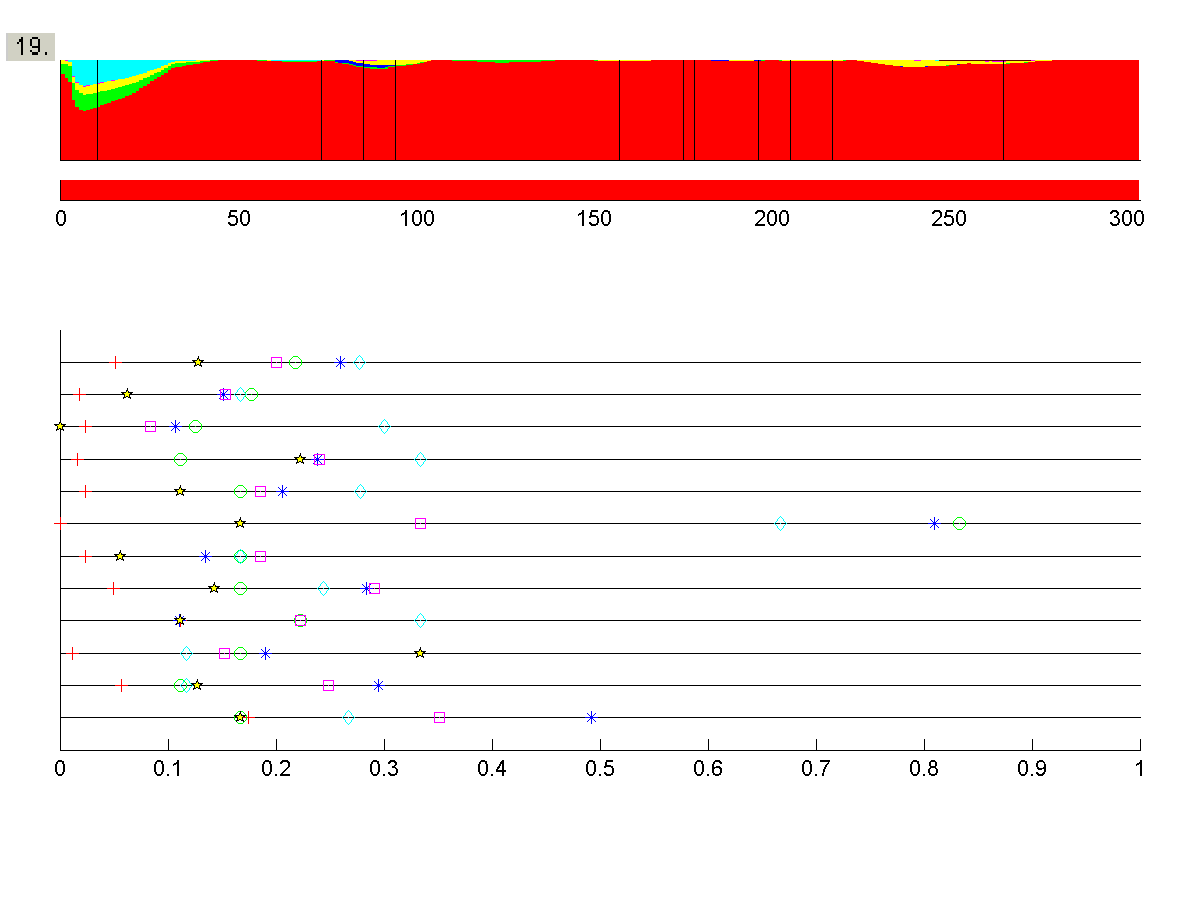

Supplement: Additional File 1 — Package of supplementary material. File BRAT_supplementary.zip available at includes a file BRAT_supplementary_text.pdf, which contains additional information on following issues: 1) elementary simulation experiments, 2) illustration of the effect of used parameter values and utilized approximations, and 3) a description of the real data analysis with more details than presented in the main text. The zip package also includes two folders. The folder "Coalescent_results" contains complete results for the analyzed coalescent data set, see the README file in the folder for further details. The folder "Trees for repetitive simulations" contains the figures of the left-side and right-side trees used in different types of simulations. [file 1471-2105-9-421-S1.zip › BRAT_supplementary_2/Coalescent_results/Results/strain19.png]

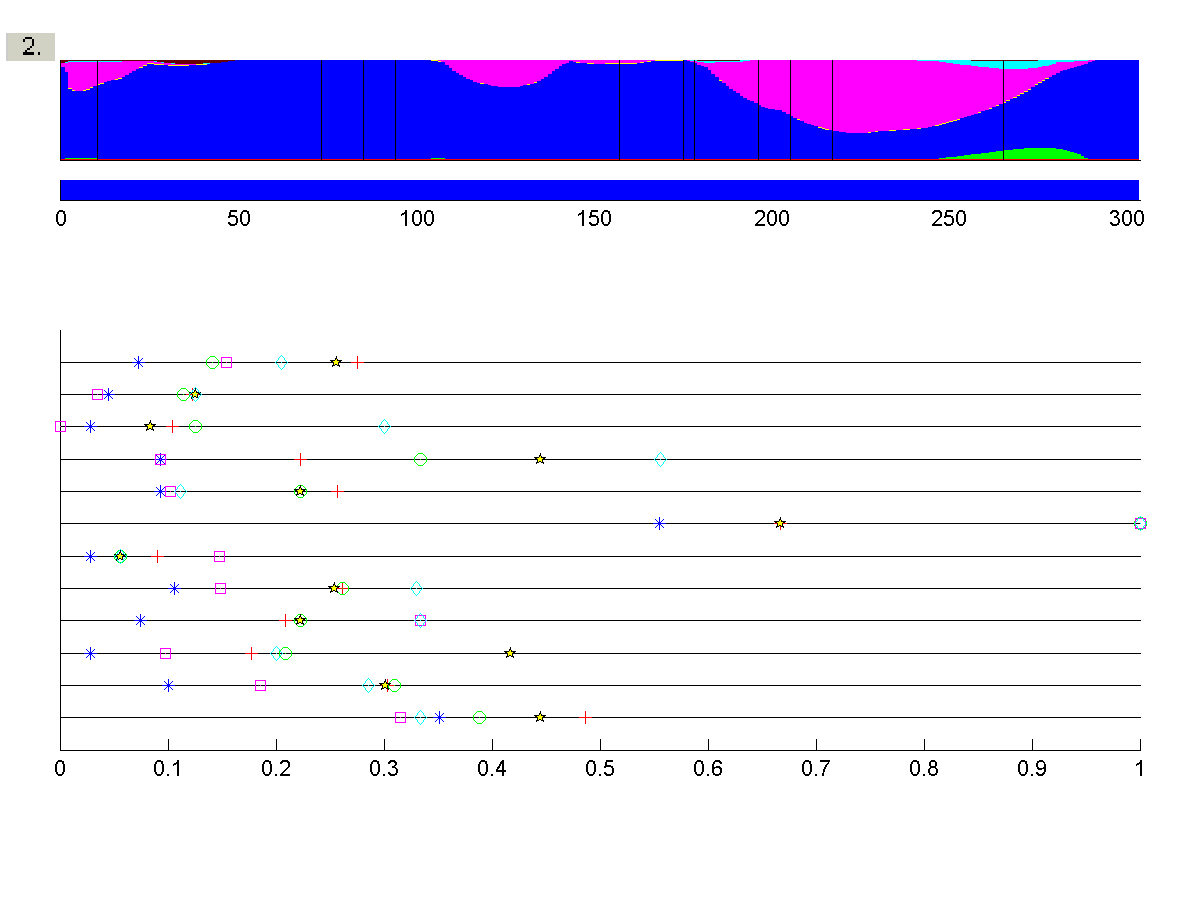

Supplement: Additional File 1 — Package of supplementary material. File BRAT_supplementary.zip available at includes a file BRAT_supplementary_text.pdf, which contains additional information on following issues: 1) elementary simulation experiments, 2) illustration of the effect of used parameter values and utilized approximations, and 3) a description of the real data analysis with more details than presented in the main text. The zip package also includes two folders. The folder "Coalescent_results" contains complete results for the analyzed coalescent data set, see the README file in the folder for further details. The folder "Trees for repetitive simulations" contains the figures of the left-side and right-side trees used in different types of simulations. [file 1471-2105-9-421-S1.zip › BRAT_supplementary_2/Coalescent_results/Results/strain2.png]

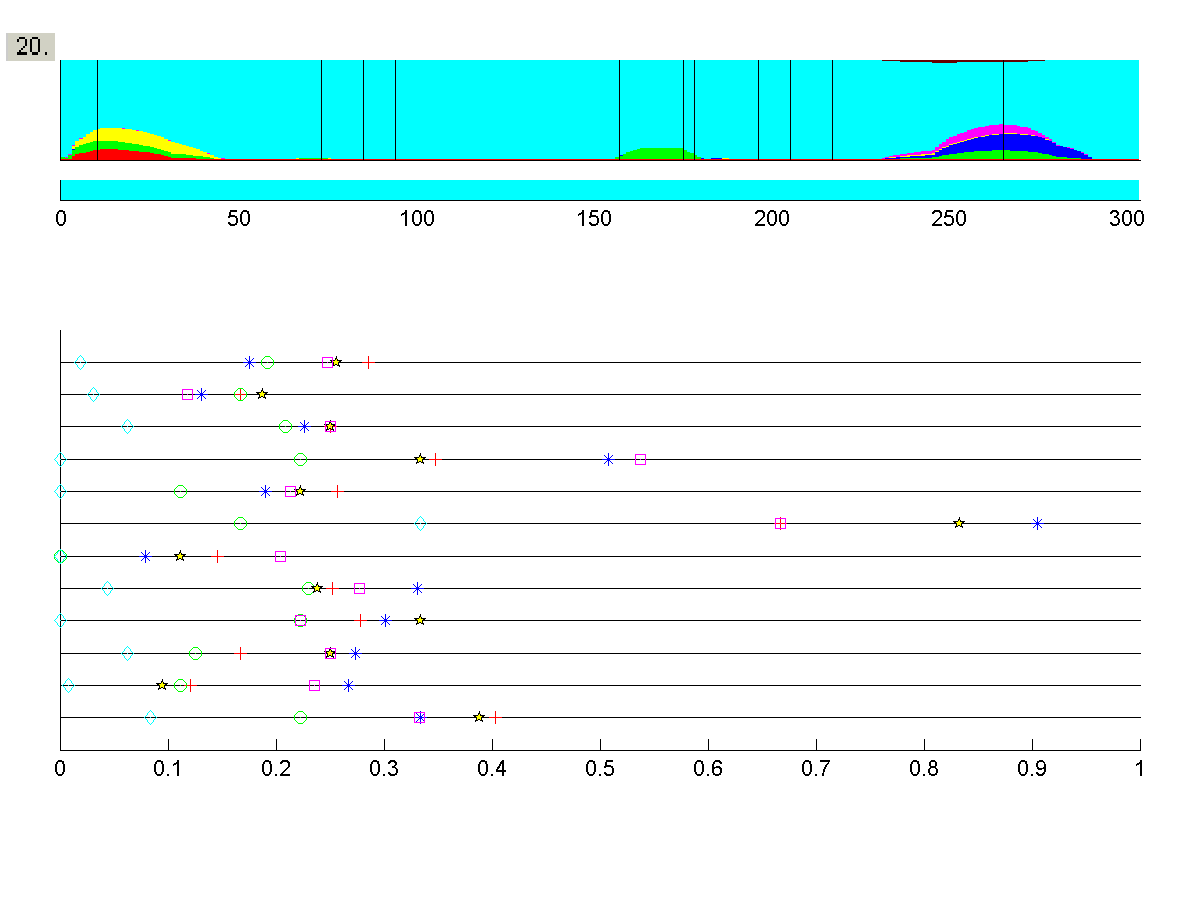

Supplement: Additional File 1 — Package of supplementary material. File BRAT_supplementary.zip available at includes a file BRAT_supplementary_text.pdf, which contains additional information on following issues: 1) elementary simulation experiments, 2) illustration of the effect of used parameter values and utilized approximations, and 3) a description of the real data analysis with more details than presented in the main text. The zip package also includes two folders. The folder "Coalescent_results" contains complete results for the analyzed coalescent data set, see the README file in the folder for further details. The folder "Trees for repetitive simulations" contains the figures of the left-side and right-side trees used in different types of simulations. [file 1471-2105-9-421-S1.zip › BRAT_supplementary_2/Coalescent_results/Results/strain20.png]

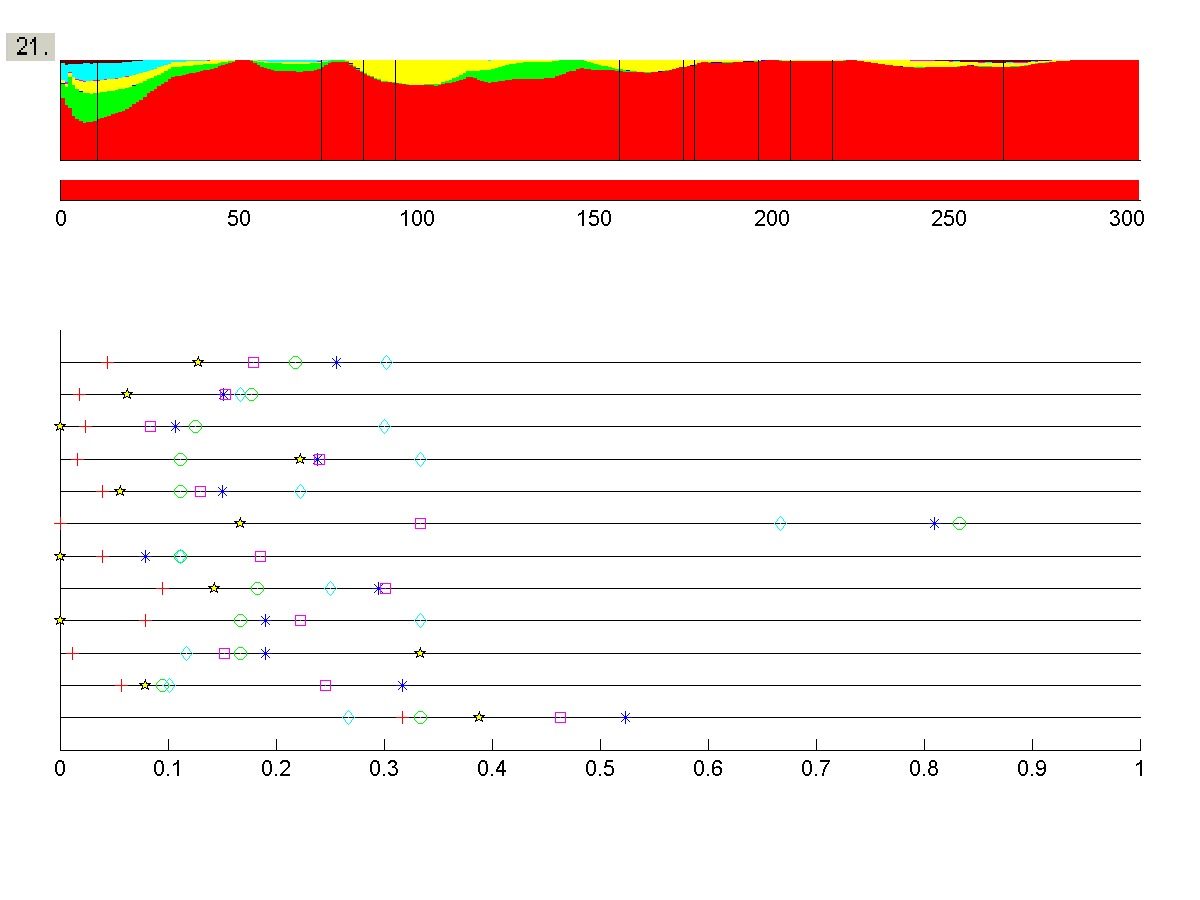

Supplement: Additional File 1 — Package of supplementary material. File BRAT_supplementary.zip available at includes a file BRAT_supplementary_text.pdf, which contains additional information on following issues: 1) elementary simulation experiments, 2) illustration of the effect of used parameter values and utilized approximations, and 3) a description of the real data analysis with more details than presented in the main text. The zip package also includes two folders. The folder "Coalescent_results" contains complete results for the analyzed coalescent data set, see the README file in the folder for further details. The folder "Trees for repetitive simulations" contains the figures of the left-side and right-side trees used in different types of simulations. [file 1471-2105-9-421-S1.zip › BRAT_supplementary_2/Coalescent_results/Results/strain21.png]

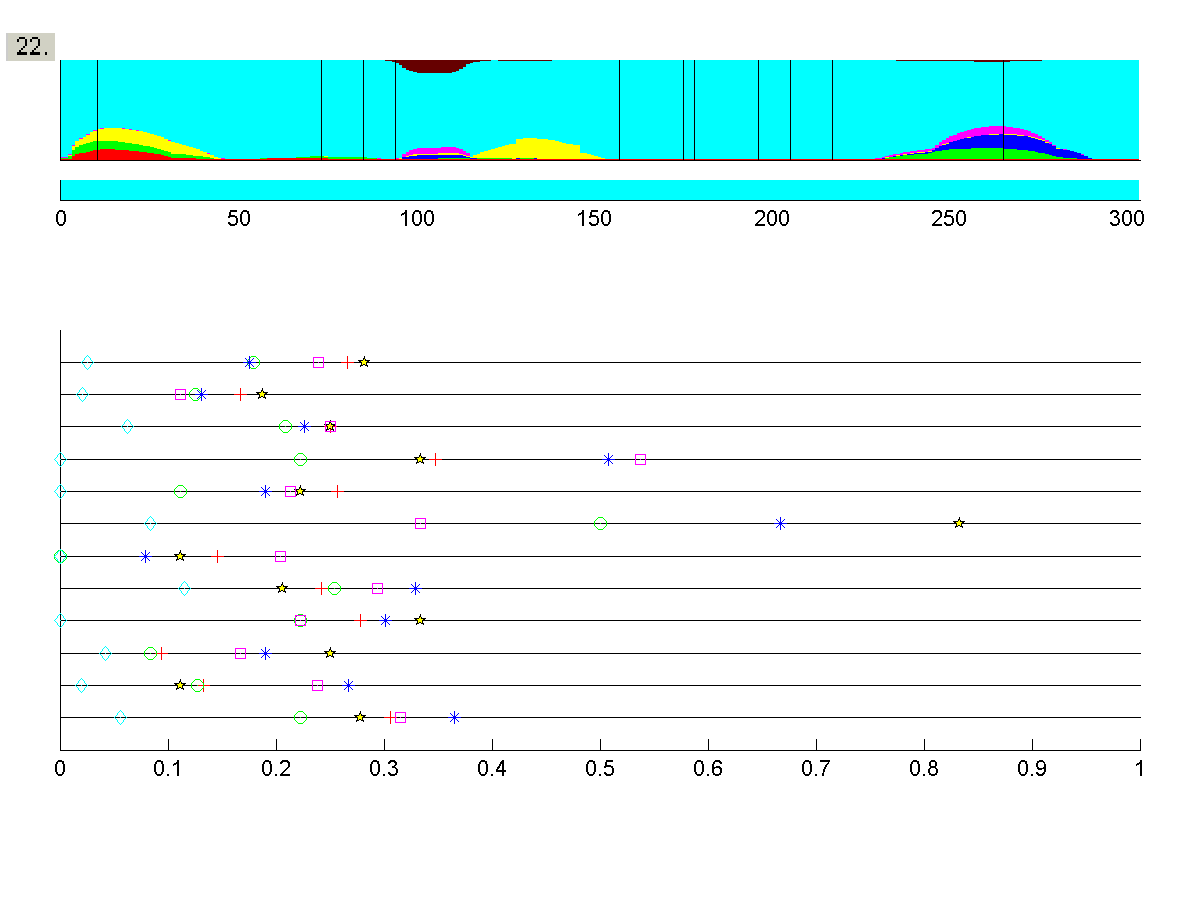

Supplement: Additional File 1 — Package of supplementary material. File BRAT_supplementary.zip available at includes a file BRAT_supplementary_text.pdf, which contains additional information on following issues: 1) elementary simulation experiments, 2) illustration of the effect of used parameter values and utilized approximations, and 3) a description of the real data analysis with more details than presented in the main text. The zip package also includes two folders. The folder "Coalescent_results" contains complete results for the analyzed coalescent data set, see the README file in the folder for further details. The folder "Trees for repetitive simulations" contains the figures of the left-side and right-side trees used in different types of simulations. [file 1471-2105-9-421-S1.zip › BRAT_supplementary_2/Coalescent_results/Results/strain22.png]

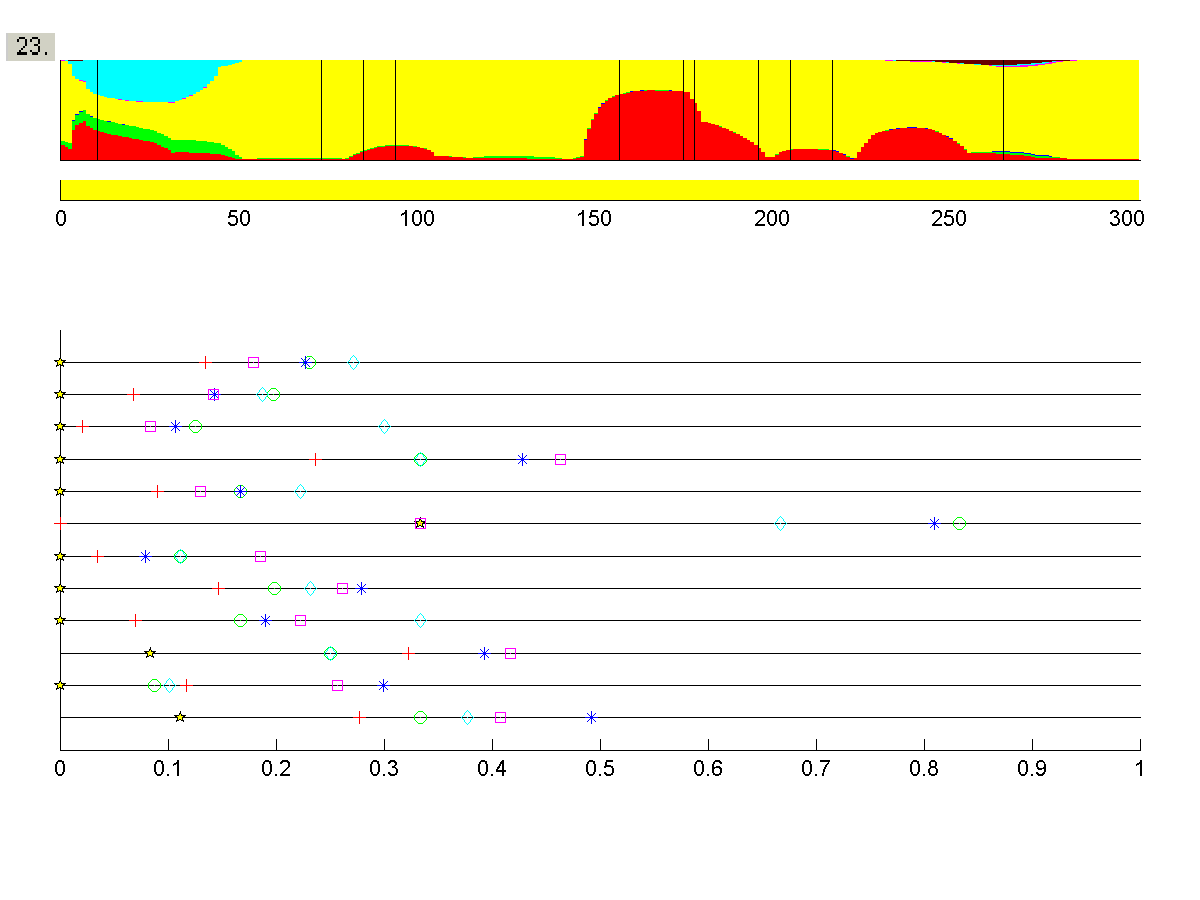

Supplement: Additional File 1 — Package of supplementary material. File BRAT_supplementary.zip available at includes a file BRAT_supplementary_text.pdf, which contains additional information on following issues: 1) elementary simulation experiments, 2) illustration of the effect of used parameter values and utilized approximations, and 3) a description of the real data analysis with more details than presented in the main text. The zip package also includes two folders. The folder "Coalescent_results" contains complete results for the analyzed coalescent data set, see the README file in the folder for further details. The folder "Trees for repetitive simulations" contains the figures of the left-side and right-side trees used in different types of simulations. [file 1471-2105-9-421-S1.zip › BRAT_supplementary_2/Coalescent_results/Results/strain23.png]

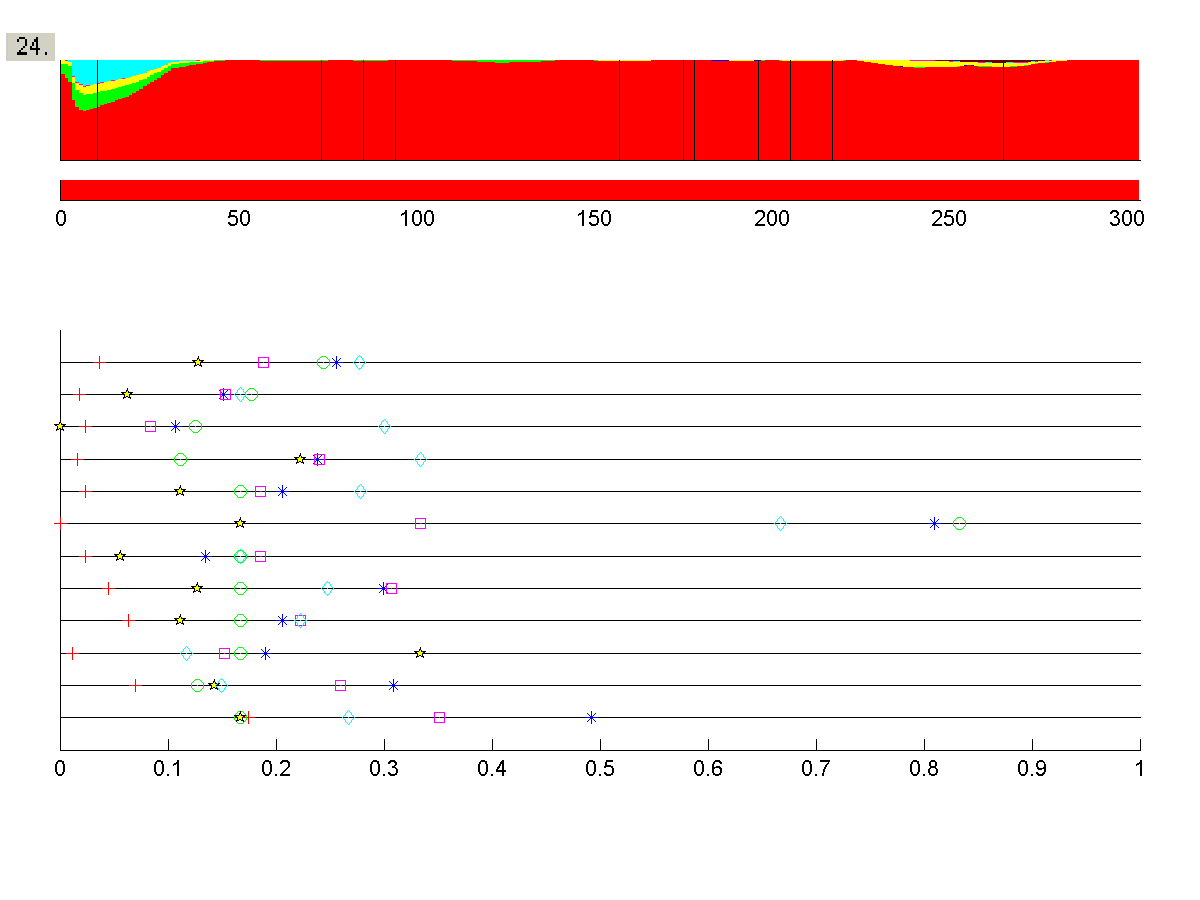

Supplement: Additional File 1 — Package of supplementary material. File BRAT_supplementary.zip available at includes a file BRAT_supplementary_text.pdf, which contains additional information on following issues: 1) elementary simulation experiments, 2) illustration of the effect of used parameter values and utilized approximations, and 3) a description of the real data analysis with more details than presented in the main text. The zip package also includes two folders. The folder "Coalescent_results" contains complete results for the analyzed coalescent data set, see the README file in the folder for further details. The folder "Trees for repetitive simulations" contains the figures of the left-side and right-side trees used in different types of simulations. [file 1471-2105-9-421-S1.zip › BRAT_supplementary_2/Coalescent_results/Results/strain24.png]

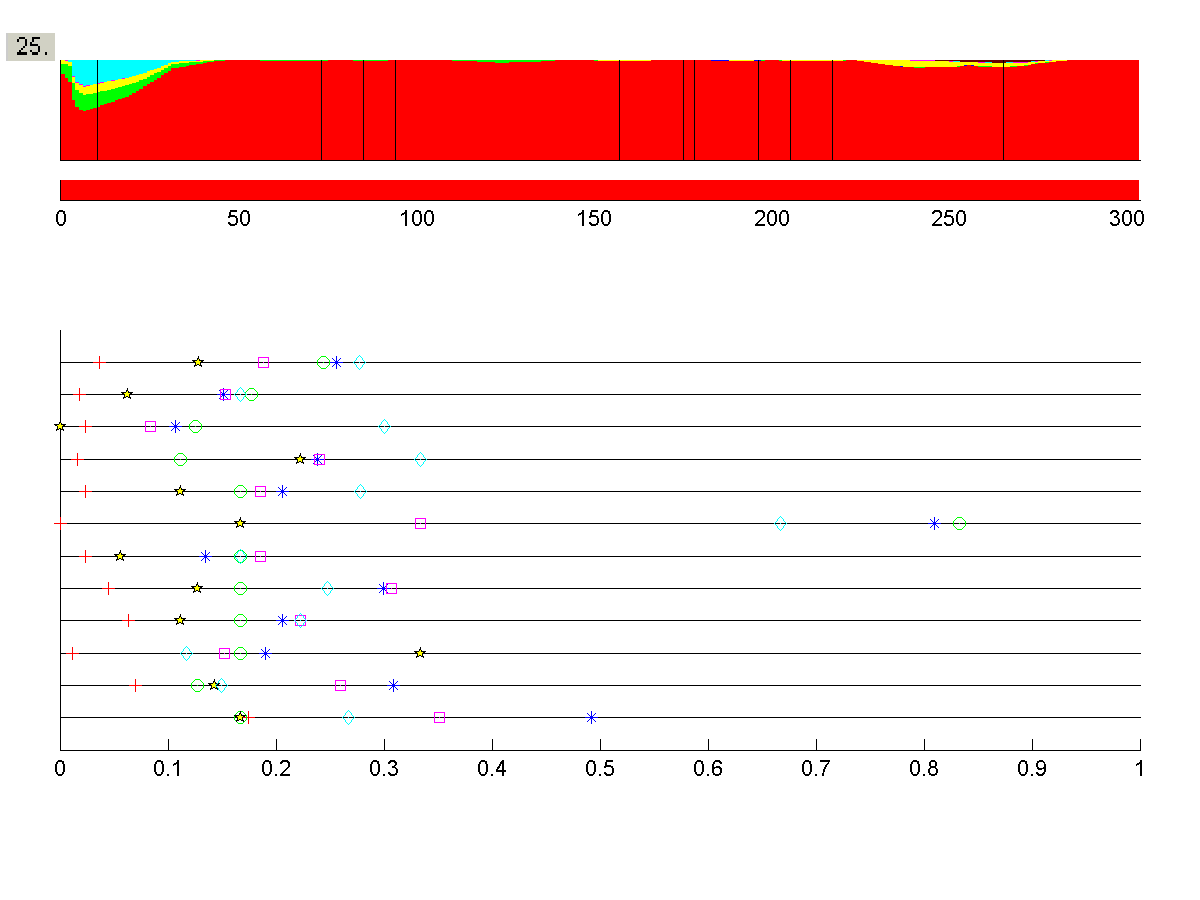

Supplement: Additional File 1 — Package of supplementary material. File BRAT_supplementary.zip available at includes a file BRAT_supplementary_text.pdf, which contains additional information on following issues: 1) elementary simulation experiments, 2) illustration of the effect of used parameter values and utilized approximations, and 3) a description of the real data analysis with more details than presented in the main text. The zip package also includes two folders. The folder "Coalescent_results" contains complete results for the analyzed coalescent data set, see the README file in the folder for further details. The folder "Trees for repetitive simulations" contains the figures of the left-side and right-side trees used in different types of simulations. [file 1471-2105-9-421-S1.zip › BRAT_supplementary_2/Coalescent_results/Results/strain25.png]

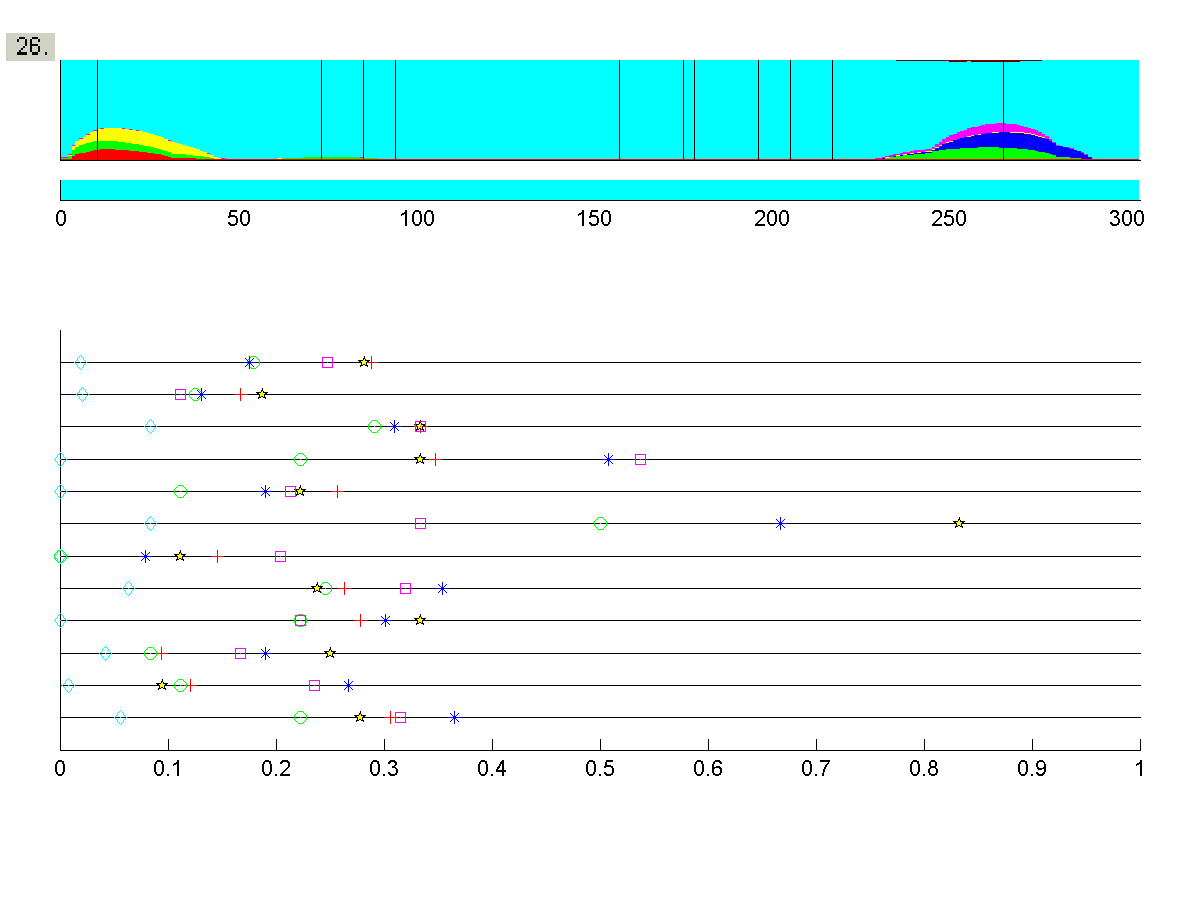

Supplement: Additional File 1 — Package of supplementary material. File BRAT_supplementary.zip available at includes a file BRAT_supplementary_text.pdf, which contains additional information on following issues: 1) elementary simulation experiments, 2) illustration of the effect of used parameter values and utilized approximations, and 3) a description of the real data analysis with more details than presented in the main text. The zip package also includes two folders. The folder "Coalescent_results" contains complete results for the analyzed coalescent data set, see the README file in the folder for further details. The folder "Trees for repetitive simulations" contains the figures of the left-side and right-side trees used in different types of simulations. [file 1471-2105-9-421-S1.zip › BRAT_supplementary_2/Coalescent_results/Results/strain26.png]

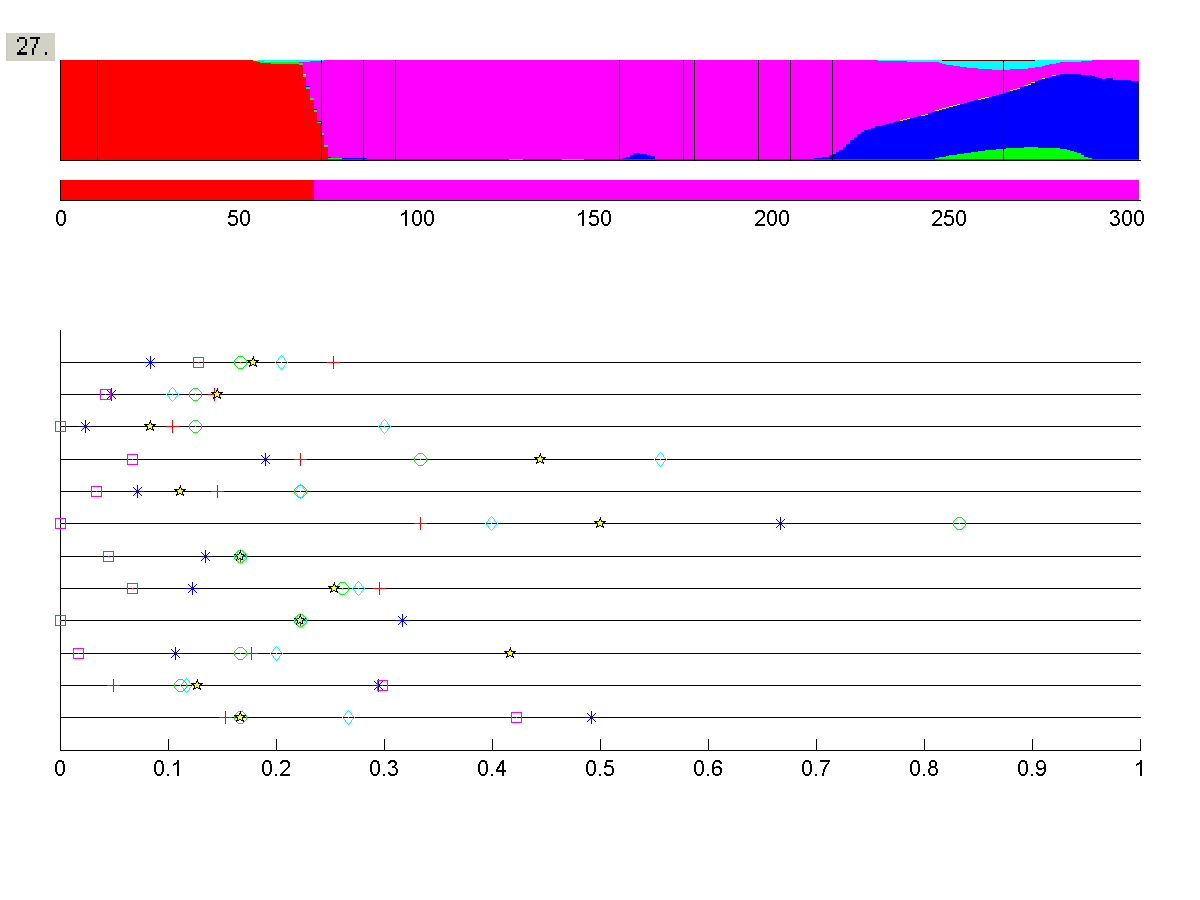

Supplement: Additional File 1 — Package of supplementary material. File BRAT_supplementary.zip available at includes a file BRAT_supplementary_text.pdf, which contains additional information on following issues: 1) elementary simulation experiments, 2) illustration of the effect of used parameter values and utilized approximations, and 3) a description of the real data analysis with more details than presented in the main text. The zip package also includes two folders. The folder "Coalescent_results" contains complete results for the analyzed coalescent data set, see the README file in the folder for further details. The folder "Trees for repetitive simulations" contains the figures of the left-side and right-side trees used in different types of simulations. [file 1471-2105-9-421-S1.zip › BRAT_supplementary_2/Coalescent_results/Results/strain27.png]

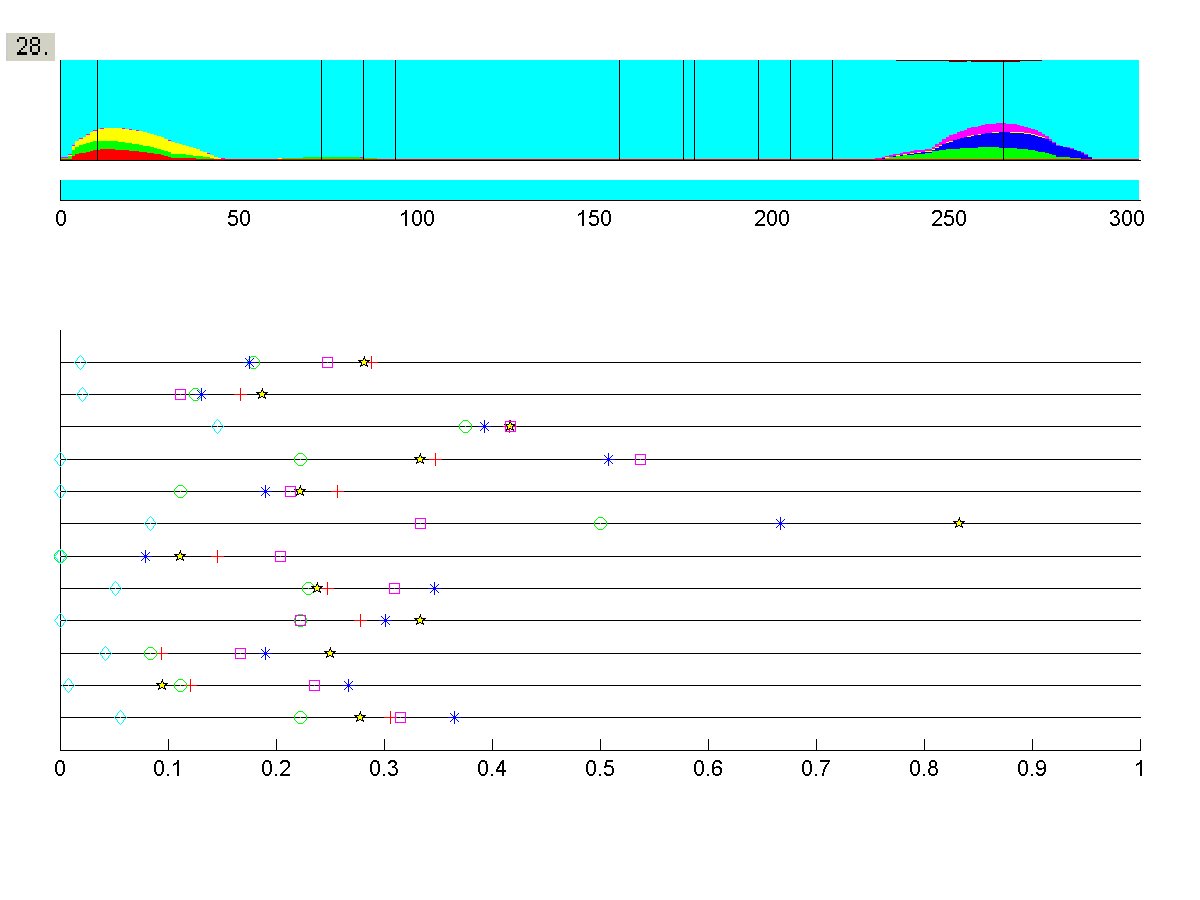

Supplement: Additional File 1 — Package of supplementary material. File BRAT_supplementary.zip available at includes a file BRAT_supplementary_text.pdf, which contains additional information on following issues: 1) elementary simulation experiments, 2) illustration of the effect of used parameter values and utilized approximations, and 3) a description of the real data analysis with more details than presented in the main text. The zip package also includes two folders. The folder "Coalescent_results" contains complete results for the analyzed coalescent data set, see the README file in the folder for further details. The folder "Trees for repetitive simulations" contains the figures of the left-side and right-side trees used in different types of simulations. [file 1471-2105-9-421-S1.zip › BRAT_supplementary_2/Coalescent_results/Results/strain28.png]

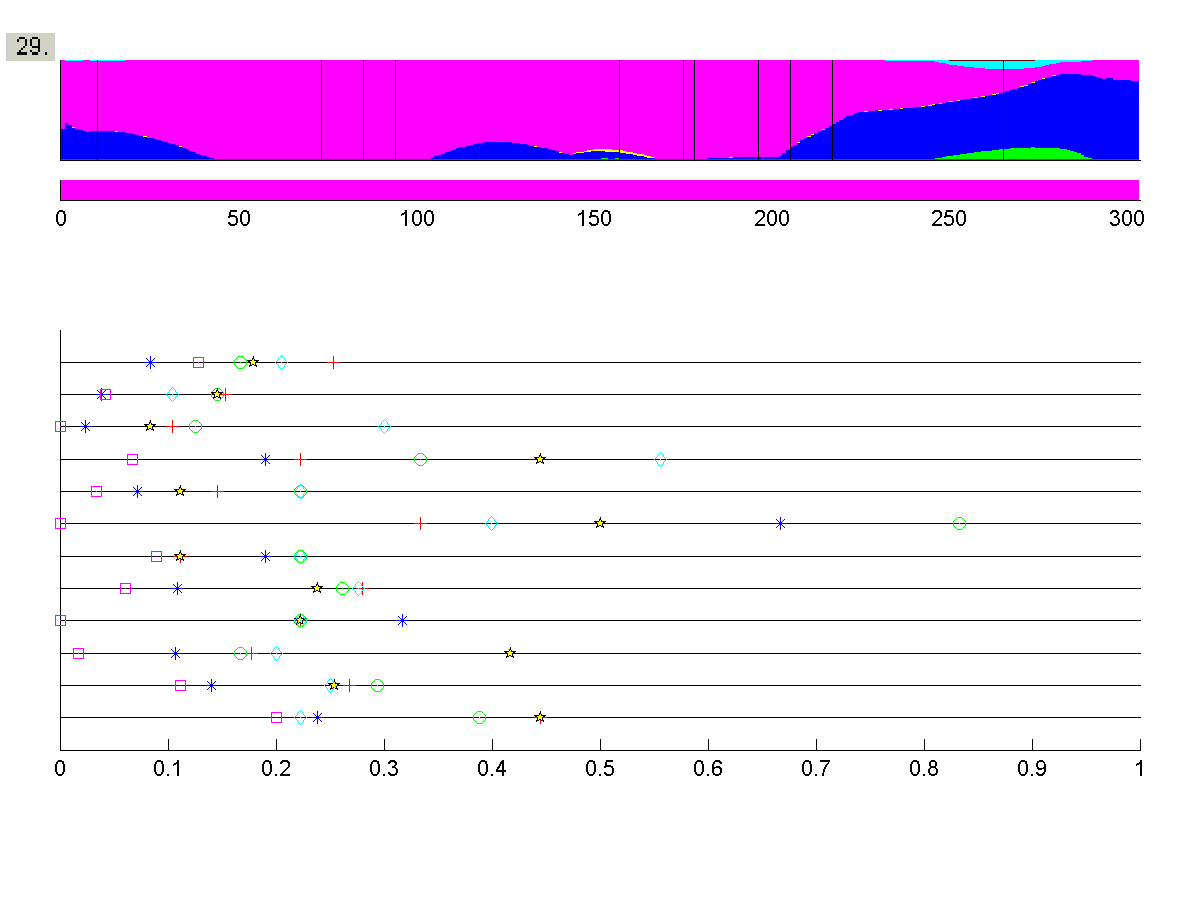

Supplement: Additional File 1 — Package of supplementary material. File BRAT_supplementary.zip available at includes a file BRAT_supplementary_text.pdf, which contains additional information on following issues: 1) elementary simulation experiments, 2) illustration of the effect of used parameter values and utilized approximations, and 3) a description of the real data analysis with more details than presented in the main text. The zip package also includes two folders. The folder "Coalescent_results" contains complete results for the analyzed coalescent data set, see the README file in the folder for further details. The folder "Trees for repetitive simulations" contains the figures of the left-side and right-side trees used in different types of simulations. [file 1471-2105-9-421-S1.zip › BRAT_supplementary_2/Coalescent_results/Results/strain29.png]

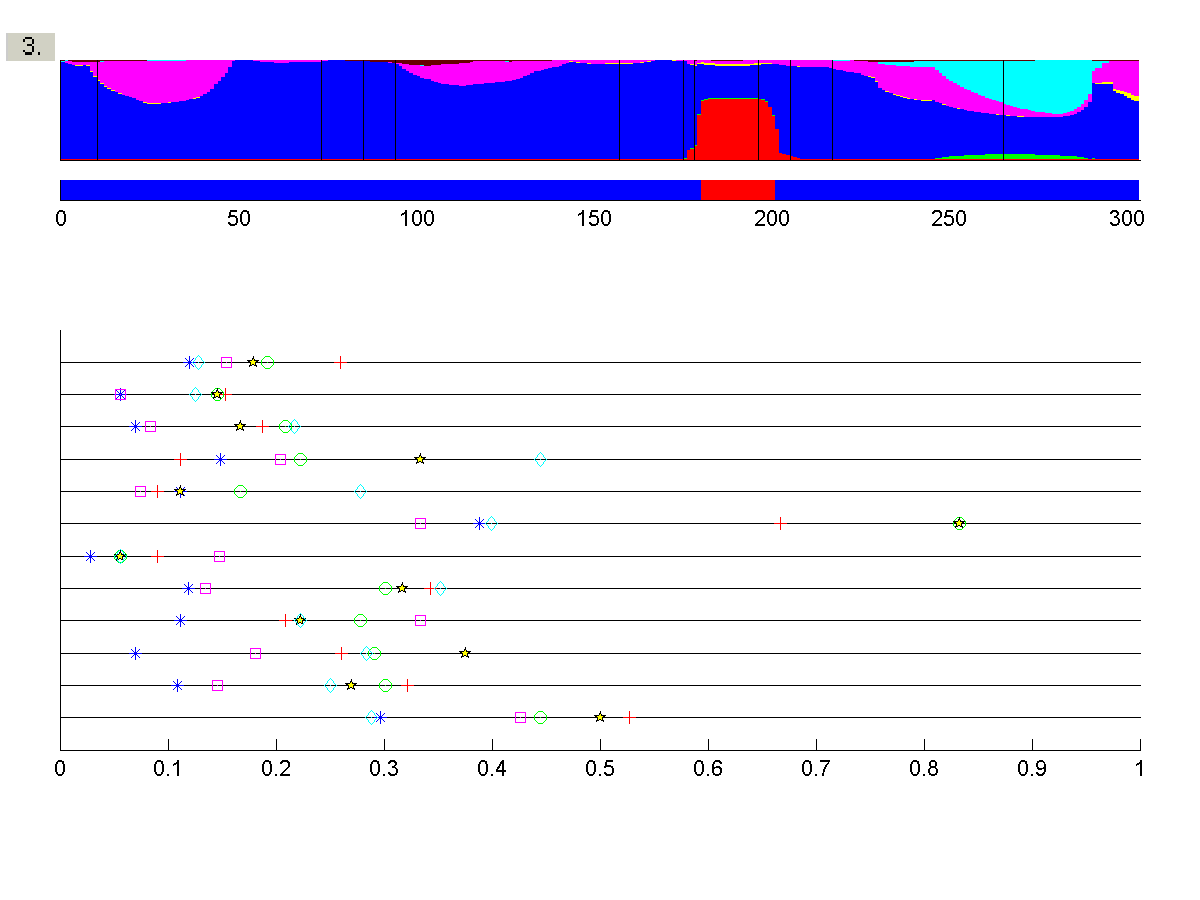

Supplement: Additional File 1 — Package of supplementary material. File BRAT_supplementary.zip available at includes a file BRAT_supplementary_text.pdf, which contains additional information on following issues: 1) elementary simulation experiments, 2) illustration of the effect of used parameter values and utilized approximations, and 3) a description of the real data analysis with more details than presented in the main text. The zip package also includes two folders. The folder "Coalescent_results" contains complete results for the analyzed coalescent data set, see the README file in the folder for further details. The folder "Trees for repetitive simulations" contains the figures of the left-side and right-side trees used in different types of simulations. [file 1471-2105-9-421-S1.zip › BRAT_supplementary_2/Coalescent_results/Results/strain3.png]

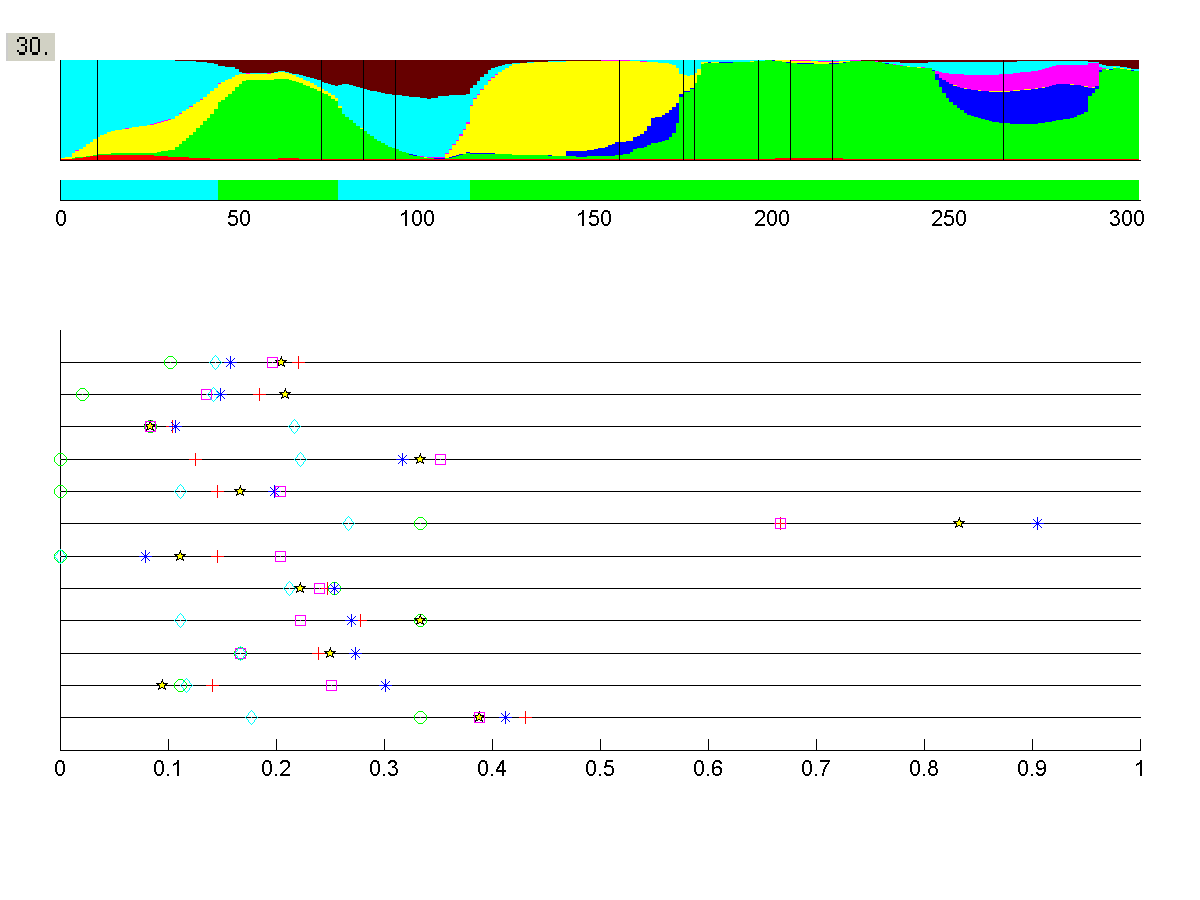

Supplement: Additional File 1 — Package of supplementary material. File BRAT_supplementary.zip available at includes a file BRAT_supplementary_text.pdf, which contains additional information on following issues: 1) elementary simulation experiments, 2) illustration of the effect of used parameter values and utilized approximations, and 3) a description of the real data analysis with more details than presented in the main text. The zip package also includes two folders. The folder "Coalescent_results" contains complete results for the analyzed coalescent data set, see the README file in the folder for further details. The folder "Trees for repetitive simulations" contains the figures of the left-side and right-side trees used in different types of simulations. [file 1471-2105-9-421-S1.zip › BRAT_supplementary_2/Coalescent_results/Results/strain30.png]

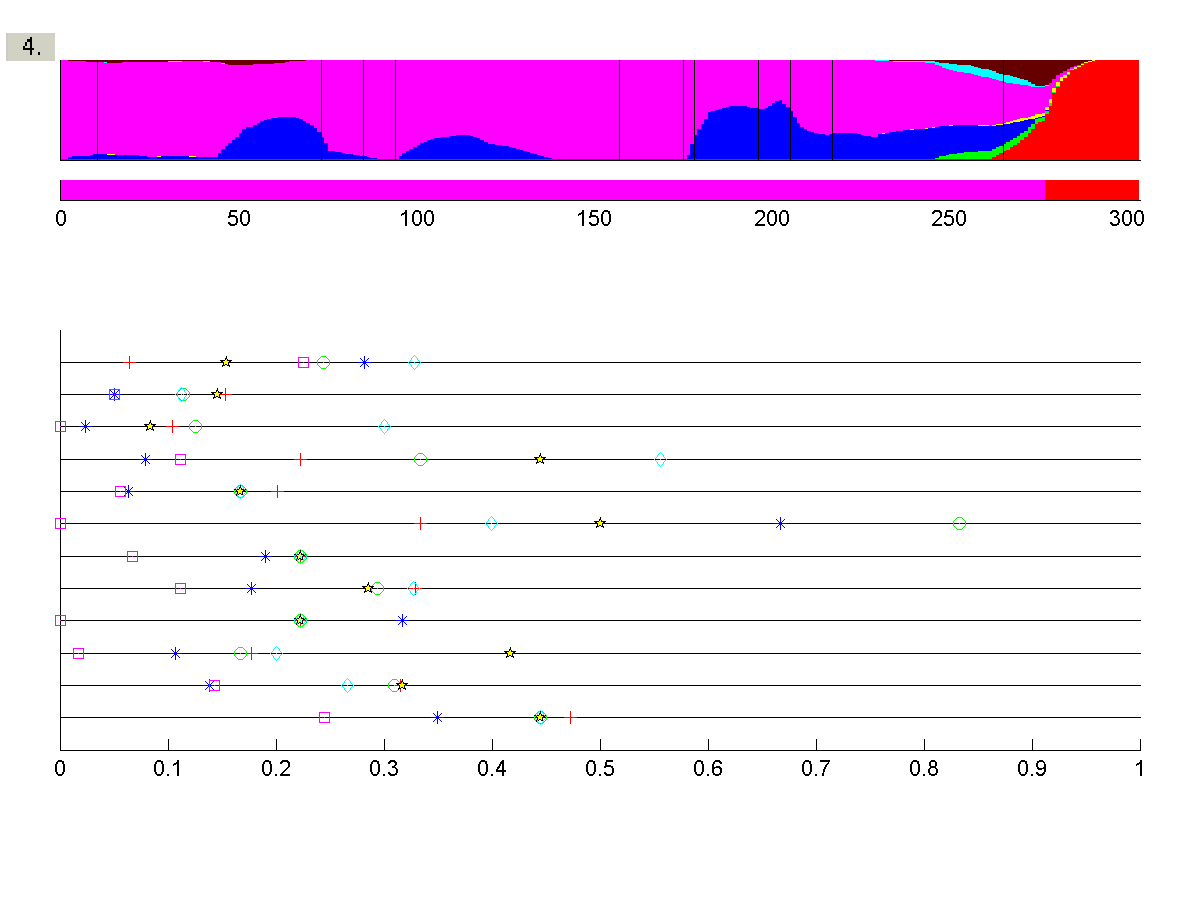

Supplement: Additional File 1 — Package of supplementary material. File BRAT_supplementary.zip available at includes a file BRAT_supplementary_text.pdf, which contains additional information on following issues: 1) elementary simulation experiments, 2) illustration of the effect of used parameter values and utilized approximations, and 3) a description of the real data analysis with more details than presented in the main text. The zip package also includes two folders. The folder "Coalescent_results" contains complete results for the analyzed coalescent data set, see the README file in the folder for further details. The folder "Trees for repetitive simulations" contains the figures of the left-side and right-side trees used in different types of simulations. [file 1471-2105-9-421-S1.zip › BRAT_supplementary_2/Coalescent_results/Results/strain4.png]

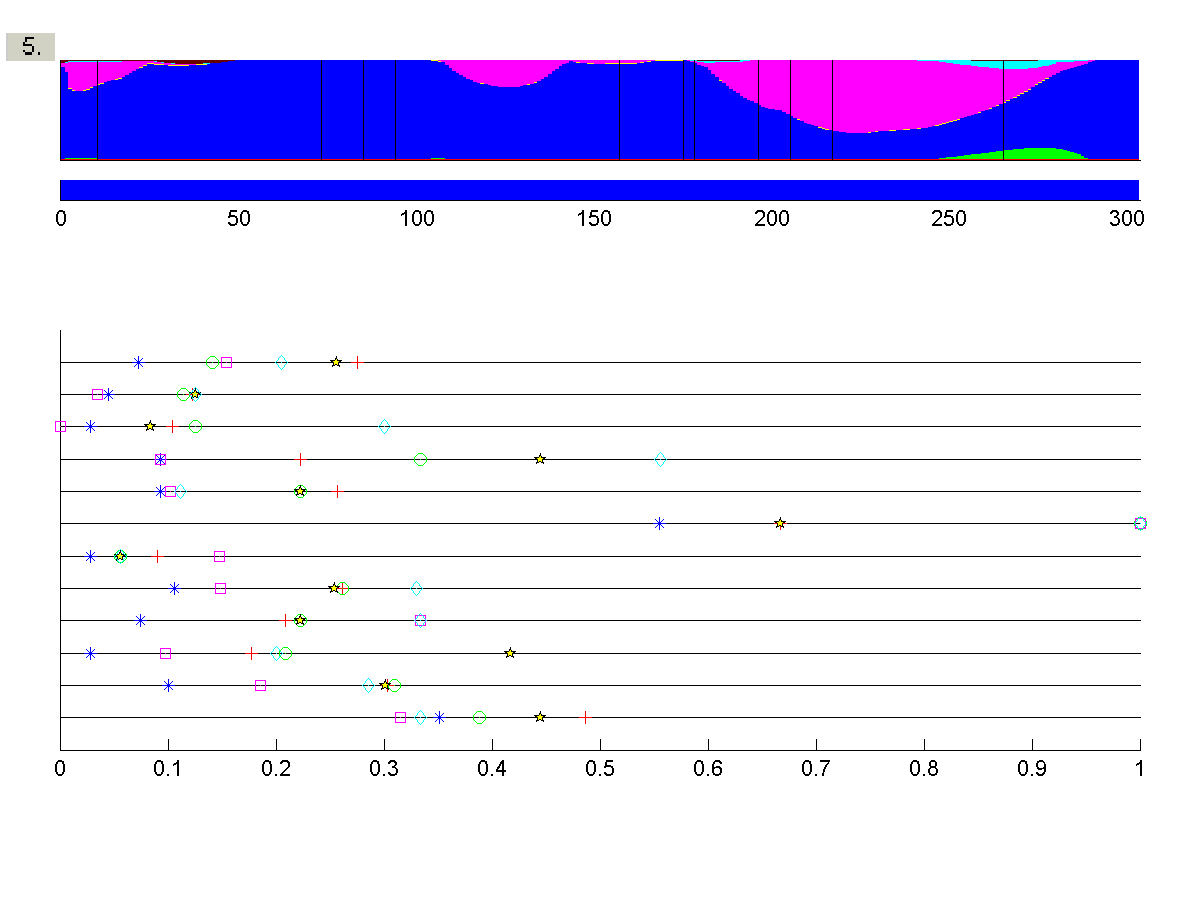

Supplement: Additional File 1 — Package of supplementary material. File BRAT_supplementary.zip available at includes a file BRAT_supplementary_text.pdf, which contains additional information on following issues: 1) elementary simulation experiments, 2) illustration of the effect of used parameter values and utilized approximations, and 3) a description of the real data analysis with more details than presented in the main text. The zip package also includes two folders. The folder "Coalescent_results" contains complete results for the analyzed coalescent data set, see the README file in the folder for further details. The folder "Trees for repetitive simulations" contains the figures of the left-side and right-side trees used in different types of simulations. [file 1471-2105-9-421-S1.zip › BRAT_supplementary_2/Coalescent_results/Results/strain5.png]

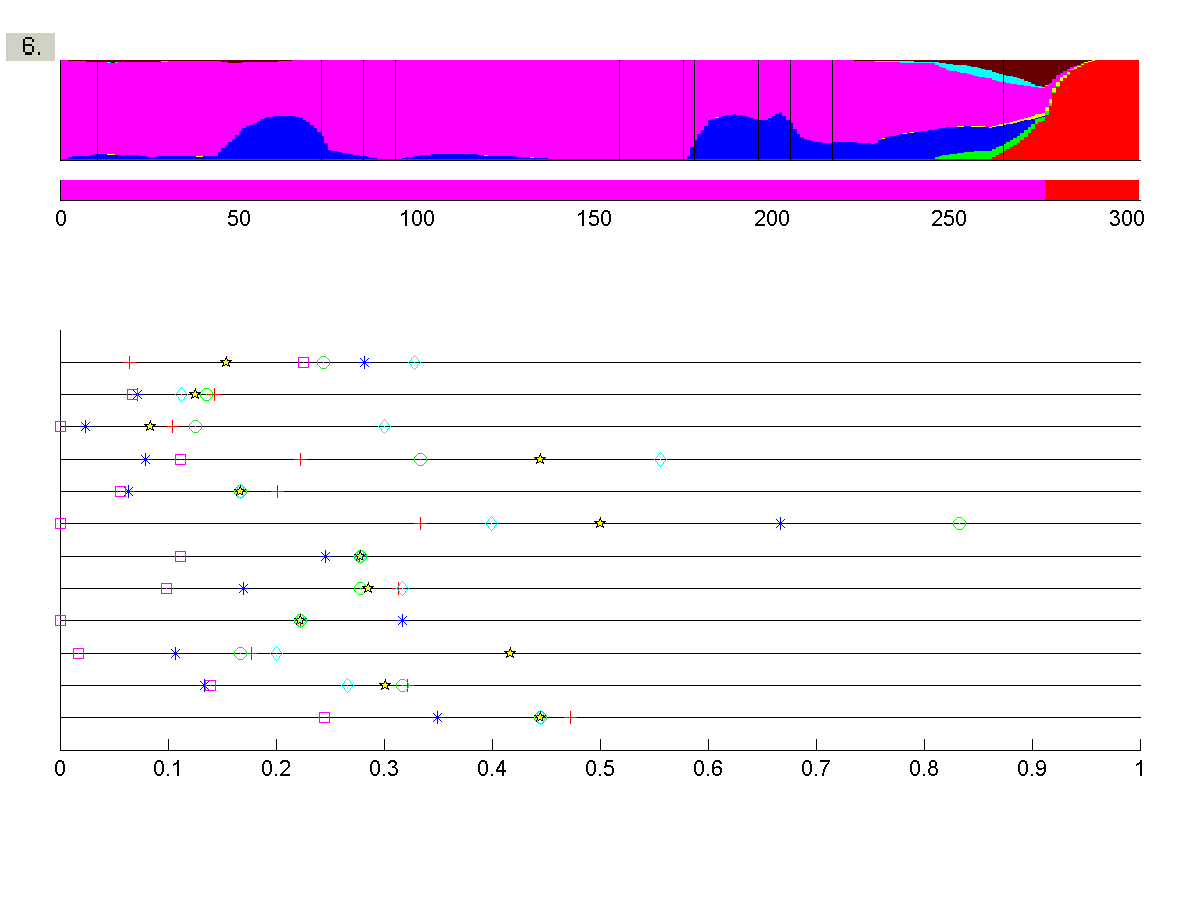

Supplement: Additional File 1 — Package of supplementary material. File BRAT_supplementary.zip available at includes a file BRAT_supplementary_text.pdf, which contains additional information on following issues: 1) elementary simulation experiments, 2) illustration of the effect of used parameter values and utilized approximations, and 3) a description of the real data analysis with more details than presented in the main text. The zip package also includes two folders. The folder "Coalescent_results" contains complete results for the analyzed coalescent data set, see the README file in the folder for further details. The folder "Trees for repetitive simulations" contains the figures of the left-side and right-side trees used in different types of simulations. [file 1471-2105-9-421-S1.zip › BRAT_supplementary_2/Coalescent_results/Results/strain6.png]

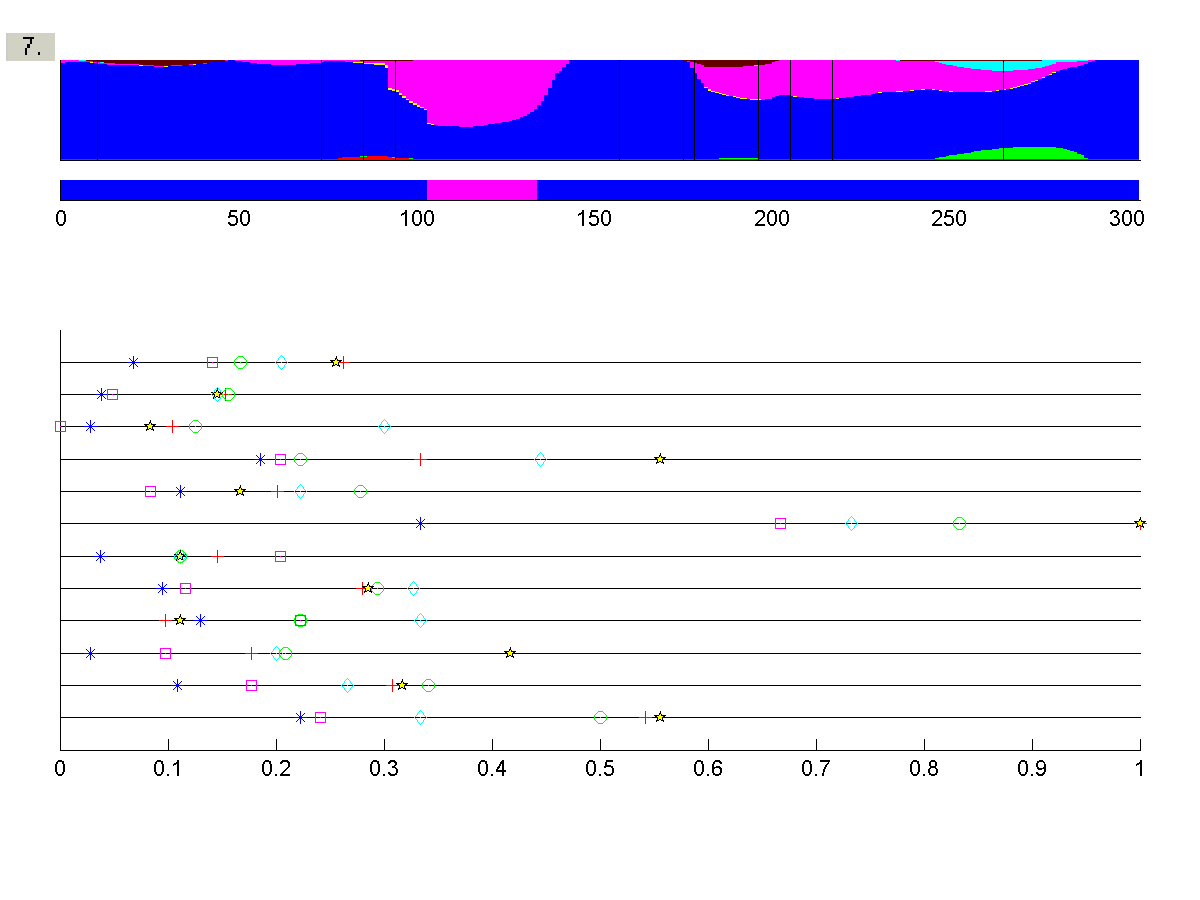

Supplement: Additional File 1 — Package of supplementary material. File BRAT_supplementary.zip available at includes a file BRAT_supplementary_text.pdf, which contains additional information on following issues: 1) elementary simulation experiments, 2) illustration of the effect of used parameter values and utilized approximations, and 3) a description of the real data analysis with more details than presented in the main text. The zip package also includes two folders. The folder "Coalescent_results" contains complete results for the analyzed coalescent data set, see the README file in the folder for further details. The folder "Trees for repetitive simulations" contains the figures of the left-side and right-side trees used in different types of simulations. [file 1471-2105-9-421-S1.zip › BRAT_supplementary_2/Coalescent_results/Results/strain7.png]

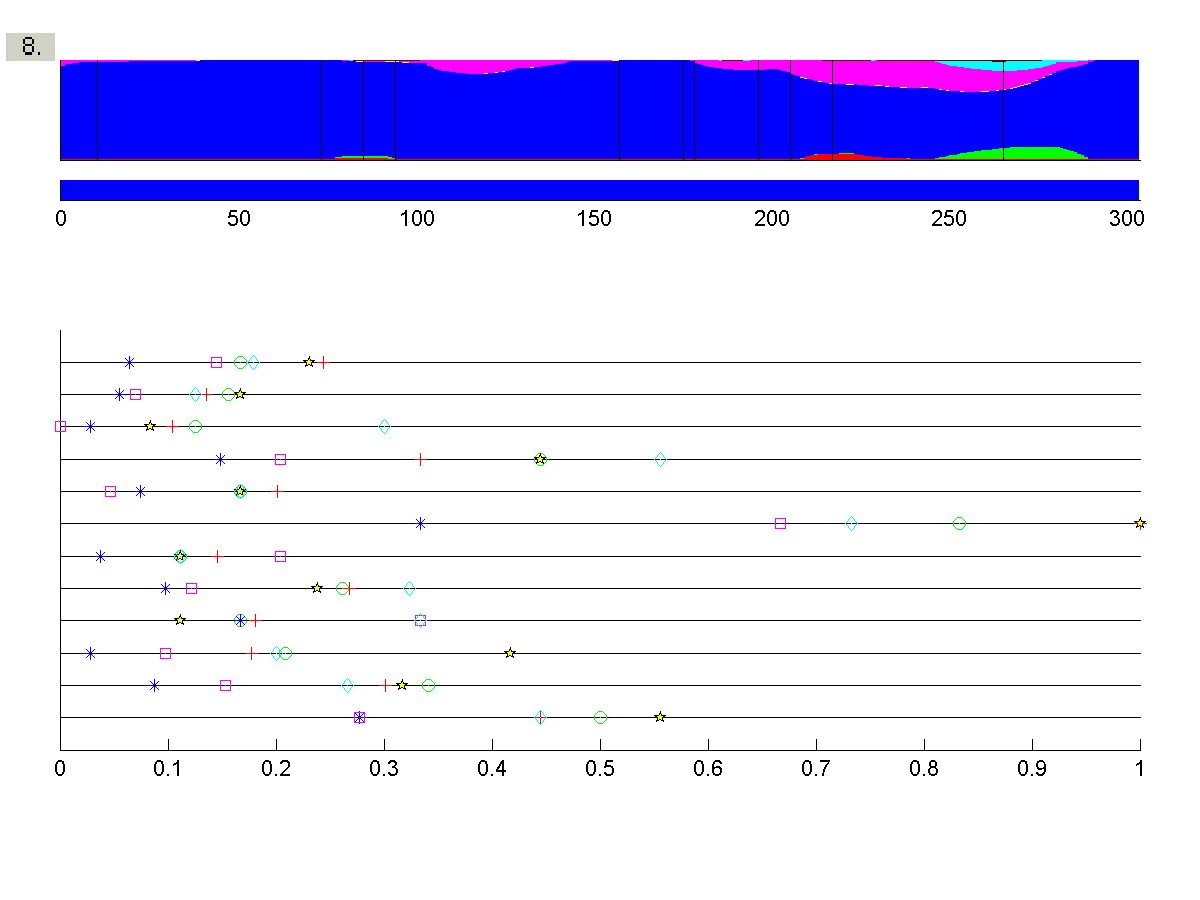

Supplement: Additional File 1 — Package of supplementary material. File BRAT_supplementary.zip available at includes a file BRAT_supplementary_text.pdf, which contains additional information on following issues: 1) elementary simulation experiments, 2) illustration of the effect of used parameter values and utilized approximations, and 3) a description of the real data analysis with more details than presented in the main text. The zip package also includes two folders. The folder "Coalescent_results" contains complete results for the analyzed coalescent data set, see the README file in the folder for further details. The folder "Trees for repetitive simulations" contains the figures of the left-side and right-side trees used in different types of simulations. [file 1471-2105-9-421-S1.zip › BRAT_supplementary_2/Coalescent_results/Results/strain8.png]

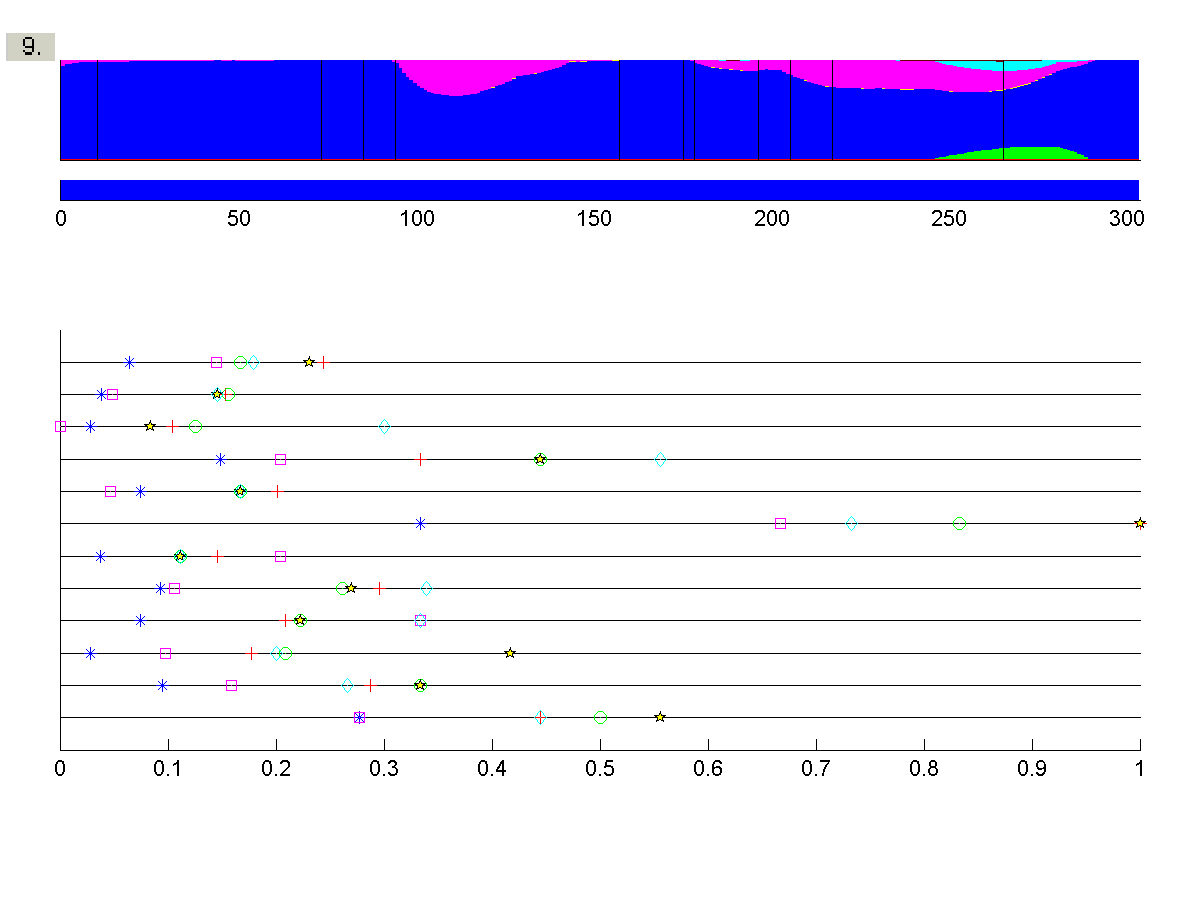

Supplement: Additional File 1 — Package of supplementary material. File BRAT_supplementary.zip available at includes a file BRAT_supplementary_text.pdf, which contains additional information on following issues: 1) elementary simulation experiments, 2) illustration of the effect of used parameter values and utilized approximations, and 3) a description of the real data analysis with more details than presented in the main text. The zip package also includes two folders. The folder "Coalescent_results" contains complete results for the analyzed coalescent data set, see the README file in the folder for further details. The folder "Trees for repetitive simulations" contains the figures of the left-side and right-side trees used in different types of simulations. [file 1471-2105-9-421-S1.zip › BRAT_supplementary_2/Coalescent_results/Results/strain9.png]
